# Supplementary material for: Inhibiting ACK1-mediated phosphorylation of C-terminal Src kinase counteracts prostate cancer immune checkpoint blockade resistance
Source: Nat Commun. 2022 Nov 14;13:6929. doi: 10.1038/s41467-022-34724-5 (PMC9663509; doi:10.1038/s41467-022-34724-5)
Supplement: Supplementary file 1 — Supplementary Information [file 41467_2022_34724_MOESM1_ESM.pdf]

SUPPLEMENTARY DATA

Inhibiting ACK1-mediated phosphorylation of C-terminal Src kinase counteracts prostate cancer immune checkpoint blockade resistance

Dhivya Sridaran[1,2,#], Surbhi Chouhan[1,2], Kiran Mahajan[1,2,3,#], Arun Renganathan[1,2], Cody Weimholt[1,4,5], Shambhavi Bhagwat[1,2], Melissa Reimers[3,4], Eric H. Kim[1,2], Manish K. Thakur[6], M. A. Saeed[3], Russell K. Pachynski[3,4], Markus A. Seeliger[6,7], W. Todd Miller[7, 8], Felix Y. Feng[9,10,11,12], Nupam P. Mahajan[1,2,3,*]

Contents

Supplementary Fig. 1: ACK1 functional domains and generation of *Ack1* knockout mice.

Supplementary Fig. 2: Increased CD8 T cells activation in *Ack1*/*Tnk2* knockout mice.

Supplementary Fig. 3: Immunophenotyping in WT and *Ack1/Tnk2* KO mice.

Supplementary Fig. 4. Immune activation and extent of autoimmunity in WT and *Ack1* knockout mice

Supplementary Fig. 5: Identification of CSK and PAG Tyr-phosphorylation sites and evolutionary conservation of Tyr18 site of CSK.

Supplementary Fig. 6: Characterization of antibodies against pTyr18-CSK and X-ray co-crystal structure of ACK1 kinase domain bound to (*R*)-**9b**.

Supplementary Fig. 7: T cell activation upon loss of ACK1 causes suppression of prostate tumor growth.

Supplementary Fig. 8: Alterations in the T cell sub-types upon loss of ACK1 dampening tumor growth

Supplementary Fig. 9: ACK1 inhibitor (*R*)-9b causes T cell activation in TRAMP-C2 tumor bearing immunocompetent mice.

Supplementary Fig. 10: T cell activation upon pharmacological inhibition of ACK1.

Supplementary Fig. 11: Overcoming T cell exhaustion upon pharmacological inhibition of ACK1.

Supplementary Fig. 12: Increased effector memory T cell levels upon genetic and pharmacological inhibition of ACK1 activity.

Supplementary Fig. 13: Effect of antibody depletion and adoptive transfer of T cells from A*ck1* KO and WT mice on TRAMP-C2 tumor growth.

Supplementary Fig. 14: Persistence of T cells upon ACK1 loss and effect of ICB and ACK1 inhibition on TRAMP-C2 tumor growth

Supplementary Fig. 15: ICB hampers immune surveillance of prostate tumors by increasing Tregs.

Supplementary Fig. 16: ACK inhibition improves immune infiltration in prostate tumors

Supplementary Fig. 17: Effective cancer cell killing and decrease in PAG Tyr-phosphorylation upon loss of ACK1 kinase activity.

Supplementary Table 1: X-ray data collection and refinement statistics.

Supplementary Table 2: List of primers

Supplementary Fig. 1

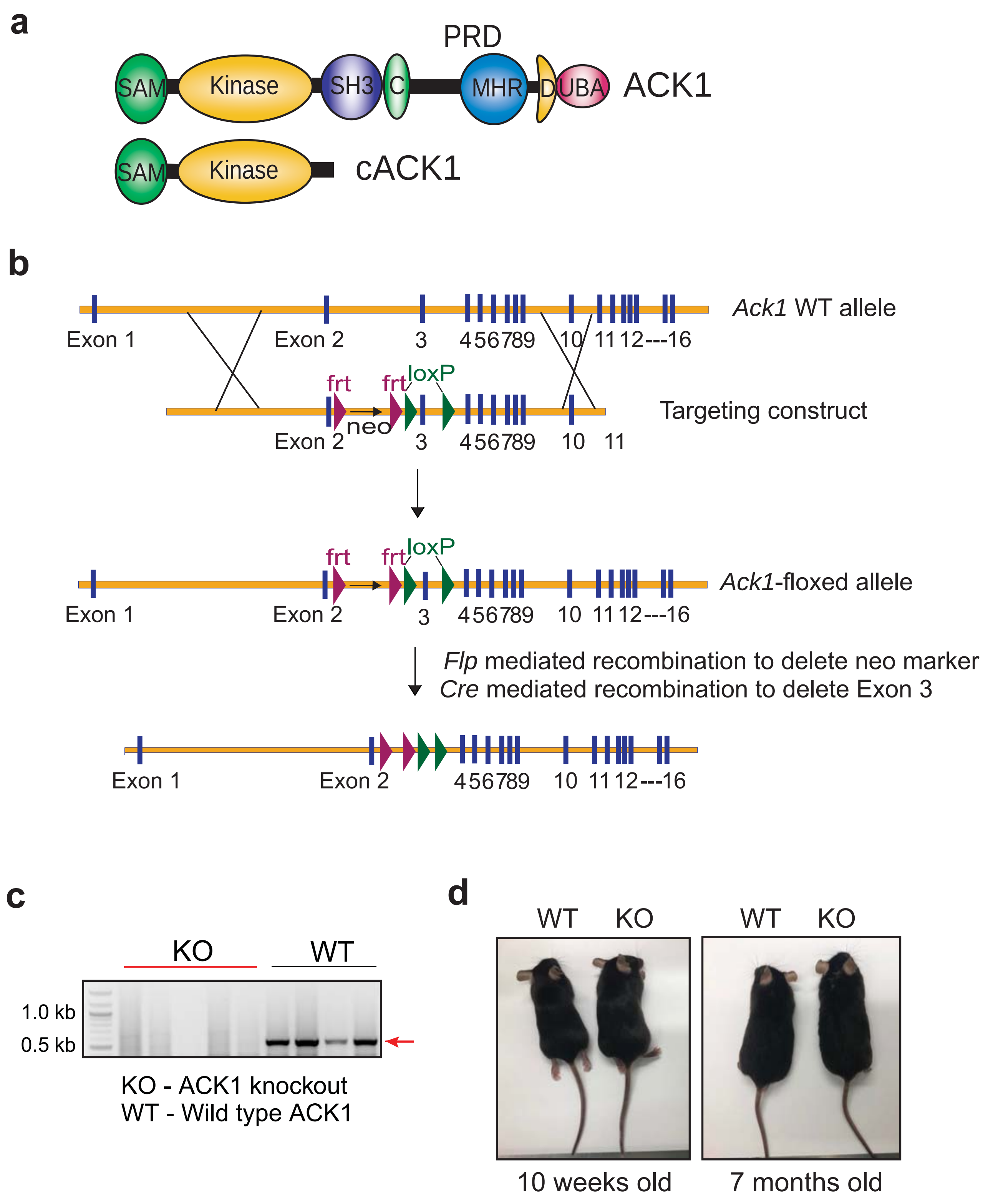

Supplementary Fig. 1: ACK1 functional domains and generation of *Ack1* knockout mice.

a Schematic representation of ACK1 and the deletion construct, a HA-tagged deletion mutant, cACK1. SAM, Sterile alpha motif; Kinase, kinase domain; SH3, Src homology domain 3; C, Cdc42 Rac interactive binding domain; PRD, Proline rich domain that contain MHR, Mig6 homology region; D, Degron Motif; UBA, Ubiquitin binding domain. **b** Generation of *Ack1* KO mice. Targeted disruption strategy to delete the exon 3 of the Ack1 gene, to cause premature translational termination. Shown are the wild-type ACK1 allele with indicated exons (top), the targeting vector (middle), and the floxed allele (bottom). The locations of the loxP sites used for cre-mediated homologous recombination are depicted by green arrows. **c** Genotyping of WT and *Ack1* KO mice by tail PCR. Representative image from three biologically independent experiments is shown. **d** 10 weeks and 7 months old WT and *Ack1* KO mice are shown. Source data are provided as a Source Data file.

Supplementary Fig. 2

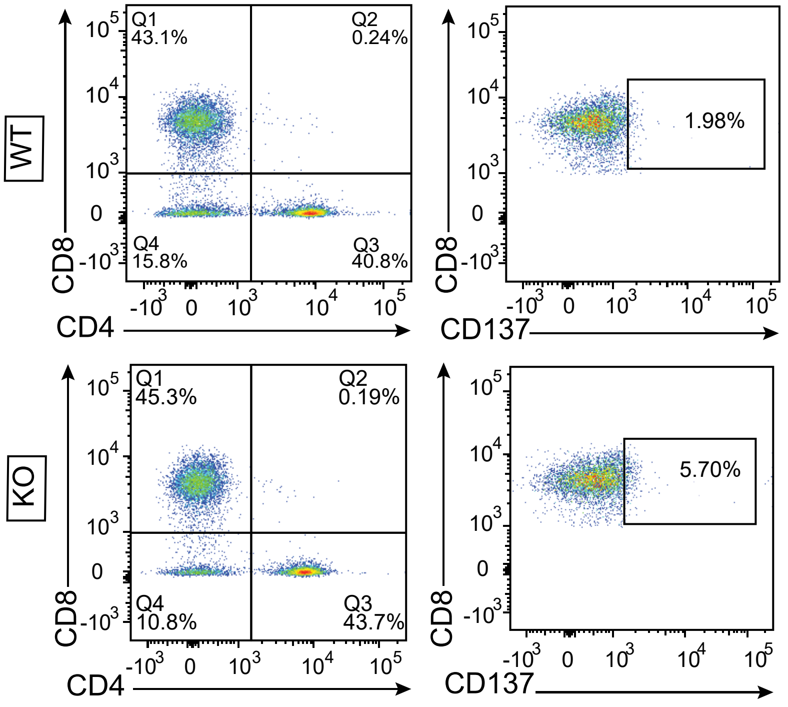

Supplementary Fig. 2: Increased CD8 T cells activation in *Ack1* knockout mice.

A representative flow cytometric analysis of CD137 on CD8 gated population isolated from splenocytes of WT and *Ack1/Tnk2* KO mice implanted with TRAMP-C2 cells. Source data are provided as a Source Data file.

Supplementary Fig. 3

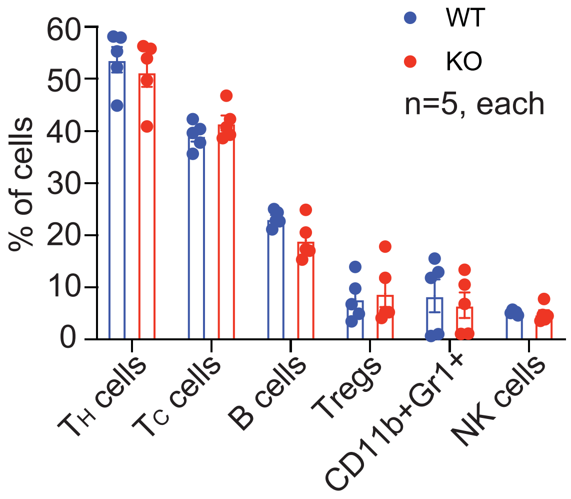

Supplementary Fig. 3: Immunophenotyping in WT and *Ack1* KO mice.

The percentages of Foxp3^{-}CD4^{+} helper T, Foxp3^{+}CD4^{+} regulatory T (Treg), CD8^{+} CTLs, CD19^{+} B, NK1.1^{+} NK, and CD11b^{+}Gr-1^{+} myeloid cells are shown (n=5 mice in each group). Data are represented as mean ± SEM and p values were determined by unpaired two-tailed Student's t-test. Source data are provided as a Source Data file.

Supplementary Fig. 4

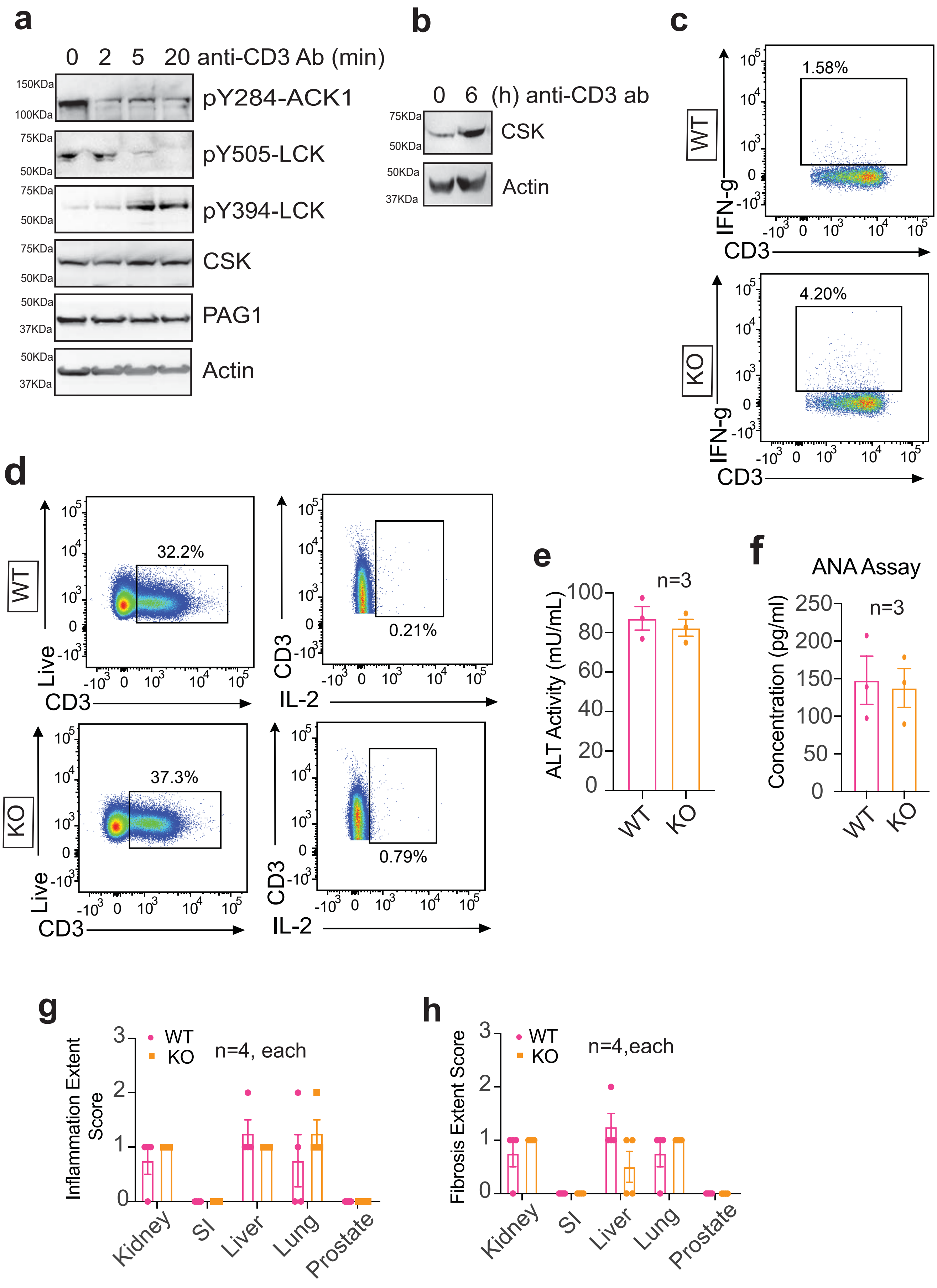

Supplementary Fig. 4: Immune activation and extent of autoimmunity in WT and *Ack1* knockout mice.

a and **b** Immunoblotting of Jurkat cells treated with anti-CD3 antibody. Representative images are shown (n = 3 biologically independent experiments). **c** and **d** Representative scatter plots of IFN-γ (**c**) and IL-2 (**d**) expression in the CD3 population isolated from the splenocytes of WT and *Ack1* KO mice is shown. (n = 3 biologically independent experiments). **e** Liver lysates were made from the WT and *Ack1* KO mice and ALT activity was assessed (n=3 mice in each group). **f** Anti-nuclear antibody assay was done in the serum of WT and *Ack1* KO mice (n=3 mice in each group). **g** and **h** Inflammation and fibrosis scores in the organs from WT and *Ack1* KO mice as assessed by H&E and picrosirius red staining of the histological sections (n=4 mice in each group). The inflammation extent score was given based on the % tissue involved (H&E) and fibrosis extent score was based on % tissue involved (Picrosirius Red) where, 0 = none, 1= ≤ 5%, 2 = 6-10%, 3 = 11-33%, 4 = 34-66%, 5 = ≥ 67%. For **e-h**, the data are represented as mean ± SEM and p values were determined by unpaired two-tailed Student's t-test. Source data are provided as a Source Data file.

Supplementary Fig. 5

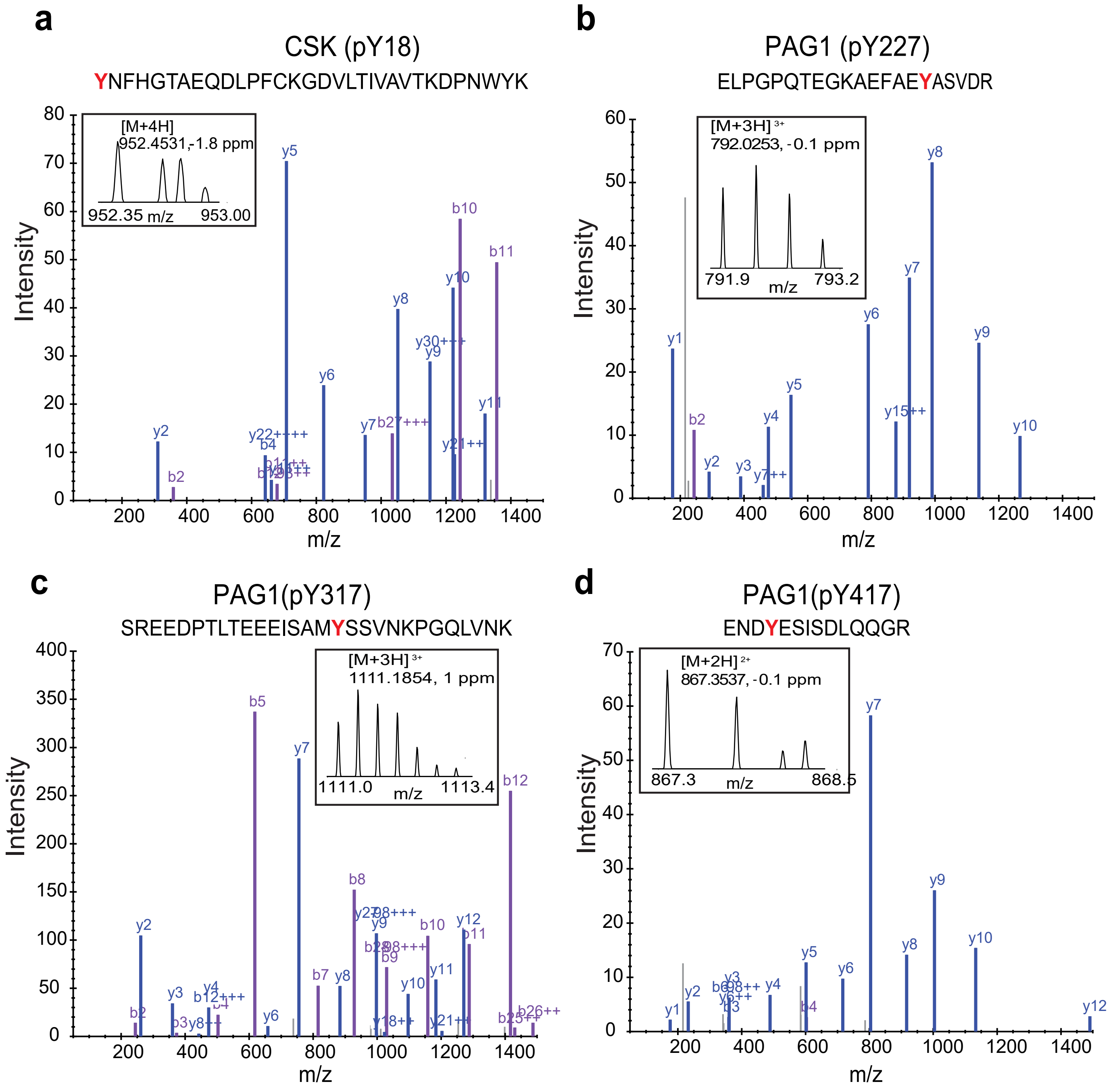

e

Tyr 18

```
PSGTECIAKYNFHGTAEQDLPFCKGDVLTIVAVTKDPNWYKAKNKV GREGIIPANYVQKREG CSK(9-70)
GGVTTFVALYDYESRTETDLSFKKGERLQIVNNTEG-DWWLAHSLSTGQTGYIPSNYVAPSDS SRC(84-145)
EQGDIVVALYPYDGIHPDDLSFKKGEKMKVLEE-HG-EWWKAKSLLTKKEGFIPSNYVAKLNT LYN(63-123)
TGVTLFVALYDYEARTEDDLSFHKGEKFQILNSSEG-DWWEARSLTTGETGYIPSNYVAPVDS FYN(82-143)
GGVTIFVALYDYEARTTEDLSFKKGERFQIINNTEG-DWWEARSIATGKNGYIPSNYVAPADS YES(91-152)
SEDIIVVALYDYEAIHHEDLSFQKGDQMVVLEE-SG-EWWKARSLATRKEGYIPSNYVARVDS HCK(78-138)
IGVTLFIALYDYEARTEDDLTFTKGEKFHILNNTEG-DWWEARSLSSGKTGCIPSNYVAPVDS FGR(77-138)
EDKHFVVALYDYTAMNDRDLQMLKGEKLQVLKGTG  DWWLARSLVTGREGYVPSNFVARVES BLK(58-118)
LQDNLVIALHSYEPSHDGDLGFEKGEQLRILEQ-SG-EWWKAQSLTTGQEGFIPFNFVAKANS LCK(61-121)
```

Supplementary Fig. 5: Identification of CSK and PAG Tyr-phosphorylation sites and evolutionary conservation of Tyr18 site in CSK.

a HEK293T cells were transfected with ACK1 and 48 hr post-transfection, cell lysates were processed for LC-MS/MS analysis. A peptide **Y**NFHGTAEQDLPFCKGDVLTIVAVTKDPNWYK from CSK (pY18) was detected at m/z 952.4531 (4+), which represents mass accuracy of -1.8 ppm (MS1). MS/MS spectrum confirmed the peptide sequence. The identification was made with MaxQuant software. **b** Peptide ELPGPQTEGKAEFAE**Y**ASVDR from PAG1 (pY227) was detected at m/z 792.0253 (3+), which represents mass accuracy of -0.1 ppm (MS1). MS/MS spectrum confirmed the peptide sequence. The identification was made with MaxQuant software. **c** A peptide SREEDPTLTEEEISAM**Y**SSVNKPGQLVNK from PAG1 (pY317) was detected at m/z 1111.1854 (3+), which represents mass accuracy of 1 ppm (MS1). MS/MS spectrum confirmed the peptide sequence. The identification was made with MaxQuant software. **d** Peptide END**Y**ESISDLQQGR from PAG1 (pY417) was detected at m/z 867.3537 (2+), which represents mass accuracy of -0.1 ppm (MS1). MS/MS spectrum confirmed the peptide sequence. The identification was made with MaxQuant software. **e** Alignment of SH3 domains of SRC family of kinases. Tyr18, shown in red, is conserved in all the kinases except LCK. Other conserved residues in SH3 domain are highlighted in yellow. The amino acids corresponding to each SH3 domains is shown in brackets next to the protein.

Supplementary Fig. 6

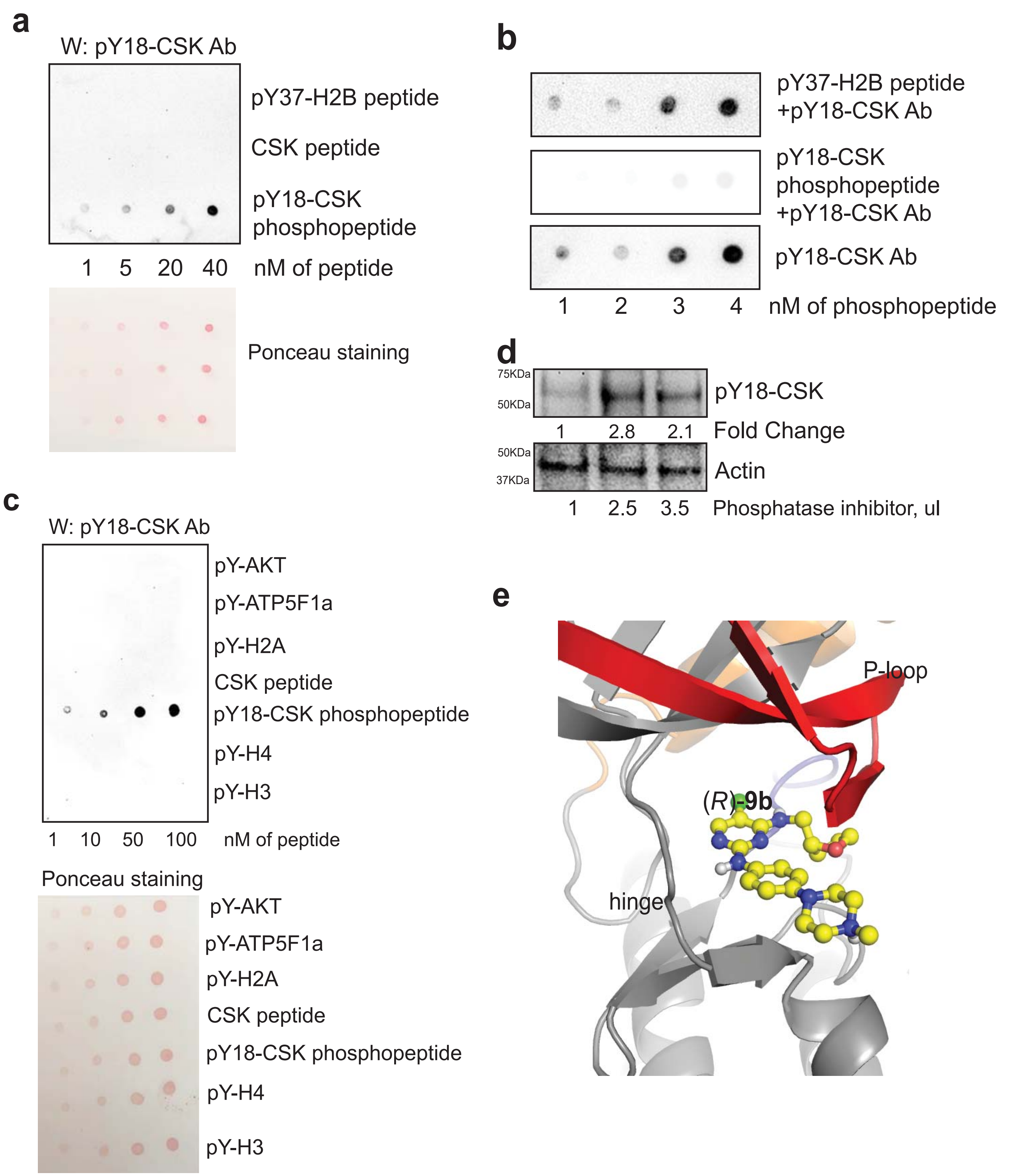

f

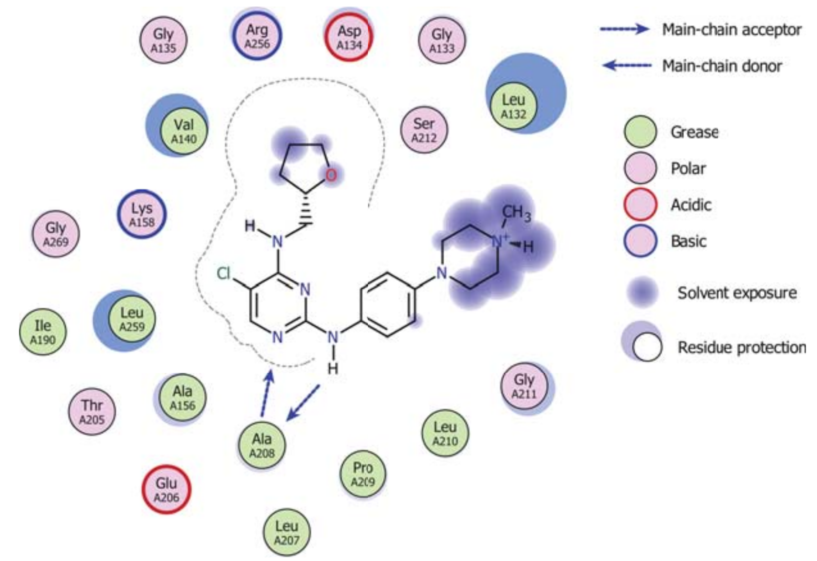

g

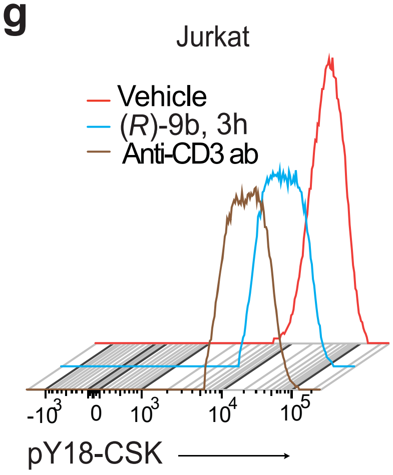

h

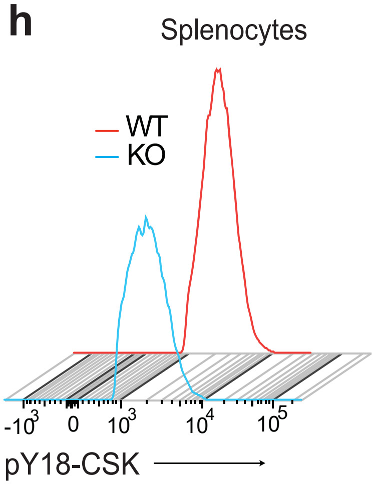

Supplementary Fig. 6: Characterization of antibodies against pTyr18-CSK and X-ray co-crystal structure of ACK1 kinase domain bound to (R)-9b.

a Validation of pY18-CSK antibody. Peptide spanning Y18 site, IAKpYNFHGTAEQDL and identical but unmodified peptide IAKYNFHGTAEQDL were spotted on nitrocellulose membrane (in increasing concentration) followed by immunoblotting with pY18-CSK antibody. A phospho-peptide derived from histone H2B is used as a negative control (top). **b** Peptide were spotted on nitrocellulose membrane in increasing concentration followed by immunoblotting with pY18-CSK antibody (bottom panel). Prior to probing, the pY18-CSK antibody was pre-incubated with the CSK ((middle panel) or histone H2B (top panel) phosphopeptide. **c** Peptides (as indicated) were spotted on nitrocellulose membrane in increasing concentration followed by immunoblotting with pY18-CSK antibody. **d** Jurkat cells were treated with different concentrations of phosphatase inhibitor cocktail followed by immunoblotting with pY18-CSK antibody. Representative images are shown (n = 3 biologically independent experiments). **e** X-ray co-crystal structure of ACK1 kinase domain bound to (R)-9b. (R)-9b binds to the ATP binding pocket. **f** A schematic representation of the interactions between (R)-9b and ACK1 kinase. **g** and **h** A representative flow cytometry analysis of pY-CSK18 levels in Jurkat cells treated with (R)-9b or anti-CD3 antibody (g) and splenocytes isolated from WT and KO mice

Supplementary Fig. 7

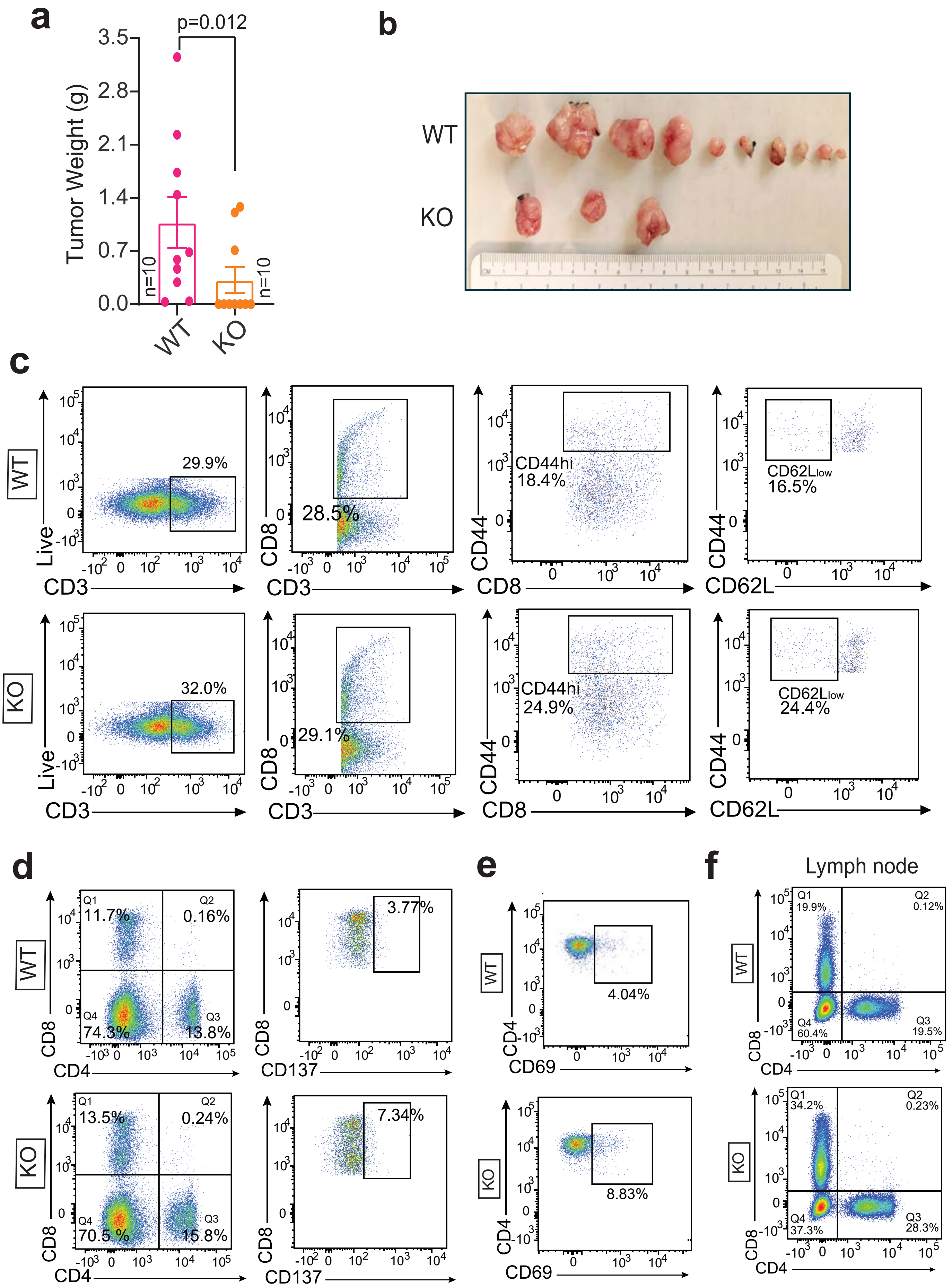

Supplementary Fig. 7: T cell activation upon loss of *Ack1* causes suppression of prostate tumor growth.

a TRAMP-C2 cells were implanted subcutaneously in 6- to 8-week-old WT and *Ack1* KO male mice**.** The tumors weights are as shown (n=10 mice in each group). Data are represented as mean ± SEM from and p values were determined by Mann-Whitney test, two-tailed. **b** A picture of TRAMP-C2 tumors implanted subcutaneously in WT and *Ack1* KO mice. **c** A representative flow cytometry analysis and gating of $CD44^{hi}/CD62L^{low}$ on CD8 gated population isolated from splenocytes of WT and *Ack1* KO mice implanted with TRAMP-C2 tumors. **d** A representative flow cytometry analysis and gating of CD137 on CD8 gated population isolated from splenocytes of WT and *Ack1* KO mice implanted with TRAMP-C2 tumors. **e** A representative flow cytometry analysis of CD69 on CD4 gated population isolated from splenocytes of WT and *Ack1* KO mice implanted with TRAMP-C2 tumors. **f,** A representative flow cytometry analysis of CD4 and CD8 population in lymph nodes drained from WT and *Ack1* KO mice implanted with TRAMP-C2 tumors. Source data are provided as a Source Data file.

Supplementary Fig. 8

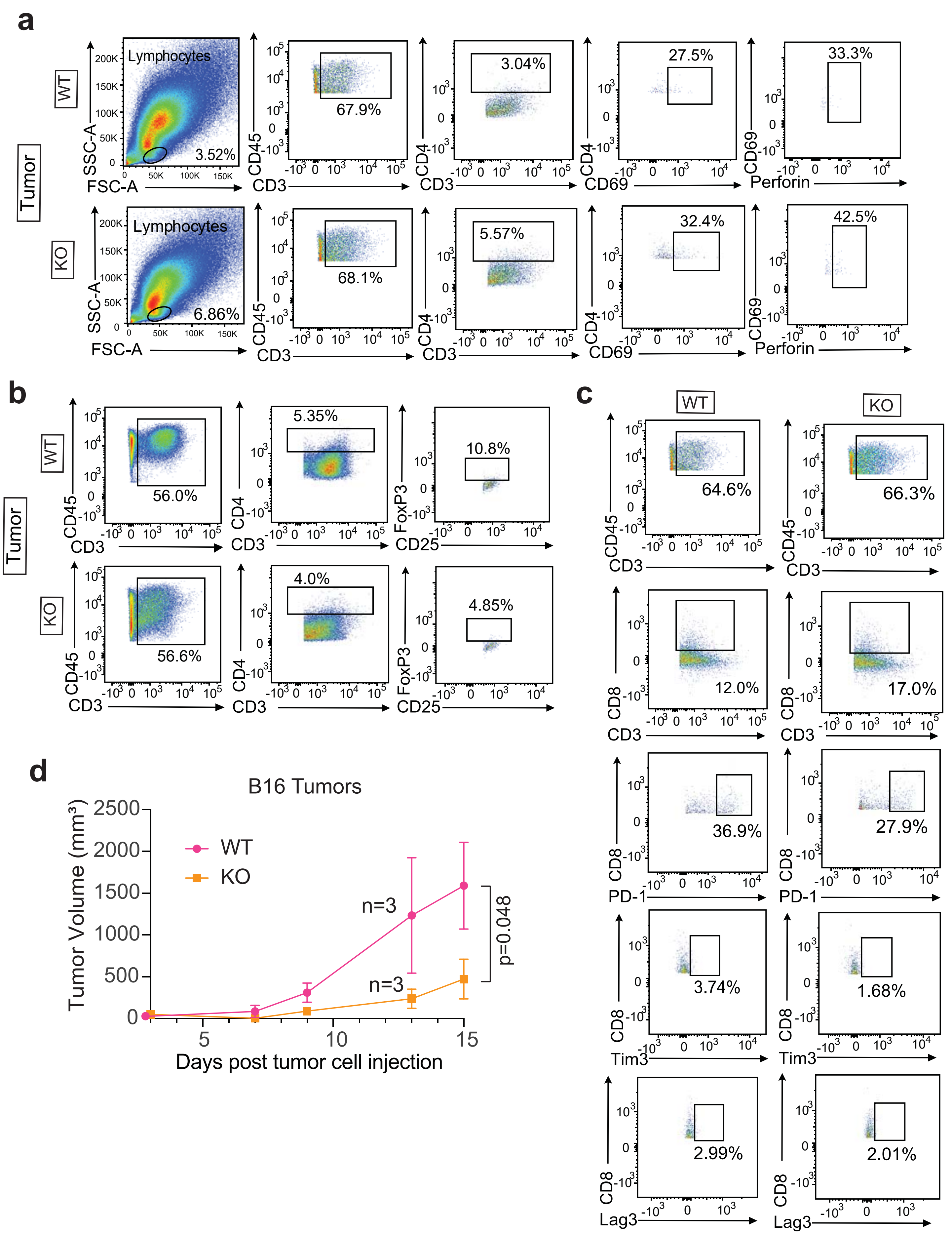

Supplementary Fig. 8: Alterations in the T cell sub-types upon loss of *Ack1* dampening tumor growth.

a A representative flow cytometry analysis and gating of $CD4^+$ $CD69^+$ T cells expressing perforin in the TILs of TRAMP-C2 tumors implanted in WT and *Ack1* KO mice. **b** A representative flow cytometry analysis and gating of Tregs in the TRAMP-C2 tumors implanted in WT and *Ack1* KO mice. **c** A representative flow cytometry analysis and gating of exhaustion markers on CD8 gated population in the TILs of TRAMP-C2 tumors implanted WT and *Ack1* KO mice. **d** Tumor volumes of subcutaneously induced B16 (Melanoma) cells in WT and *Ack1* KO mice (n=3 in each group). Data are represented as mean ± SEM and p values were determined by unpaired two-tailed Student's t-test. Source data are provided as a Source Data file.

Supplementary Fig. 9

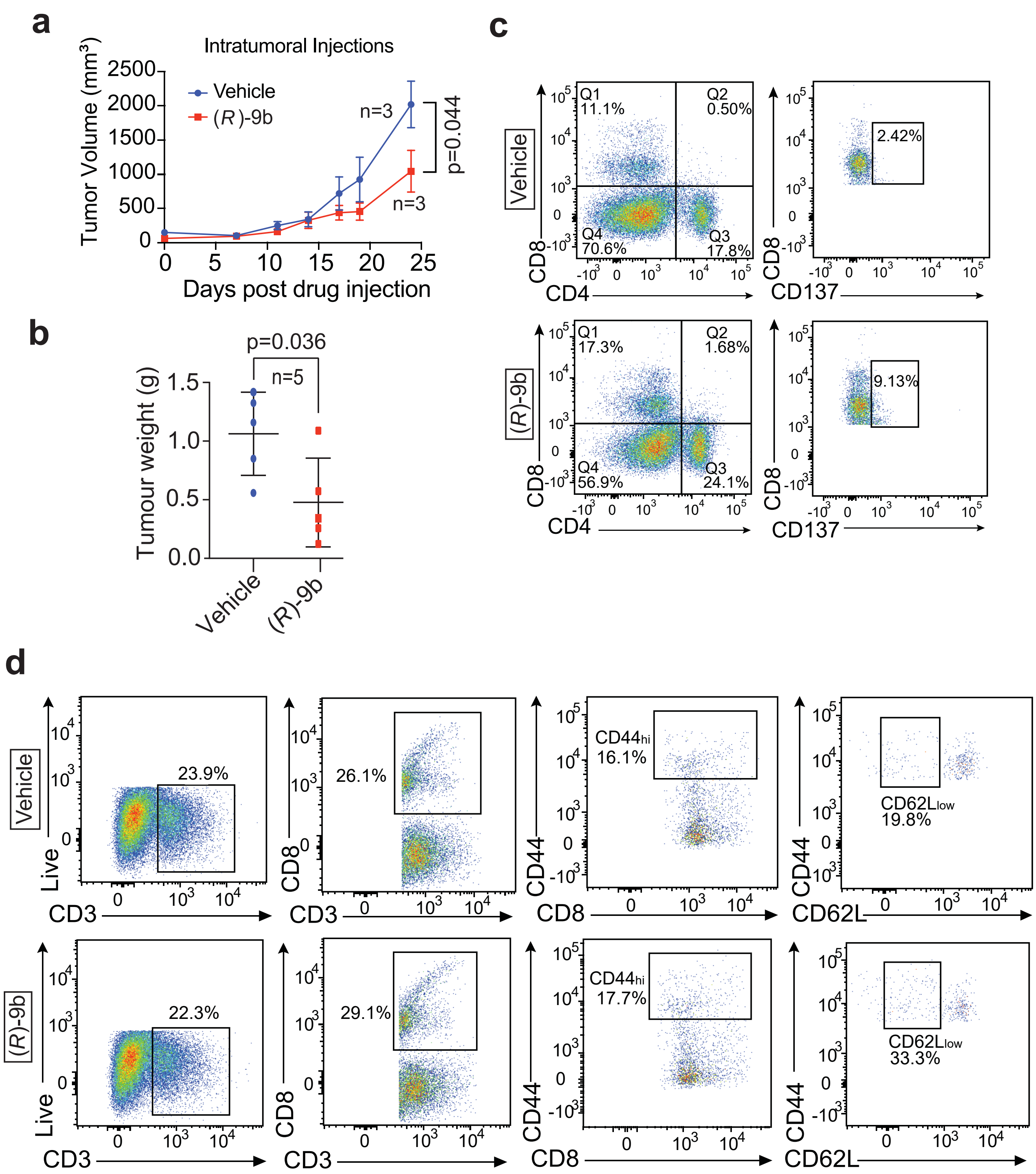

Supplementary Fig. 9: ACK1 inhibitor (*R*)-9b causes T cell activation in TRAMP-C2 tumor bearing immunocompetent mice.

a TRAMP-C2 implanted 6- to 8-week-old C57BL/6 male mice were injected either with vehicle (6% Captisol) or (*R*)-**9b** in 6% Captisol intratumorally for five times a week, for about 3.5 weeks. The tumor volumes were measured using calipers (n=3 in each group). **b** TRAMP-C2 implanted 6- to 8-week-old C57BL/6 male mice were injected either with vehicle (6% Captisol) or (*R*)-**9b** in 6% Captisol. The weights of the tumors are shown (n = 5 mice in each group). For **a** and **b**, the data are represented as mean ± SEM and p values were determined by unpaired two-tailed Student's t-test. **c** A representative flow cytometry analysis and gating of CD137 on CD8 gated population isolated from splenocytes of vehicle and (*R*)-**9b** injected mice implanted with TRAMP-C2 tumors. **d** A representative flow cytometry analysis and gating of $CD44^{hi}$ $CD62L^{low}$ on CD8 gated population isolated from splenocytes of vehicle and (*R*)-**9b** injected mice implanted with TRAMP-C2 tumors. Source data are provided as a Source Data file.

Supplementary Fig. 10

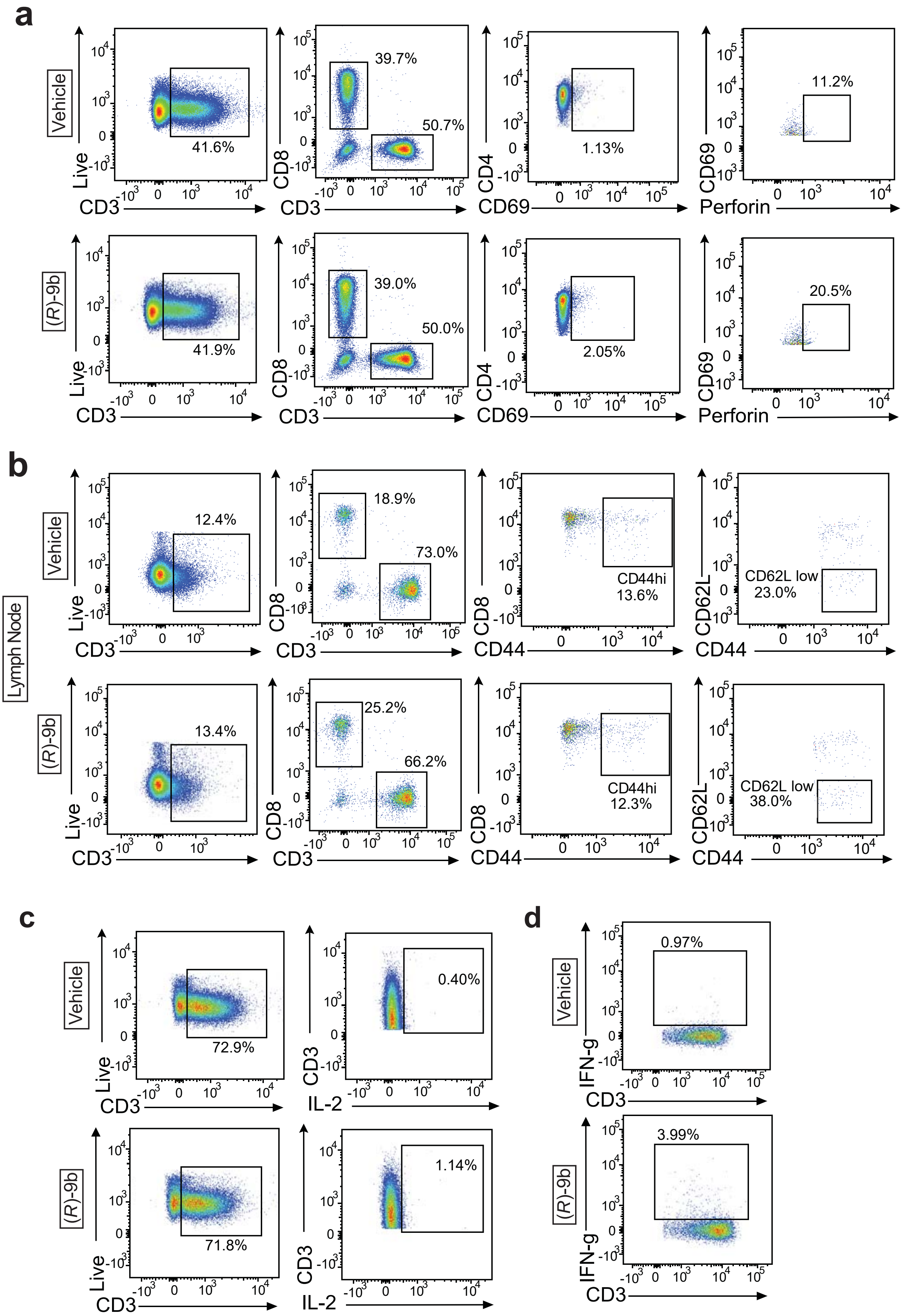

Supplementary Fig. 10: T cell activation upon pharmacological inhibition of ACK1.

a TRAMP-C2 implanted 6- to 8-week-old C57BL/6 male mice were injected either with vehicle (6% Captisol) or (*R*)-**9b** in 6% Captisol. Flow cytometry of splenocytes was done to assess $CD4^{+}$ $CD69^{+}$ T cells expressing perforin. **b** A representative flow cytometry analysis and gating of $CD44^{hi}$/$CD62L^{low}$ on CD8 gated population in the tumor draining lymph nodes. **c** and **d** A representative flow cytometry analysis of IL-2 (**c**) and IFN-g (**d**) gated on CD3 population. Source data are provided as a Source Data file.

Supplementary Fig. 11

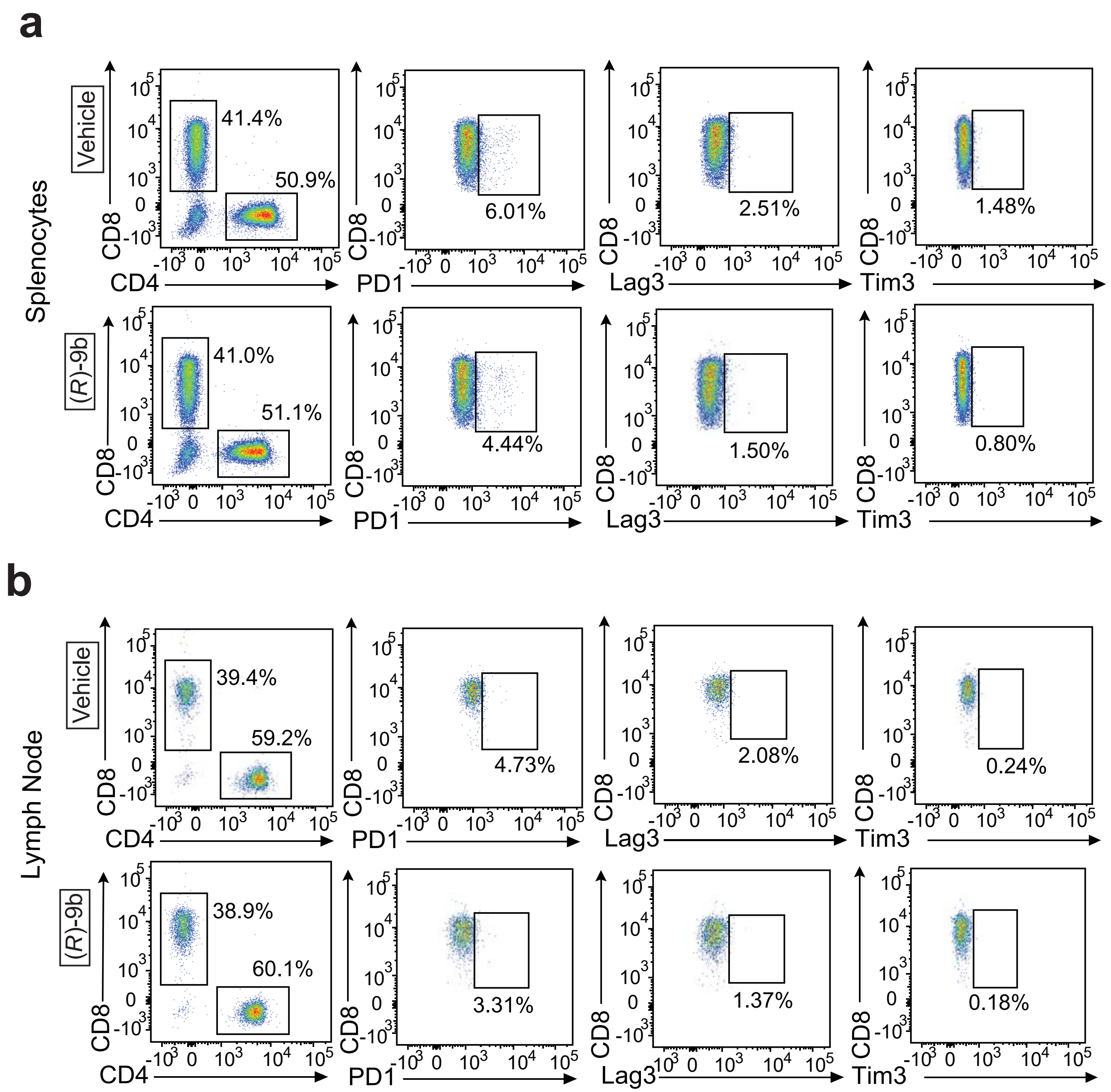

Supplementary Fig. 11: Overcoming T cell exhaustion upon pharmacological inhibition of ACK1. **a** and **b** TRAMP-C2 implanted 6- to 8-week-old C57BL/6 male mice were injected either with vehicle (6% Captisol) or (*R*)-**9b** in 6% Captisol. Flow cytometry of splenocytes (**a**) and drained lymph nodes (**b**) was performed to assess the levels of exhaustion markers, PD1, Lag3 and Tim3 gated on CD8 population (n = 3 mice in each group). Representative scatter plots are shown. Source data are provided as a Source Data file.

Supplementary Fig. 12

a

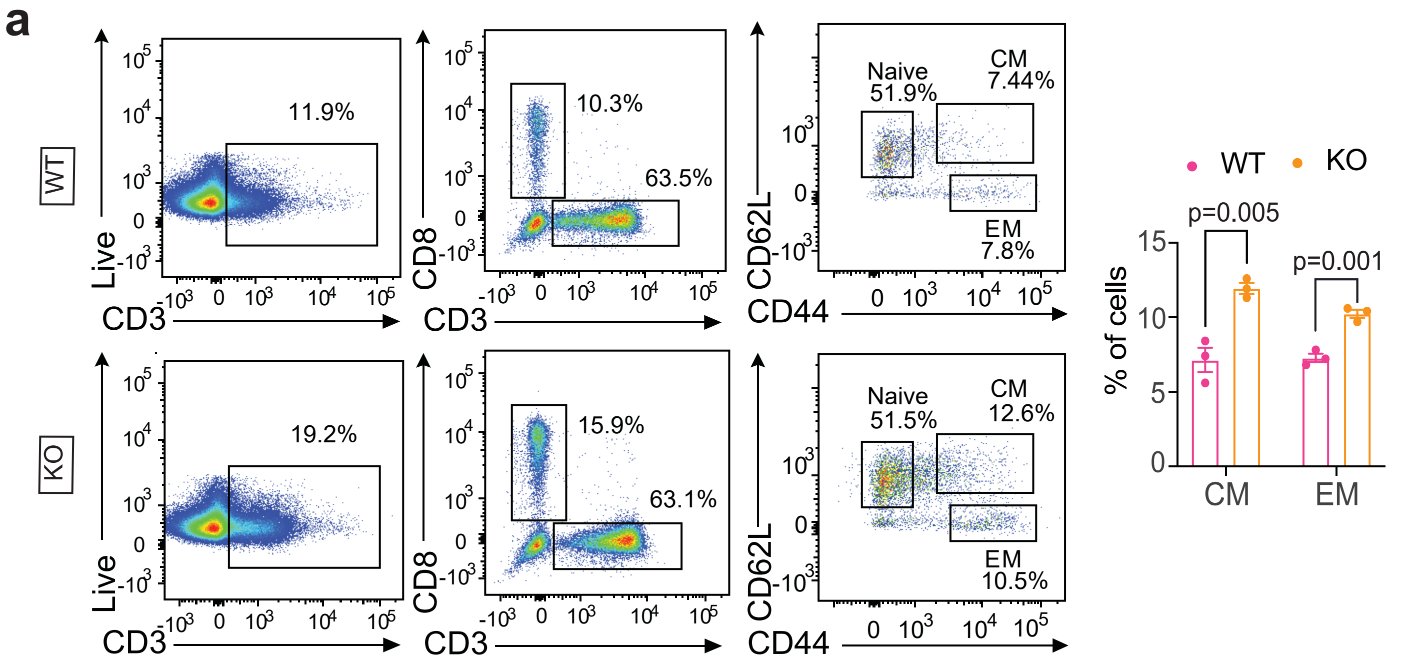

b

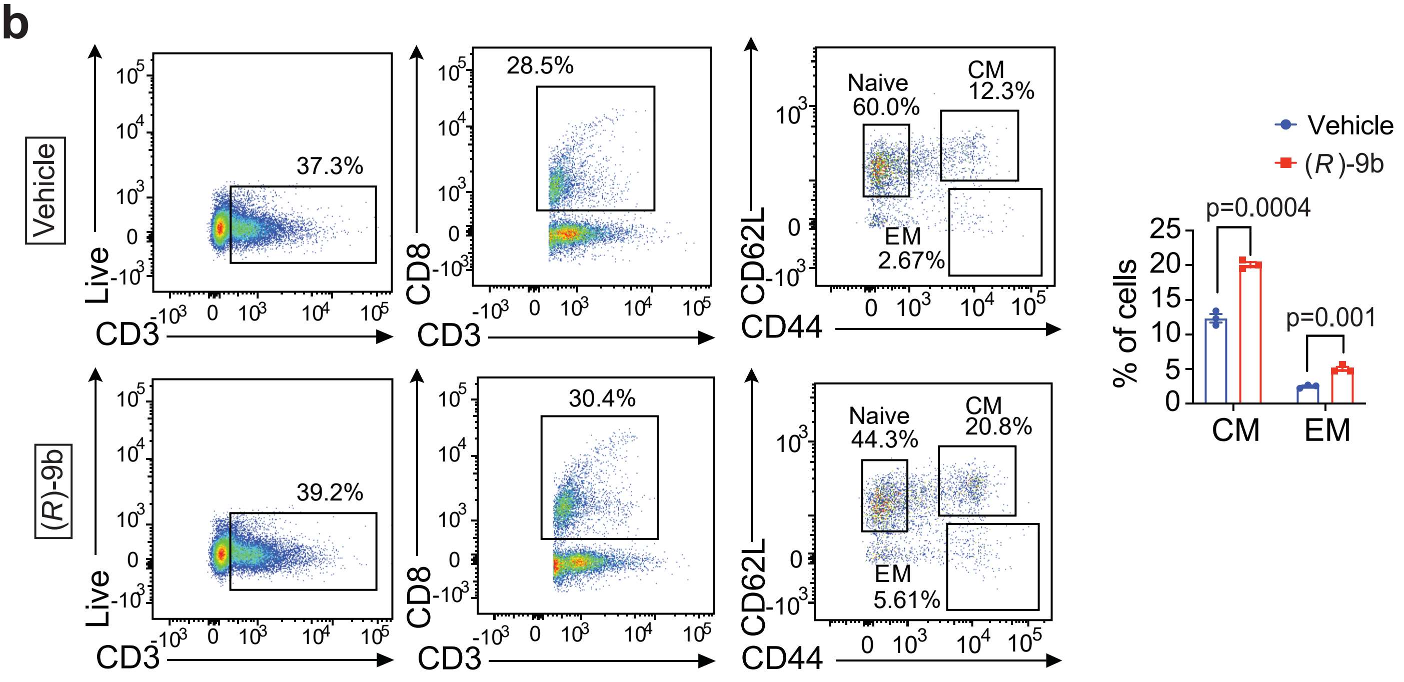

Supplementary Fig. 12: Increased effector memory T cell levels upon genetic and pharmacological inhibition of ACK1 activity.

a Flow cytometry analysis of naïve, central memory (CM) and effector memory (EM) $CD8^+$ T cell population in the splenocytes isolated from TRAMP-C2 tumor bearing WT and *Ack1* KO mice (n=3 mice in each group). **b** Flow cytometry analysis of naïve, central memory (CM) and effector memory (EM) $CD8^+$ T cell population in the splenocytes isolated from TRAMP-C2 tumor bearing C57BL/6 male mice injected either with vehicle (6% Captisol) or (*R*)-**9b** in 6% Captisol (n=3 mice in each group). Representative scatter plots are shown. For **a** and **b** (right panel), the data are represented as mean ± SEM from two independent experiments and p values were determined by unpaired two-tailed Student's t-test. Source data are provided as a Source Data file.

Supplementary Fig. 13

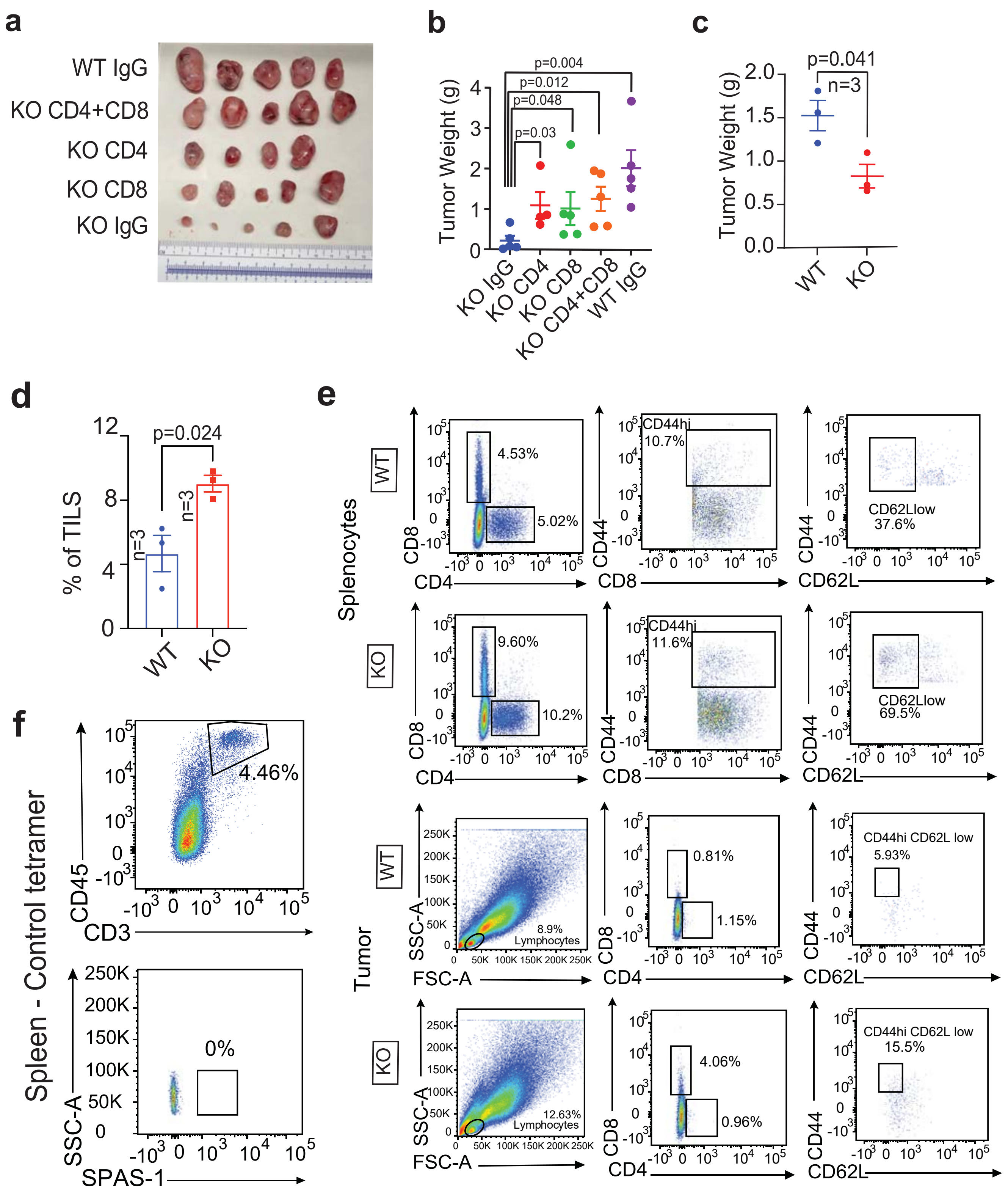

Supplementary Fig. 13: Effect of antibody depletion and adoptive transfer of T cells from *Ack1* KO and WT mice on TRAMP-C2 tumor growth.

a and **b** A picture of tumors (**a**) and weights of tumors (**b**) from WT and *Ack1* KO mice injected with CD4 or CD8 (or IgG as a control) depletion antibodies (n = 5 mice in each group expect KO CD4). For **b**, the data are represented as mean ± SEM from two independent experiments and p values were determined by unpaired two-tailed Student's t-test. **c** Adoptive transfer of T cells from WT and *Ack1* KO mice into NSG mice was done, followed by tumor weight measurements (n = 3 mice in each group). **d** A histogram of lymphocyte population isolated from TRAMP-C2 tumors implanted in NSG mice, which were earlier subjected to adoptive transfer of T cells from WT and *Ack1* KO mice, as described above (n=3 mice in each group). For **c** and **d**, data are represented as mean ± SEM from three independent experiments and p values were determined by unpaired two-tailed Student's t-test. **e** A representative flow cytometry analysis of $CD44^{hi}$ $CD62L^{low}$ on CD8 gated population in the splenocytes and TILs isolated from mice that had adoptively transferred T cells from WT and *Ack1* KO mice. **f** A representative scatter plot of the control tetramer stained splenocytes assessed by flow cytometry. Source data are provided as a Source Data file.

Supplementary Fig. 14

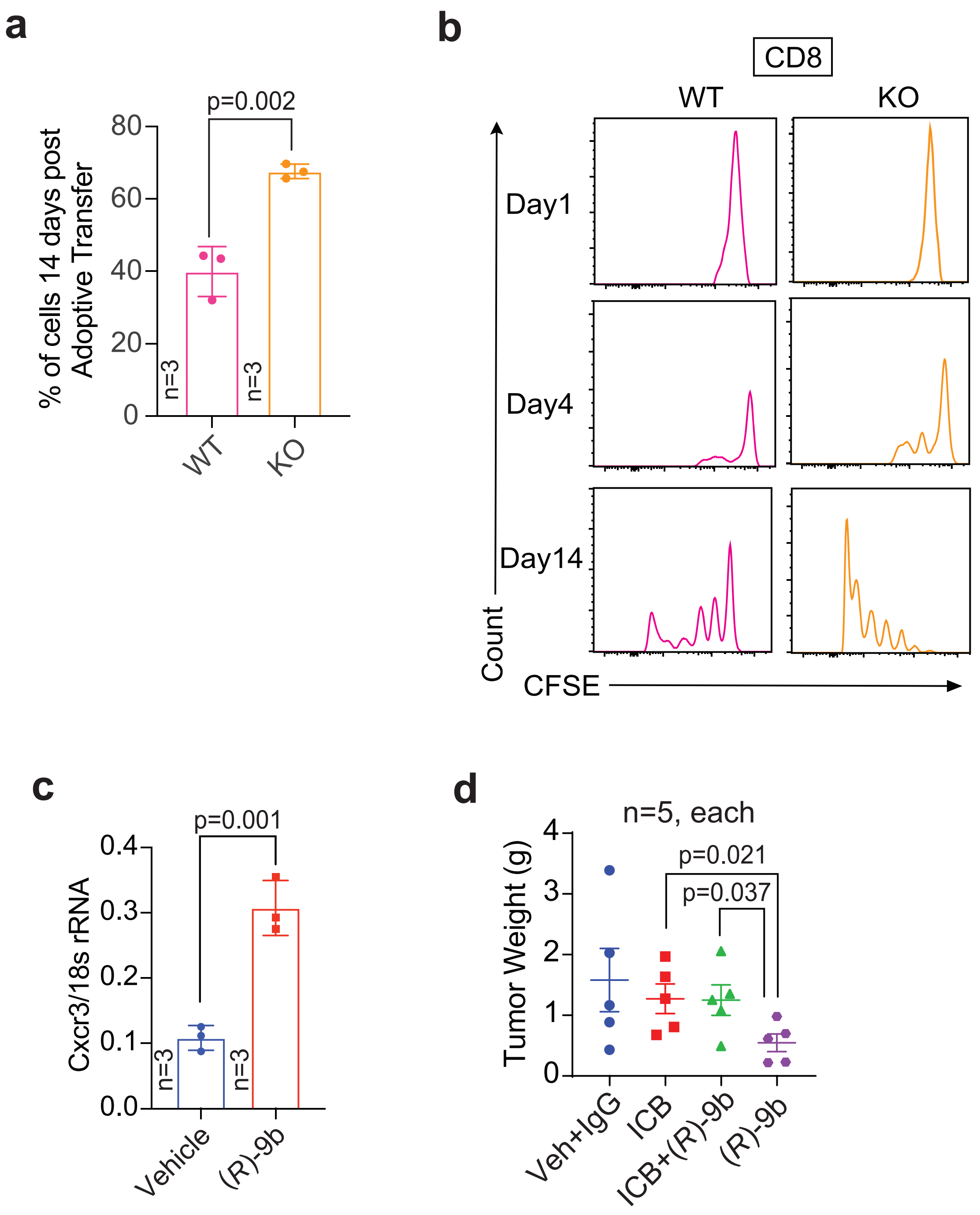

Supplementary Fig. 14: Persistence of T cells upon *Ack1* loss and effect of ICB & ACK1 inhibition on TRAMP-C2 tumor growth

a and **b** Flow cytometric analysis of persistence of adoptively transferred CFSE-stained T cells from WT and *Ack1* KO mice into NSG mice (n=3 mice in each group). For **a**, data are represented as mean ± SEM and p value was determined by unpaired two-tailed Student's t-test. **c** RNA was prepared from the TRAMP-C2 tumors formed in C57BL/6 male mice treated with vehicle or (*R*)-**9b,** followed by real time PCR of Cxcr3 (n=3 biologically independent samples, 3 replicates). The p value was determined by unpaired two-tailed Student's t-test. **d** TRAMP-C2 cells were implanted subcutaneously in 6- to 8-week-old C57BL/6 male mice and were injected with vehicle (6% Captisol) or (*R*)-**9b** or αPD-1 & αCTLA-4 antibodies (ICB) or its combination with (*R*)-**9b**. The weights of the tumors are shown (n=5 mice in each group). Data are represented as mean ± SEM and p values were determined by unpaired two-tailed Student's t-test. Source data are provided as a Source Data file.

Supplementary Fig. 15

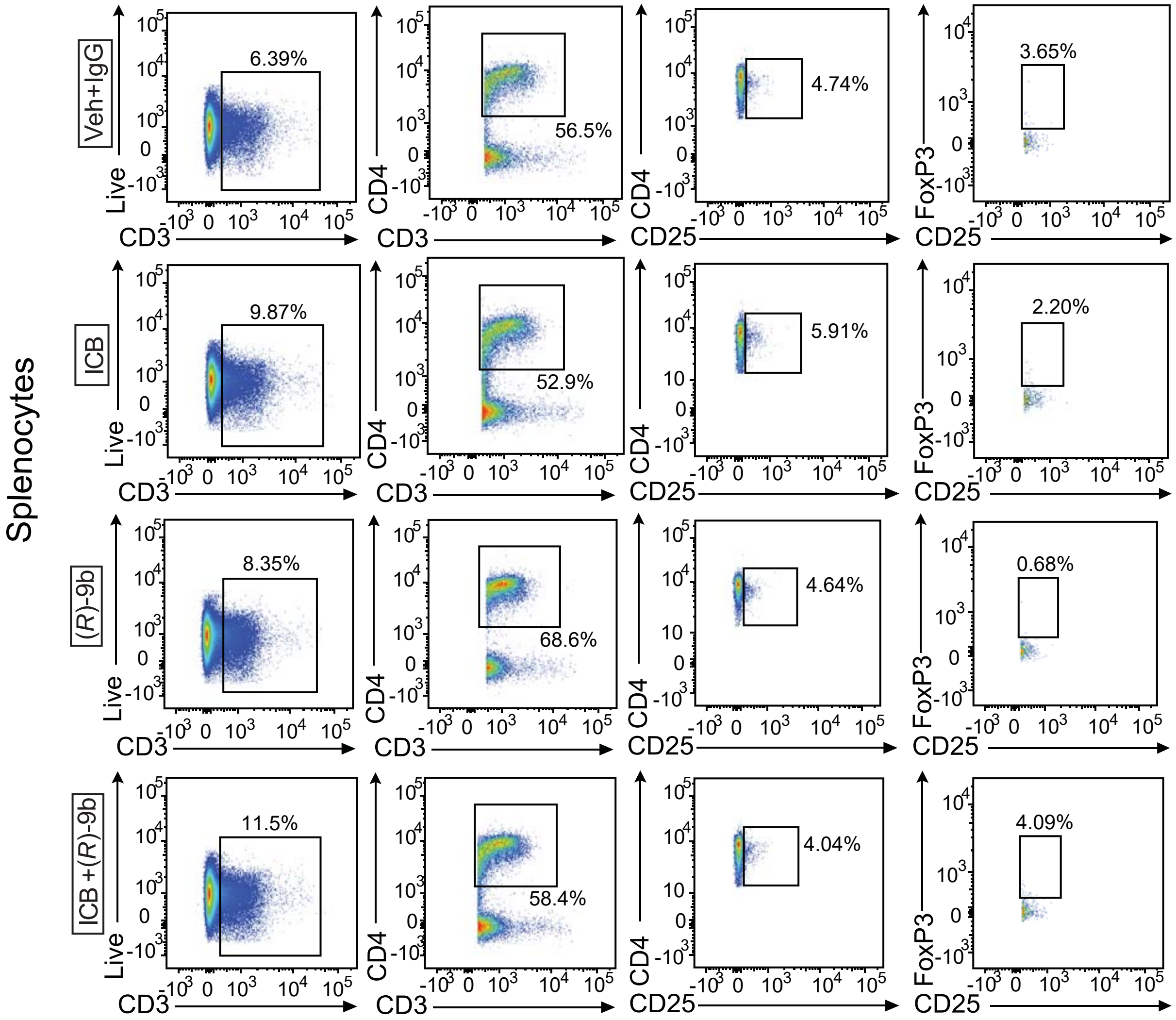

Supplementary Fig. 15. ICB hampers immune surveillance of prostate tumors by increasing Tregs.

A representative flow cytometry analysis of Treg population in the splenocytes isolated from TRAMP-C2 cells implanted mice treated with vehicle+IgG (6% Captisol), (*R*)-**9b**, αPD-1 & αCTLA-4 antibodies (ICB), or its combination with (*R*)-**9b**. Source data are provided as a Source Data file.

Supplementary Fig. 16

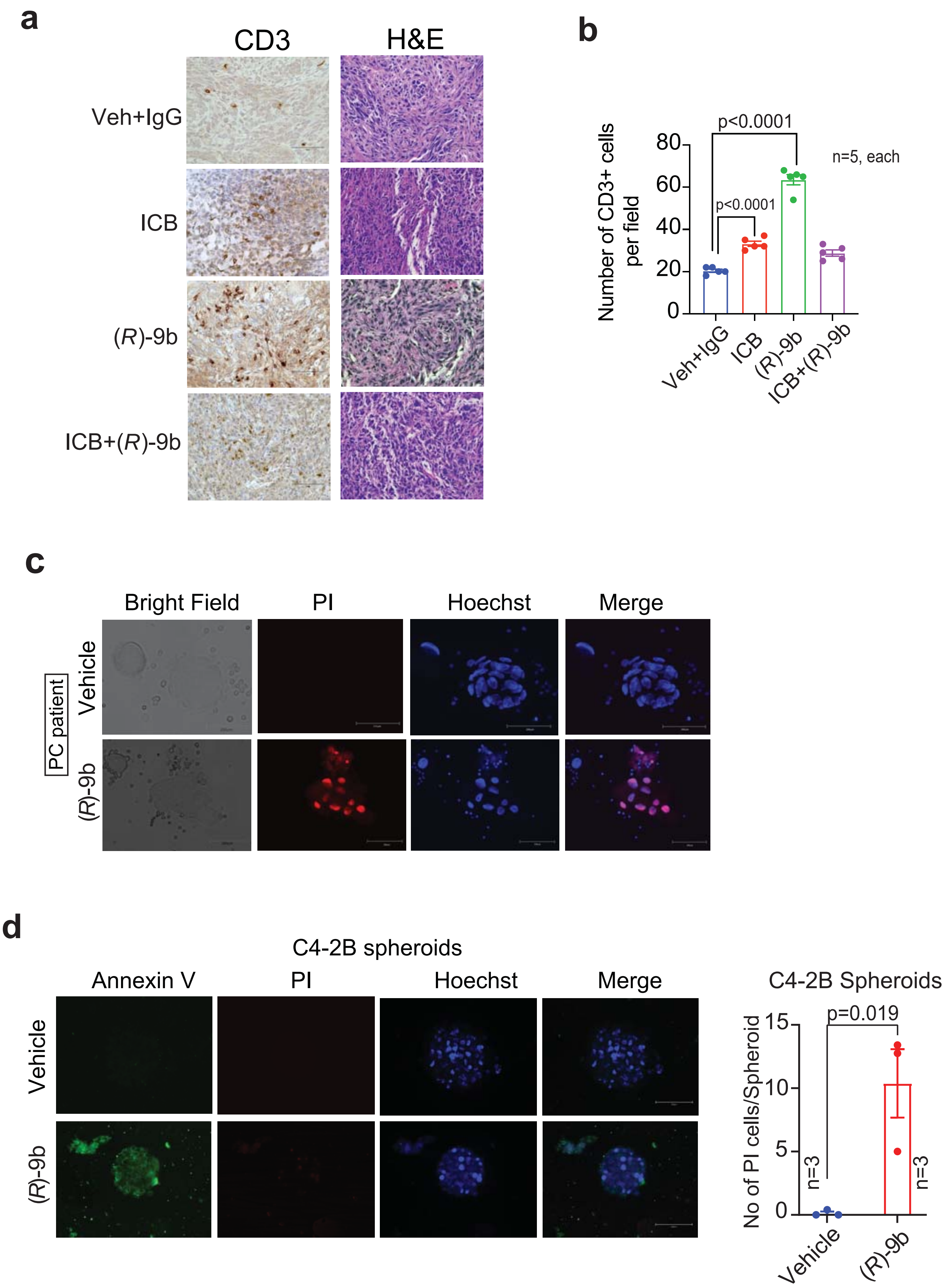

Supplementary Fig. 16: ACK inhibition improves immune infiltration in prostate tumors

a Immunohistochemistry of anti-CD3 antibody in formalin fixed paraffin sections of TRAMP-C2 tumors from mice treated with Vehicle+IgG, (*R*)-**9b**, αPD-1 & αCTLA-4 antibodies (ICB), or its combination with (*R*)-**9b.** Scale bar 75μM. **b** Corresponding quantification of CD3+ cells (n=5 mice in each group). Data are represented as mean ± SEM and p value was determined by unpaired two-tailed Student's t-test. **c** PBMCs from prostate cancer patient were treated with vehicle and (*R*)-**9b** for 6 hours, washed and co-cultured with C4-2B spheroids. After 24h, the spheroids were stained with PI and Hoechst 33258 and observed under fluorescent microscope. Representative images are as shown (n = 3 biologically independent experiments). **d** C4-2B spheroids were incubated with vehicle or (*R*)-**9b**. After 24h, the spheroids were stained with PI and Hoechst 33258 and observed under fluorescent microscope. Representative images are as shown (n = 3 biologically independent experiments). Data are represented as mean ± SEM from three biologically independent experiments and p value was determined by unpaired two-tailed Student's t-test. Source data are provided as a Source Data file.

Supplementary Fig. 17

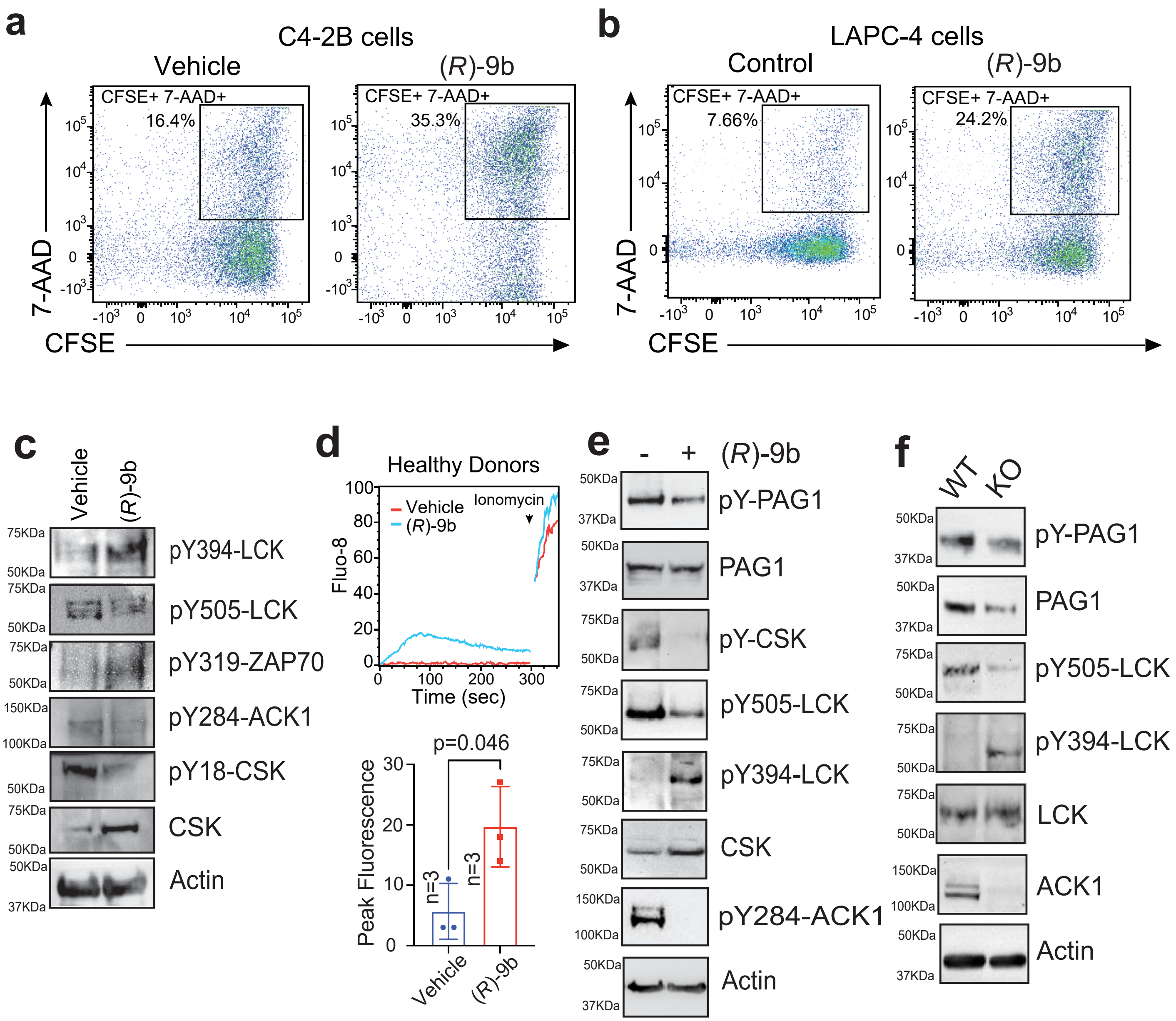

Supplementary Fig. 17: Effective cancer cell killing and decrease in PAG Tyr-phosphorylation upon loss of ACK1 kinase activity.

a and **b** PBMC were isolated from healthy donors and treated with vehicle and (*R*)-**9b** for 6h, and co-cultured with CFSE stained C4-2B (**a**), and LAPC-4 (**b**) cells. Tumor cell lysis was assessed by flow cytometry using 7-AAD staining. Representative scatter plots are as shown. Data are representative of three independent experiments. **c** and **d** PBMCs were isolated from blood of healthy donors. The PBMCs were treated with (*R*)-**9b** for 6 hours. Calcium flux was analyzed by flow cytometry (n=3 biologically independent samples) (**d**) and immunoblotting (**c**) was performed with indicated antibodies. **e** Lysates were made from Jurkat cells treated with (*R*)-**9b** for 6h. Lysates were immunoprecipitated with PAG antibody, followed by immunoblotting with pTyr antibodies (top panel). Lower blots were immunoblotted with indicated antibodies. **f** Lysates were made from spleen of WT and KO mice. The lysates were immunoprecipitated with PAG antibody, followed by immunoblotting with pTyr antibodies (top panel). Lower blots were immunoblotted with indicated antibodies. For **c**, **e** and **f**, representative images are shown (n = 3 biologically independent experiments). For **d**, the data are represented as mean ± SEM and p value was determined by unpaired two-tailed Student's t-test. Source data are provided as a Source Data file.

Supplementary Table 1. Data collection and refinement statistics.

	ACK1*(*R*)-9b
Wavelength	
Resolution range	41.56 - 1.79 (1.854 - 1.79)
Space group	P 1 21 1
Unit cell	71.016 42.819 92.623 Å<br>90º 99.652º 90º
Total reflections	169864 (18601)
Unique reflections	49624 (5101)
Multiplicity	3.4 (3.6)
Completeness (%)	94.74 (97.83)
Mean I/sigma(I)	11.93 (3.02)
Wilson B-factor	19.60
R-merge	0.0581 (0.4335)
R-meas	0.06915 (0.5111)
R-pim	0.0371 (0.2683)
CC1/2	0.997 (0.876)
CC*	0.999 (0.966)
Reflections used in refinement	49520 (5101)
Reflections used for R-free	2515 (243)
R-work	0.1784 (0.2290)
R-free	0.2031 (0.2426)
CC(work)	0.953 (0.747)
CC(free)	0.947 (0.685)
Number of non-hydrogen atoms	4626
macromolecules	4289
ligands	58
solvent	279
Protein residues	534
RMS(bonds)	0.004
RMS(angles)	0.77
Ramachandran favored (%)	98.09
Ramachandran allowed (%)	1.91
Ramachandran outliers (%)	0.00
Rotamer outliers (%)	0.00
Clashscore	0.70
Average B-factor (all atoms)	28.76
macromolecules	28.82
ligands	29.91
solvent	27.54
Number of TLS groups	13

Statistics for the highest-resolution shell are shown in parentheses.

Supplementary Table 2. List of primers

**qRT-PCR Primers:**	
Actin FP: CATTGCTGACAGGATGCAGAAGG	This paper
Actin RP: TGCTGGAAGGTGGACAGTGAGG	This paper
ACK1 FP: CACACAGGACATGGTGACAGTG	This paper
ACK1 RP: CAGTTCCACACTCAGCAGGTCA	This paper
Il-2 FP: GCGGCATGTTCTGGATTTGACTC	This paper
Il-2 RP: CCACCACAGTTGCTGACTCATC	This paper
Ifn-g FP: CAGCAACAGCAAGGCGAAAAAGG	This paper
Ifn-g RP: TTTCCGCTTCCTGAGGCTGGAT	This paper
Prf FP: ACACAGTAGAGTGTCGCATGTAC	This paper
Prf RP: GTGGAGCTGTTAAAGTTGCGGG	This paper
Cxcr3 FP: TACGATCAGCGCCTCAATGCCA	This paper
Cxcr3 RP: AGCAGGAAACCAGCCACTAGCT	This paper
**ChIP PCR Primer**	
Human CXCL10 FP: GAACAGTGATTTACCTGGACA	
Human CXCL10 RP: CTTGCCAGTTCCAGATCTTTG	
Mouse Cxcl10 FP: TGTCACCTCTATGCGAGATCTATG	
Mouse Cxcl10 RP: AAGAGGAATTGCAAAGCTAATTGT	
