# Supplementary material for: Inhibiting ACK1-mediated phosphorylation of C-terminal Src kinase counteracts prostate cancer immune checkpoint blockade resistance
Source: Nat Commun. 2022 Nov 14;13:6929. doi: 10.1038/s41467-022-34724-5 (PMC9663509; doi:10.1038/s41467-022-34724-5)
Supplement: Supplementary file 3 — Reporting Summary [file 41467_2022_34724_MOESM3_ESM.pdf]

nature portfolio

Corresponding author(s): Nupam Mahajan

Last updated by author(s): 2022/10/19

Reporting Summary

Nature Portfolio wishes to improve the reproducibility of the work that we publish. This form provides structure for consistency and transparency in reporting. For further information on Nature Portfolio policies, see our Editorial Policies and the Editorial Policy Checklist.

Statistics

For all statistical analyses, confirm that the following items are present in the figure legend, table legend, main text, or Methods section.

n/a	Confirmed	
☐	☒	The exact sample size (*n*) for each experimental group/condition, given as a discrete number and unit of measurement
☐	☒	A statement on whether measurements were taken from distinct samples or whether the same sample was measured repeatedly
☐	☒	The statistical test(s) used AND whether they are one- or two-sided<br>*Only common tests should be described solely by name; describe more complex techniques in the Methods section.*
☐	☒	A description of all covariates tested
☐	☒	A description of any assumptions or corrections, such as tests of normality and adjustment for multiple comparisons
☐	☒	A full description of the statistical parameters including central tendency (e.g. means) or other basic estimates (e.g. regression coefficient) AND variation (e.g. standard deviation) or associated estimates of uncertainty (e.g. confidence intervals)
☐	☒	For null hypothesis testing, the test statistic (e.g. *F*, *t*, *r*) with confidence intervals, effect sizes, degrees of freedom and *P* value noted<br>*Give P values as exact values whenever suitable.*
☒	☐	For Bayesian analysis, information on the choice of priors and Markov chain Monte Carlo settings
☒	☐	For hierarchical and complex designs, identification of the appropriate level for tests and full reporting of outcomes
☒	☐	Estimates of effect sizes (e.g. Cohen's *d*, Pearson's *r*), indicating how they were calculated

Our web collection on statistics for biologists contains articles on many of the points above.

Software and code

Policy information about availability of computer code

Data collection	Western Blotting - Invitrogen™ iBright™ FL1000 imaging system<br>qRT-PCR - StepOne and StepOnePlus Software v2.3 Applied Biosystems<br>Microscopy - EVOS M5000 Imaging System Software Invitrogen<br>Flow cytometry – BD CantoII and LSRII (BD Biosciences)<br>LC-MS/MS - Capillary liquid chromatography interfaced to a mass spectrometer (nano-LC-MS/MS).
Data analysis	Prism8: GraphPad https://www.graphpad.com/scientific-software/prism/<br>FlowJo V10 (BD Biosciences) https://www.bdbiosciences.com/en-us/products/software/flowjo-v10-software<br>VOS M5000 Imaging System Software Invitrogen: https://www.thermofisher.com/us/en/home/technical-resources/software-downloads/evos-m5000-imaging-system-software-download.html<br>ImageJ National Institutes of Health: https://imagej.nih.gov/ij/<br>Adobe Photoshop Adobe: https://www.adobe.com/products/photoshop.html<br>Adobe Illustrator Adobe: https://www.adobe.com/products/illustrator.html<br>RNA-seq Analysis Illumina's bcl2fastq software https://support.illumina.com/sequencing/sequencing_software/bcl2fastq-conversion-software/documentation.html<br>Ensembl release 101 primary assembly with STAR version 2.7.9a1

For manuscripts utilizing custom algorithms or software that are central to the research but not yet described in published literature, software must be made available to editors and reviewers. We strongly encourage code deposition in a community repository (e.g. GitHub). See the Nature Portfolio guidelines for submitting code & software for further information.

Data

Policy information about availability of data

All manuscripts must include a data availability statement. This statement should provide the following information, where applicable:

- Accession codes, unique identifiers, or web links for publicly available datasets
- A description of any restrictions on data availability
- For clinical datasets or third party data, please ensure that the statement adheres to our policy

RNA-sequencing datasets used in this study can be found at: https://www.ncbi.nlm.nih.gov/geo/query/acc.cgi GEO accession GSE211835.
The structure ACK1 kinase domain with the compound (R)-9b can be found at: https://www.rcsb.org/structure/7KP6 PDB ID 7KP6 (Deposition ID D_1000252889).
Proteomics data has been submitted in PRIDE: https://www.ebi.ac.uk/pride/archive/projects/PXD037546/
Project accession: PXD037546; Project DOI: 10.6019/PXD037546
Username: reviewer_pxd037546@ebi.ac.uk
Password: xIZA6hxV
The database will become public once the manuscript is accepted

All the relevant information on the key findings and validation are there in the article, supplementary figures and tables. The raw data related to the manuscript are included in the "Source Data" files.
Any additional datasets generated during and/or analyzed during the current study are available from the corresponding author on reasonable request.

Human research participants

Policy information about studies involving human research participants and Sex and Gender in Research.

Reporting on sex and gender	Tissue and blood samples from male patients with prostate cancer (or healthy male individual) were used.
Population characteristics	Not applicable
Recruitment	This study is primarily direced towards prostate cancer, hence only male patients or healthy male individual were included.
Ethics oversight	Patients were consented to our IRB-approved genitourinary banking protocol [Human Research Protection Office (HRPO) no.: 201411135] to allow tissue collection.

Note that full information on the approval of the study protocol must also be provided in the manuscript.

Field-specific reporting

Please select the one below that is the best fit for your research. If you are not sure, read the appropriate sections before making your selection.

☒ Life sciences ☐ Behavioural & social sciences ☐ Ecological, evolutionary & environmental sciences

For a reference copy of the document with all sections, see nature.com/documents/nr-reporting-summary-flat.pdf

Life sciences study design

All studies must disclose on these points even when the disclosure is negative.

Sample size	The sample size was chosen on the basis of prior studies that showed significant effects with similar sample sizes. All mice experiments had 3-10 mice in each arm. Reference: PMID: 28609657
Data exclusions	No data was excluded from the analysis.
Replication	Each experiment was repeated atleast twice, thrice in most cases for reproducibility. All primers were tested by gel electrophoresis and melt curves evaluated before using them in the quantitative assays for reproducibility.<br>Western blots were repeated atleast twice thrice in most cases to ensure reproducibility and captured digitally using iBright imager. All data are available for review.<br>Replicated experiments were successful and support conclusions drawn in this report.
Randomization	Mice were randomly assigned to two or more groups prior to the injection of cells or drug. For all other experiments, allocations were random in this study.
Blinding	Investigators were not blinded for any of the experiments, including to the animal assignments for tumor formation studies and molecular analysis. However, standard laboratory procedures of randomization was followed.<br>Each experiment was designed with appropriate controls and samples for comparison were collected and analyzed under the same conditions. All tissue sections were independently evaluated by pathologist for presence of antibody validation studies and for tumor versus

normal determination.

Behavioural & social sciences study design

All studies must disclose on these points even when the disclosure is negative.

Study description	*Briefly describe the study type including whether data are quantitative, qualitative, or mixed-methods (e.g. qualitative cross-sectional, quantitative experimental, mixed-methods case study).*
Research sample	*State the research sample (e.g. Harvard university undergraduates, villagers in rural India) and provide relevant demographic information (e.g. age, sex) and indicate whether the sample is representative. Provide a rationale for the study sample chosen. For studies involving existing datasets, please describe the dataset and source.*
Sampling strategy	*Describe the sampling procedure (e.g. random, snowball, stratified, convenience). Describe the statistical methods that were used to predetermine sample size OR if no sample-size calculation was performed, describe how sample sizes were chosen and provide a rationale for why these sample sizes are sufficient. For qualitative data, please indicate whether data saturation was considered, and what criteria were used to decide that no further sampling was needed.*
Data collection	*Provide details about the data collection procedure, including the instruments or devices used to record the data (e.g. pen and paper, computer, eye tracker, video or audio equipment) whether anyone was present besides the participant(s) and the researcher, and whether the researcher was blind to experimental condition and/or the study hypothesis during data collection.*
Timing	*Indicate the start and stop dates of data collection. If there is a gap between collection periods, state the dates for each sample cohort.*
Data exclusions	*If no data were excluded from the analyses, state so OR if data were excluded, provide the exact number of exclusions and the rationale behind them, indicating whether exclusion criteria were pre-established.*
Non-participation	*State how many participants dropped out/declined participation and the reason(s) given OR provide response rate OR state that no participants dropped out/declined participation.*
Randomization	*If participants were not allocated into experimental groups, state so OR describe how participants were allocated to groups, and if allocation was not random, describe how covariates were controlled.*

Ecological, evolutionary & environmental sciences study design

All studies must disclose on these points even when the disclosure is negative.

Study description	*Briefly describe the study. For quantitative data include treatment factors and interactions, design structure (e.g. factorial, nested, hierarchical), nature and number of experimental units and replicates.*
Research sample	*Describe the research sample (e.g. a group of tagged Passer domesticus, all Stenocereus thurberi within Organ Pipe Cactus National Monument), and provide a rationale for the sample choice. When relevant, describe the organism taxa, source, sex, age range and any manipulations. State what population the sample is meant to represent when applicable. For studies involving existing datasets, describe the data and its source.*
Sampling strategy	*Note the sampling procedure. Describe the statistical methods that were used to predetermine sample size OR if no sample-size calculation was performed, describe how sample sizes were chosen and provide a rationale for why these sample sizes are sufficient.*
Data collection	*Describe the data collection procedure, including who recorded the data and how.*
Timing and spatial scale	*Indicate the start and stop dates of data collection, noting the frequency and periodicity of sampling and providing a rationale for these choices. If there is a gap between collection periods, state the dates for each sample cohort. Specify the spatial scale from which the data are taken*
Data exclusions	*If no data were excluded from the analyses, state so OR if data were excluded, describe the exclusions and the rationale behind them, indicating whether exclusion criteria were pre-established.*
Reproducibility	*Describe the measures taken to verify the reproducibility of experimental findings. For each experiment, note whether any attempts to repeat the experiment failed OR state that all attempts to repeat the experiment were successful.*
Randomization	*Describe how samples/organisms/participants were allocated into groups. If allocation was not random, describe how covariates were controlled. If this is not relevant to your study, explain why.*
Blinding	*Describe the extent of blinding used during data acquisition and analysis. If blinding was not possible, describe why OR explain why blinding was not relevant to your study.*

Did the study involve field work? ☐ Yes ☒ No

Field work, collection and transport

Field conditions	*Describe the study conditions for field work, providing relevant parameters (e.g. temperature, rainfall).*
Location	*State the location of the sampling or experiment, providing relevant parameters (e.g. latitude and longitude, elevation, water depth).*
Access & import/export	*Describe the efforts you have made to access habitats and to collect and import/export your samples in a responsible manner and in compliance with local, national and international laws, noting any permits that were obtained (give the name of the issuing authority, the date of issue, and any identifying information).*
Disturbance	*Describe any disturbance caused by the study and how it was minimized.*

Reporting for specific materials, systems and methods

We require information from authors about some types of materials, experimental systems and methods used in many studies. Here, indicate whether each material, system or method listed is relevant to your study. If you are not sure if a list item applies to your research, read the appropriate section before selecting a response.

Materials & experimental systems

n/a	Involved in the study	
☐	☒	Antibodies
☐	☒	Eukaryotic cell lines
☒	☐	Palaeontology and archaeology
☐	☒	Animals and other organisms
☒	☐	Clinical data
☒	☐	Dual use research of concern

Methods

n/a	Involved in the study	
☒	☐	ChIP-seq
☐	☒	Flow cytometry
☒	☐	MRI-based neuroimaging

Antibodies

Antibodies used

LIVE/DEAD™ Fixable Aqua Dead Cell Stain Kit Invitrogen Cat#L34957
PE/Cyanine7 anti-mouse CD3ε Antibody Biolegend Cat#100320
APC Rat Anti-Mouse CD8a BD Pharmingen Cat#553035
Pacific Blue Rat Anti-Mouse CD4 BD Pharmingen Cat#558107
PerCP-Cy 5.5 Rat Anti-CD11b BD Pharmingen Cat#550993
PerCP-Cy™5.5 Rat Anti-Mouse CD19 BD Biosciences Cat#561113
PE anti-mouse NK-1.1 Antibody Biolegend Cat#108708
FITC Rat Anti-Mouse CD137 BD Pharmingen Cat#558975
FITC anti-mouse CD279 (PD-1) antibody Biolegend Cat#135214
PE anti-mouse CD44 Biolegend Cat# 103002
Percpcy5.5 anti-mouse CD62L Biolegend Cat# 104432
PE anti-mouse CD69 Biolegend Cat# 104508
APC/Cy7 anti-mouse CD45 Biolegend Cat#103116
Anti-mouse CD8β (Lyt 3.2) BioXCell Cat# BE0223
Anti-mouse CD4 BioXCell Cat# BE0003-1
H-2D(b) STHVNHLHC PE-Labeled Tetramer (SPAS-1) Synthesized from NIH Tetramer Core Facility
H-2K(b) SIINFEKL Brilliant Violet 421-Labeled control Tetramer synthesized from NIH Tetramer Core Facility
Alexa Fluor® 488 Anti-rabbit secondary antibody Invitrogen Cat#A11070
Anti-CSK Santa Cruz Bio Cat#sc-166560
Anti-FLAG Sigma Cat#F3165
Anti-HA Santa Cruz Bio Cat#sc-7392
Anti-MYC Cell Signaling Tech Cat#2276S
Anti-pTyr Santa Cruz Bio Cat#sc-508
Anti-ACK1 Santa Cruz Bio Cat#sc-28336
Anti-PD-1 Novus Biologicals NBP1-77276
Anti-LCKpY394 Invitrogen Cat#PA5-37628
Anti-LCKpY505 Cell Signaling Tech Cat#2751S
Anti-LATpY132 Invitrogen Cat#44-224
Anti-pZap70 Santa Cruz Bio Cat#sc-136248
Anti-PLCgpY783 Cell Signaling Tech Cat#2821P
Anti-cbp/PAG1 Santa Cruz Bio Cat#sc-365387
Anti-ACK1pY284 Millipore Cat#09-142

	Anti-LCK Santa Cruz Bio Cat#sc-166627<br>Anti-Ub Santa Cruz Bio Cat#sc-8017<br>Anti-Actin Sigma Cat#A2228<br>Anti-PerforinFITC Biolegend Cat#154310<br>Anti-FOXP3PE Biolegend Cat#320008
Validation	The effectiveness of the antibodies was confirmed by performing flow cytometry or immunoblotting experiments using the manufacturer's data associated with antibody, and their authentication data.<br>Fixable Aqua Dead Cell Stain (https://www.thermofisher.com/order/catalog/product/L34957)<br>anti-mouse CD3ε (https://www.biolegend.com/en-us/products/pe-cyanine7-anti-mouse-cd3epsilon-antibody-1899)<br>Anti-Mouse CD8a (https://www.bdbiosciences.com/en-us/products/reagents/flow-cytometry-reagents/research-reagents/single-color-antibodies-ruo/apc-rat-anti-mouse-cd8a.553035)<br>Anti-Mouse CD4 (https://www.bdbiosciences.com/en-us/products/reagents/flow-cytometry-reagents/research-reagents/single-color-antibodies-ruo/pacific-blue-rat-anti-mouse-cd4.558107)<br>Anti-Mouse CD11b (https://www.bdbiosciences.com/en-us/products/reagents/flow-cytometry-reagents/research-reagents/single-color-antibodies-ruo/percp-cy-5-5-rat-anti-cd11b.550993)<br>Anti-Mouse CD19 (https://www.bdbiosciences.com/en-us/products/reagents/flow-cytometry-reagents/research-reagents/single-color-antibodies-ruo/percp-cy-5-5-rat-anti-mouse-cd19.561113?gclid=EAIaIQobChMI0pSKw7TH-gIVR25vBB1aewljEAAYASAAEgJXhvD_BwE)<br>Anti-mouse NK-1.1 (https://www.bdbiosciences.com/en-us/products/reagents/flow-cytometry-reagents/research-reagents/single-color-antibodies-ruo/pe-mouse-anti-mouse-nk-1-1.561046)<br>Anti-mouse CD137 (https://www.bdbiosciences.com/en-us/products/reagents/flow-cytometry-reagents/research-reagents/single-color-antibodies-ruo/fitc-rat-anti-mouse-cd137.558975)<br>Anti-Mouse PD-1 (https://www.biolegend.com/en-us/products/fitc-anti-mouse-cd279-pd-1-antibody-7004)<br>Anti-mouse CD44 (https://www.biolegend.com/en-us/products/purified-anti-mouse-human-cd44-antibody-318)<br>Anti-mouse CD62L (https://www.biolegend.com/en-us/products/percp-cyanine5-5-anti-mouse-cd62l-antibody-4272)<br>Anti-mouse CD69 (https://www.biolegend.com/en-us/products/pe-anti-mouse-cd69-antibody-265)<br>Anti-mouse CD45 (https://www.biolegend.com/en-us/products/apc-cyanine7-anti-mouse-cd45-antibody-2530)<br>Anti-mouse CD8β (https://bxcell.com/product/m-lyt-3-2-ly-3-2/)<br>Anti-mouse CD4 (https://bxcell.com/product/m-cd4/)<br>H-2D(b) STHVNHLHC PE-Labeled Tetramer (SPAS-1) Synthesized from NIH Tetramer Core Facility<br>H-2K(b) SIINFEKL Brilliant Violet 421-Labeled control Tetramer synthesized from NIH Tetramer Core Facility<br>Alexa Fluor® 488 Anti-rabbit secondary antibody (https://www.thermofisher.com/antibody/product/Goat-anti-Rabbit-IgG-H-L-Cross-Adsorbed-Secondary-Antibody-Polyclonal/A-11070)<br>Anti-CSK (https://www.scbt.com/p/csk-antibody-e-3)<br>Anti-FLAG (https://www.sigmaaldrich.com/US/en/product/sigma/f3165)<br>Anti-HA (https://www.scbt.com/p/ha-probe-antibody-f-7?requestFrom=search)<br>Anti-MYC (https://www.cellsignal.com/products/primary-antibodies/myc-tag-9b11-mouse-mab/2276)<br>Anti-pTyr (https://www.scbt.com/p/p-tyr-antibody-py20?requestFrom=search)<br>Anti-ACK1 (https://www.scbt.com/p/ack-antibody-a-11?requestFrom=search)<br>Anti-PD-1 (https://www.novusbio.com/products/pd-1-antibody_nbp1-77276)<br>Anti-LCKpY394 (https://www.thermofisher.com/antibody/product/Phospho-LCK-Tyr394-Antibody-Polyclonal/PA5-37628)<br>Anti-LCKpY505 (https://www.cellsignal.com/products/primary-antibodies/phospho-lck-tyr505-antibody/2751)<br>Anti-LATpY132 (https://www.thermofisher.com/antibody/product/Phospho-LAT-Tyr132-Antibody-Polyclonal/44-224)<br>Anti-pZap70 (https://www.scbt.com/p/p-zap-70-antibody-py319-17a?requestFrom=search)<br>Anti-PLCgpY783 (https://www.cellsignal.com/products/primary-antibodies/phospho-plcg1-tyr783-antibody/2821?site-search-type=Products&N=4294956287&Ntt=2821p&fromPage=plp&_requestid=2573643)<br>Anti-cbp/PAG1 (https://www.scbt.com/p/cbp-antibody-g-8?requestFrom=search)<br>Anti-ACK1pY284 (https://www.emdmillipore.com/US/en/product/Anti-phospho-ACK1-Tyr284-Antibody,MM_NF-09-142)<br>Anti-LCK (https://www.scbt.com/p/lck-antibody-b-10?requestFrom=search)<br>Anti-Ub (https://www.scbt.com/p/ubiquitin-antibody-p4d1?requestFrom=search)<br>Anti-Actin Sigma (https://www.sigmaaldrich.com/US/en/product/sigma/a2228)<br>Anti-Perforin (https://www.biolegend.com/en-us/products/fitc-anti-mouse-perforin-antibody-21795)<br>Anti-FOXP3 (https://www.biolegend.com/en-us/products/pe-anti-mouse-rat-human-foxp3-antibody-2889)

Eukaryotic cell lines

Policy information about cell lines and Sex and Gender in Research

Cell line source(s)	TRAMP-C2, LNCaP-C4-2B, LAPC4, HEK293T, Jurkat cells were obtained from ATCC.
Authentication	Identities of all cell lines were confirmed by Short Tandem Repeat (STR) Profiling.
Mycoplasma contamination	All cultures were tested for mycoplasma contamination every 2 months using the PCR Mycoplasma Test Kit I/C (PromoKine). Mycoplasma testing was negative.
Commonly misidentified lines<br>(See ICLAC register)	None

Palaeontology and Archaeology

Specimen provenance	*Provide provenance information for specimens and describe permits that were obtained for the work (including the name of the*

Specimen provenance	*issuing authority, the date of issue, and any identifying information). Permits should encompass collection and, where applicable, export.*
Specimen deposition	*Indicate where the specimens have been deposited to permit free access by other researchers.*
Dating methods	*If new dates are provided, describe how they were obtained (e.g. collection, storage, sample pretreatment and measurement), where they were obtained (i.e. lab name), the calibration program and the protocol for quality assurance OR state that no new dates are provided.*

☐ Tick this box to confirm that the raw and calibrated dates are available in the paper or in Supplementary Information.

Ethics oversight	*Identify the organization(s) that approved or provided guidance on the study protocol, OR state that no ethical approval or guidance was required and explain why not.*

Note that full information on the approval of the study protocol must also be provided in the manuscript.

Animals and other research organisms

Policy information about studies involving animals; ARRIVE guidelines recommended for reporting animal research, and Sex and Gender in Research

Laboratory animals	6-week-old male C57BL/6 (Strain code: 027) mice were purchased from Charles River Laboratories. Ack1 KO mice and their WT counterparts were bred in house. 6-8 weeks old Ack1 KO and WT mice were used for the experiment. 10-12 weeks old NSG mice (Strain number: 005557) were used for the adoptive transfer experiment, purchased from The Jackson Laboratory. All mice were co-housed with 3-5 mice per cage and maintained in a controlled pathogen-free/germ-free environment with a temperature of 20–23° C, 12/12 h light/dark cycle, 50–60% humidity, and food and water provided ad libitum. Number mice used per experiments are described in the manuscript.<br><br>Customized polyclonal phosphorylated CSK antibody was made by GenScript, NJ using two 10-14 weeks old New Zealand Rabbits. All animals were handled according to the standards for humane care in accordance with the National Institutes of Health (NIH) Guide for the Care and Use of Laboratory Animals.
Wild animals	The study did not involve wild animals.
Reporting on sex	The study focuses on prostate cancer and immune modulation and male mice were used for all the tumor induction experiments in the study. While initial screening of the phenotype, both male and female Ack1 KO and WT mice were used without any gender bias. However, later predominantly male mice were used.
Field-collected samples	No field collected samples were used in the study
Ethics oversight	All animal studies were performed under approved Institutional Animal Care and Use Committee protocols at Washington University in St. Louis; Approved IACUC protocol #; 20180247, 20180259 and 20-0383.

Note that full information on the approval of the study protocol must also be provided in the manuscript.

Dual use research of concern

Policy information about dual use research of concern

Hazards

Could the accidental, deliberate or reckless misuse of agents or technologies generated in the work, or the application of information presented in the manuscript, pose a threat to:

No	Yes	
☒	☐	Public health
☒	☐	National security
☒	☐	Crops and/or livestock
☒	☐	Ecosystems
☒	☐	Any other significant area

Experiments of concern

Does the work involve any of these experiments of concern:

No	Yes	
☒	☐	Demonstrate how to render a vaccine ineffective
☒	☐	Confer resistance to therapeutically useful antibiotics or antiviral agents
☒	☐	Enhance the virulence of a pathogen or render a nonpathogen virulent
☒	☐	Increase transmissibility of a pathogen
☒	☐	Alter the host range of a pathogen
☒	☐	Enable evasion of diagnostic/detection modalities
☒	☐	Enable the weaponization of a biological agent or toxin
☒	☐	Any other potentially harmful combination of experiments and agents

ChIP-seq

Data deposition

☐ Confirm that both raw and final processed data have been deposited in a public database such as GEO.

☐ Confirm that you have deposited or provided access to graph files (e.g. BED files) for the called peaks.

Data access links<br>*May remain private before publication.*	*For "Initial submission" or "Revised version" documents, provide reviewer access links. For your "Final submission" document, provide a link to the deposited data.*
Files in database submission	*Provide a list of all files available in the database submission.*
Genome browser session<br>(e.g. UCSC)	*Provide a link to an anonymized genome browser session for "Initial submission" and "Revised version" documents only, to enable peer review. Write "no longer applicable" for "Final submission" documents.*

Methodology

Replicates	*Describe the experimental replicates, specifying number, type and replicate agreement.*
Sequencing depth	*Describe the sequencing depth for each experiment, providing the total number of reads, uniquely mapped reads, length of reads and whether they were paired- or single-end.*
Antibodies	*Describe the antibodies used for the ChIP-seq experiments; as applicable, provide supplier name, catalog number, clone name, and lot number.*
Peak calling parameters	*Specify the command line program and parameters used for read mapping and peak calling, including the ChIP, control and index files used.*
Data quality	*Describe the methods used to ensure data quality in full detail, including how many peaks are at FDR 5% and above 5-fold enrichment.*
Software	*Describe the software used to collect and analyze the ChIP-seq data. For custom code that has been deposited into a community repository, provide accession details.*

Flow Cytometry

Plots

Confirm that:

☒ The axis labels state the marker and fluorochrome used (e.g. CD4-FITC).

☒ The axis scales are clearly visible. Include numbers along axes only for bottom left plot of group (a 'group' is an analysis of identical markers).

☒ All plots are contour plots with outliers or pseudocolor plots.

☒ A numerical value for number of cells or percentage (with statistics) is provided.

Methodology

Sample preparation	Under sterile conditions, spleen, lymph nodes and femurs were harvested from naïve or TRAMP-C2 tumor bearing WT and Ack1 KO mice. Single cells were made and RBCs were lysed using ACK lysis buffer. For immunophenotyping, 1x106 cells were incubated with Live/Dead Aqua (1:800), anti-CD3 PECy7 (1:400), anti-CD4 Pacific blue (1:400), anti CD8 APC (1:400) antibodies to identify T-cells. The anti-CD19 PerCP-Cy 5.5 antibody (1:400) was used to identify B-cells, anti-NK1.1 PE antibody (1:500) was used to identify NK cells, anti-CD4 Pacific blue (1:400), anti-CD25 FITC (1:250) and intracellular anti-

FoxP3 PE (1:250) antibodies were used to identify regulatory T cells, anti-CD11b PerCP-Cy 5.5 (1:400) and anti-Gr-1 APC (1:400) antibodies were used to identify subset of myeloid cells. Antibodies were incubated for 20 minutes, according to manufacturer's instructions (BD biosciences and BioLegend). T-cells were purified from splenocytes using mouse CD3+ T Cell Enrichment Column (R&D Systems) according to manufacturer's protocol. Purified T-cells or splenocytes were stained with Live/Dead Aqua (1:800) and either anti-CD3 PECy7 (1:400), anti CD8 APC (1:400) and activation markers - anti-CD137 FITC (1:400), anti-CD44 PE (1:400) and anti-CD62L Percpcy5.5 (1:400) or exhaustion markers - anti-PD1 FITC (1:400), anti-Lag3 Percpcy5.5 (1:400), anti-Tim3 PE (1:400) or anti-CD4 Pacific blue (1:400) with activation marker anti-CD69 PE (1:400) antibodies. Cells were then permeabilized and intracellular staining was done with anti-perforin FITC (1:300), anti-IL2 APC-Cy7 (1:300) or anti-IFN gamma BV786 (1:300) antibodies. Tumor infiltrating T cells were identified by staining with Live/Dead Aqua (1:800), anti-CD45 APC-Cy7 (1:300), anti-CD3 PECy7 (1:400), anti CD8 APC (1:400), anti-CD4 Pacific blue (1:400), anti-CD69 PE (1:400), anti-CD25 FITC (1:250) antibodies. Cells were then permeabilized and intracellular staining was done with anti-perforin FITC (1:300) and anti-FoxP3 PE (1:250) antibodies. Parallel immunophenotyping of spleen and tumor draining lymph nodes was performed in control and (R)-9b treated tumor bearing C57BL/6 mice staining with Live/Dead Aqua (1:800), anti-CD3, anti-CD4, Anti-CD8, activation markers, exhaustion markers and intracellular staining with antibodies for markers as mentioned above. Samples were analyzed using BD FACSCanto II or LSR Fortessa (BD Biosciences) and post-acquisition analysis was done using FlowJo software (Tree Star Inc).
Inhibition of pY18-CSK upon ACK1 knock down or pharmacological inhibition was assessed by flow cytometry. Briefly, Jurkat cells treated with vehicle or (R)-9b and splenocytes from WT and Ack1 KO mice were incubated with pTyr18-CSK primary antibody. Cells were washed, incubated with anti-rabbit Alexa Fluor® 488 antibody, fixed and flow cytometry was performed.

Instrument: Samples were analyzed using BD FACSCanto II and LSRII (BD Biosciences)

Software: Post-acquisition analysis was done using FlowJo software (Tree Star Inc)

Cell population abundance: The abundance of live cells was measured by counting using trypan blue dye exclusion method prior to flow staining.

Gating strategy: The T cells were identified gating on Live cells and anti-CD3 PECy7, followed by anti-CD4 Pacific blue and anti CD8 APC. For B, NK and MDSCs and Tregs cells, first live cells were gated, followed by the respective antibodies, e.g. anti-CD19 were used to identify B-cells, anti-NK1.1 to identify NK cells, anti-CD4 Pacific blue and anti-CD25 to identify regulatory T cells, anti-CD11b and anti-Gr-1 to identify myeloid derived suppressor cells.
Purified T-cells or splenocytes or cells from lymph nodes were gated with live cells and CD3 PECy7, followed by anti CD8 APC and activation markers - anti-CD137 FITC, anti-CD44 PE and anti-CD62L Percpcy5.5 or anti-CD4 Pacific blue with activation marker anti-CD69 PE.
For the SPAS-1 tetramer, the cells were gated for CD45+, followed by CD3+ and SPAS-1+.
For tumor infiltrating cells (TILS), the cells were gated on Live cells, CD45+, followed by CD4+ and CD8 + gating. The subtypes of activated and regulatory T cells were identified by CD44/CD62L and CD4+ CD25+FOXP3+ gating respectively. A detailed explanation on gating is provided in the manuscript wherever necessary and shown in the supplementary figures 7c, 8a-c, 10a and source data.

☒ Tick this box to confirm that a figure exemplifying the gating strategy is provided in the Supplementary Information.

Magnetic resonance imaging

Experimental design

Design type: *Indicate task or resting state; event-related or block design.*

Design specifications: *Specify the number of blocks, trials or experimental units per session and/or subject, and specify the length of each trial or block (if trials are blocked) and interval between trials.*

Behavioral performance measures: *State number and/or type of variables recorded (e.g. correct button press, response time) and what statistics were used to establish that the subjects were performing the task as expected (e.g. mean, range, and/or standard deviation across subjects).*

Acquisition

Imaging type(s): *Specify: functional, structural, diffusion, perfusion.*

Field strength: *Specify in Tesla*

Sequence & imaging parameters: *Specify the pulse sequence type (gradient echo, spin echo, etc.), imaging type (EPI, spiral, etc.), field of view, matrix size, slice thickness, orientation and TE/TR/flip angle.*

Area of acquisition: *State whether a whole brain scan was used OR define the area of acquisition, describing how the region was determined.*

Diffusion MRI ☐ Used ☐ Not used

Preprocessing

Preprocessing software: *Provide detail on software version and revision number and on specific parameters (model/functions, brain extraction, segmentation, smoothing kernel size, etc.).*

Normalization | *If data were normalized/standardized, describe the approach(es): specify linear or non-linear and define image types used for transformation OR indicate that data were not normalized and explain rationale for lack of normalization.*

Normalization template | *Describe the template used for normalization/transformation, specifying subject space or group standardized space (e.g. original Talairach, MNI305, ICBM152) OR indicate that the data were not normalized.*

Noise and artifact removal | *Describe your procedure(s) for artifact and structured noise removal, specifying motion parameters, tissue signals and physiological signals (heart rate, respiration).*

Volume censoring | *Define your software and/or method and criteria for volume censoring, and state the extent of such censoring.*

Statistical modeling & inference

Model type and settings | *Specify type (mass univariate, multivariate, RSA, predictive, etc.) and describe essential details of the model at the first and second levels (e.g. fixed, random or mixed effects; drift or auto-correlation).*

Effect(s) tested | *Define precise effect in terms of the task or stimulus conditions instead of psychological concepts and indicate whether ANOVA or factorial designs were used.*

Specify type of analysis: ☐ Whole brain ☐ ROI-based ☐ Both

Statistic type for inference (See Eklund et al. 2016) | *Specify voxel-wise or cluster-wise and report all relevant parameters for cluster-wise methods.*

Correction | *Describe the type of correction and how it is obtained for multiple comparisons (e.g. FWE, FDR, permutation or Monte Carlo).*

Models & analysis

n/a	Involved in the study
☐	☐ Functional and/or effective connectivity
☐	☐ Graph analysis
☐	☐ Multivariate modeling or predictive analysis

Functional and/or effective connectivity | *Report the measures of dependence used and the model details (e.g. Pearson correlation, partial correlation, mutual information).*

Graph analysis | *Report the dependent variable and connectivity measure, specifying weighted graph or binarized graph, subject- or group-level, and the global and/or node summaries used (e.g. clustering coefficient, efficiency, etc.).*

Multivariate modeling and predictive analysis | *Specify independent variables, features extraction and dimension reduction, model, training and evaluation metrics.*
