# Supplementary material for: Inhibiting ACK1-mediated phosphorylation of C-terminal Src kinase counteracts prostate cancer immune checkpoint blockade resistance
Source: Nat Commun. 2022 Nov 14;13:6929. doi: 10.1038/s41467-022-34724-5 (PMC9663509; doi:10.1038/s41467-022-34724-5)
Supplement: Supplementary file 4 — Source Data [file 41467_2022_34724_MOESM4_ESM.zip › 331588_3_related_ms_7010045_rk2r4x.pdf]

Fig 1a

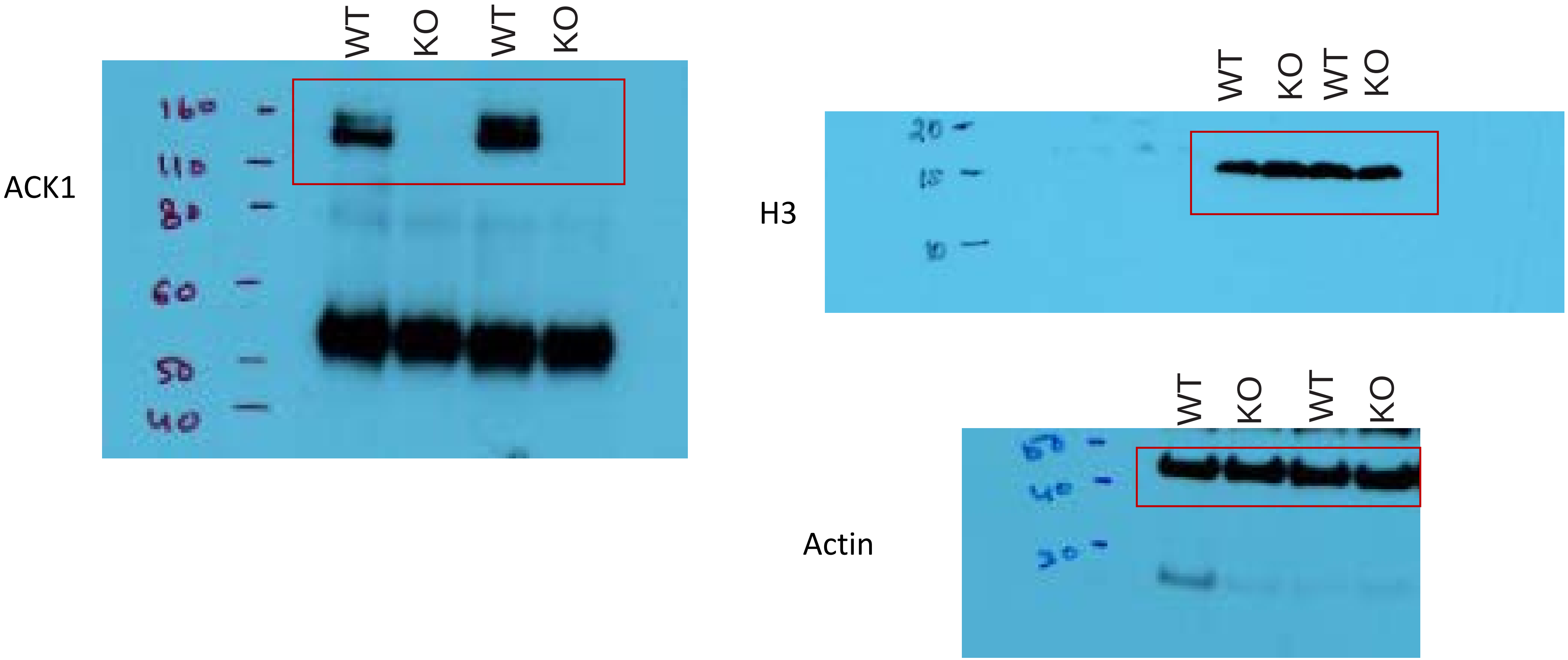

Fig 2a

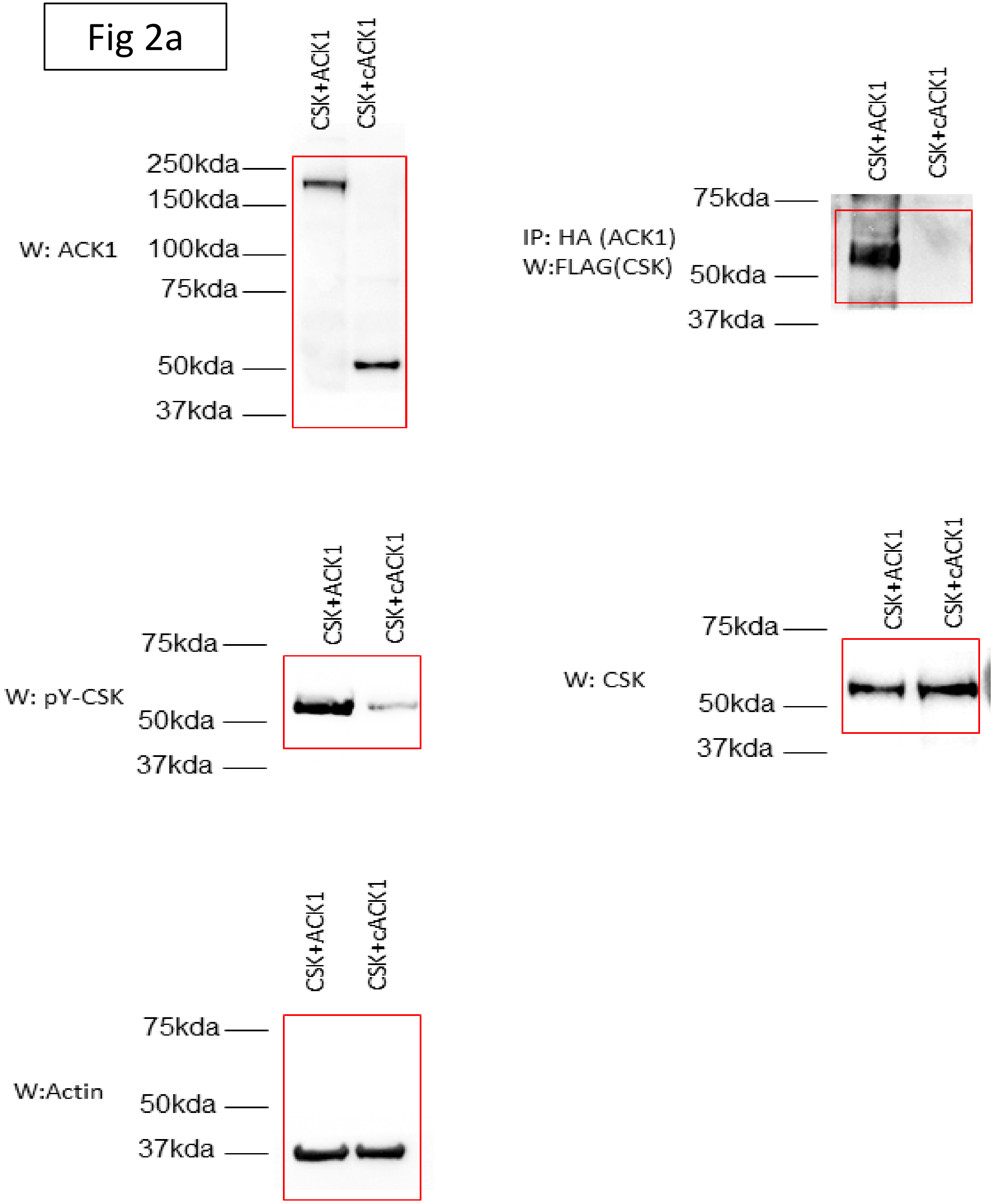
CSK+ACK1
CSK+cACK1
W: ACK1
250kda
150kda
100kda
75kda
50kda
37kda
IP: HA (ACK1)
W:FLAG(CSK)
75kda
50kda
37kda
W: pY-CSK
75kda
50kda
37kda
W: CSK
75kda
50kda
37kda
W:Actin
75kda
50kda
37kda

Fig 2c

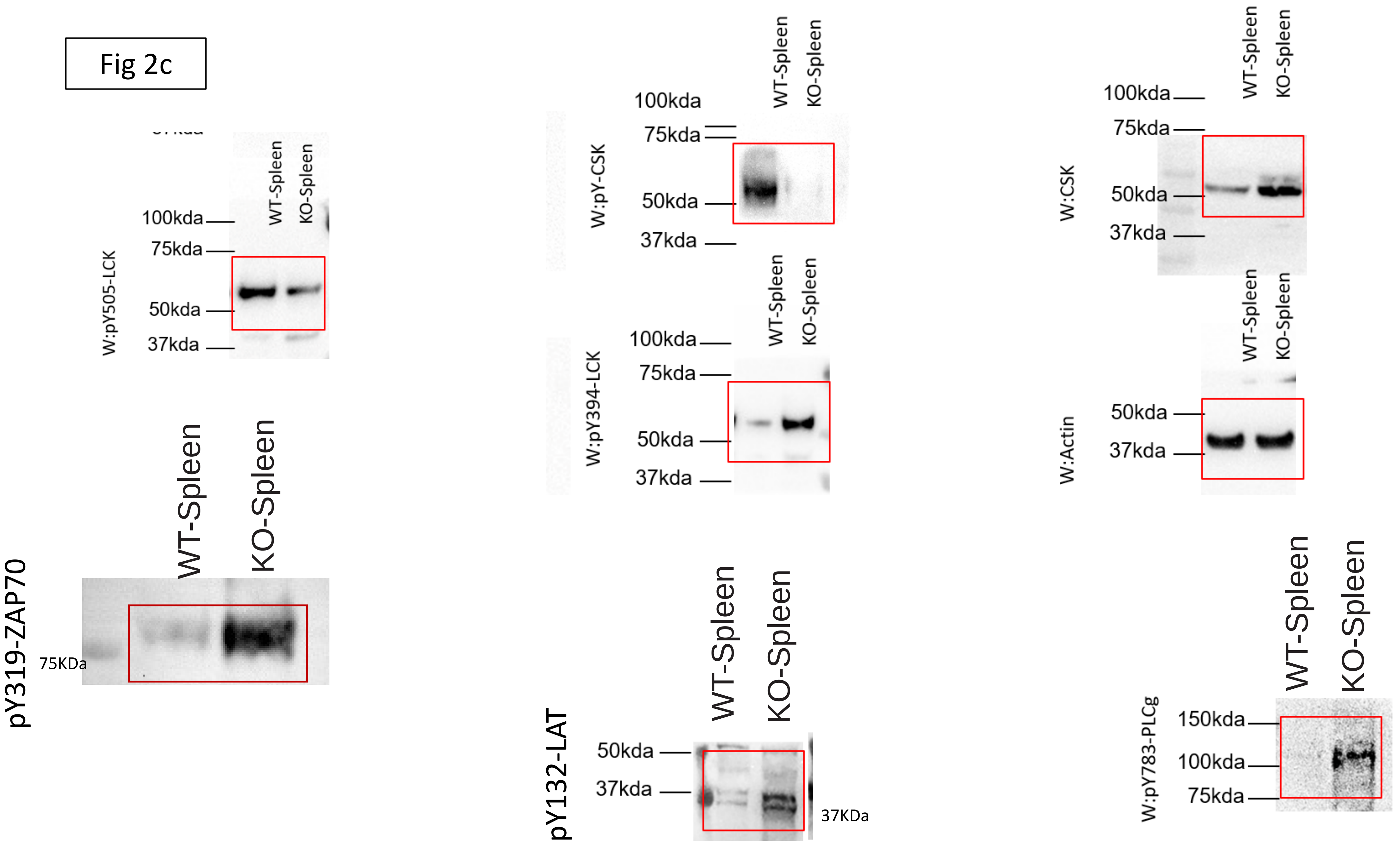
W:pY505-LCK
WT-Spleen
KO-Spleen
100kda
75kda
50kda
37kda
W:pY-CSK
WT-Spleen
KO-Spleen
100kda
75kda
50kda
37kda
W:CSK
WT-Spleen
KO-Spleen
100kda
75kda
50kda
37kda
W:pY394-LCK
WT-Spleen
KO-Spleen
100kda
75kda
50kda
37kda
W:Actin
WT-Spleen
KO-Spleen
50kda
37kda
pY319-ZAP70
WT-Spleen
KO-Spleen
75KDa
pY132-LAT
WT-Spleen
KO-Spleen
50kda
37kda
37KDa
W:pY783-PLCg
WT-Spleen
KO-Spleen
150kda
100kda
75kda

Fig 2d

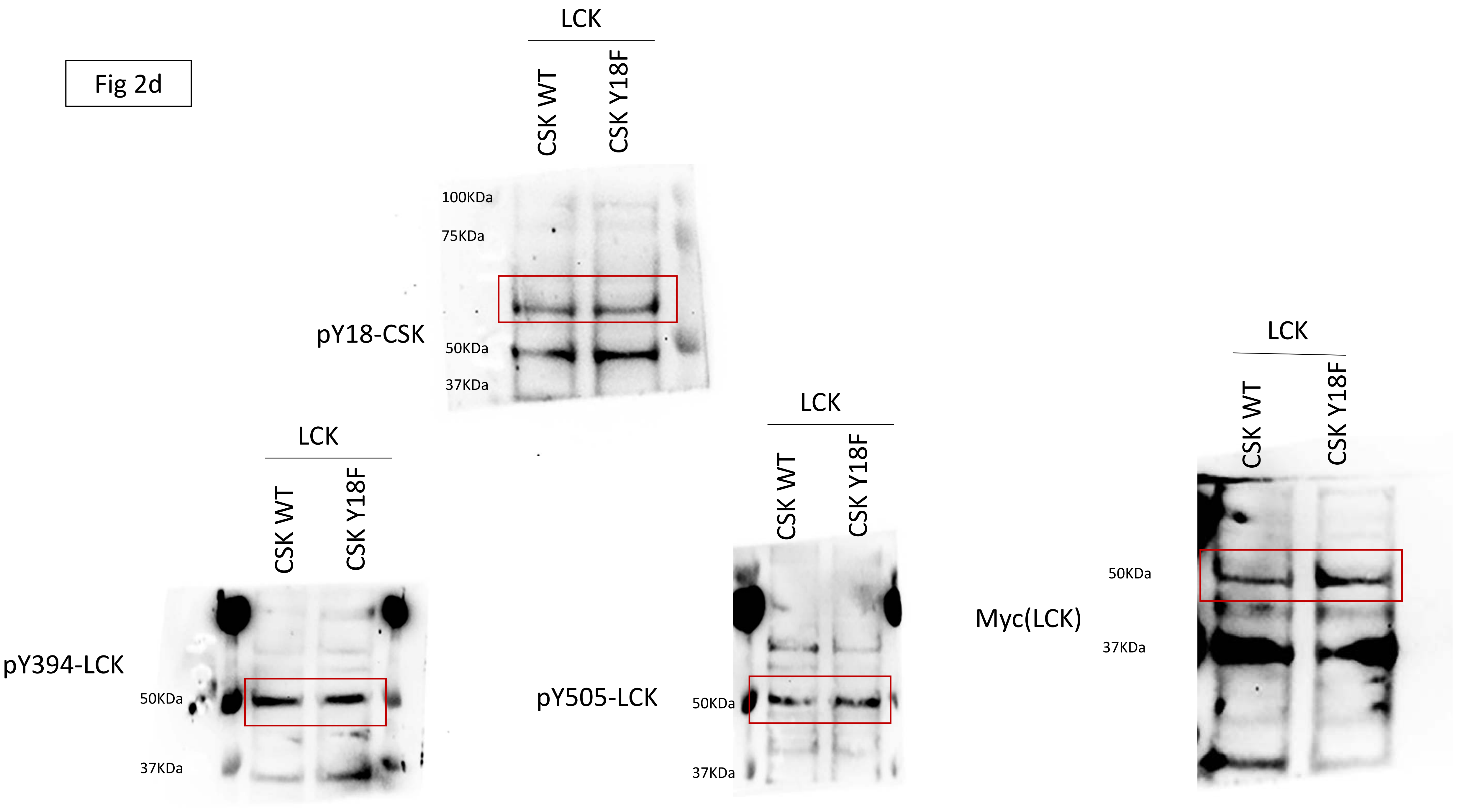
LCK
CSK WT
CSK Y18F
100KDa
75KDa
pY18-CSK
50KDa
37KDa
LCK
CSK WT
CSK Y18F
pY394-LCK
50KDa
37KDa
LCK
CSK WT
CSK Y18F
pY505-LCK
50KDa
37KDa
LCK
CSK WT
CSK Y18F
Myc(LCK)
50KDa
37KDa

Fig 2d

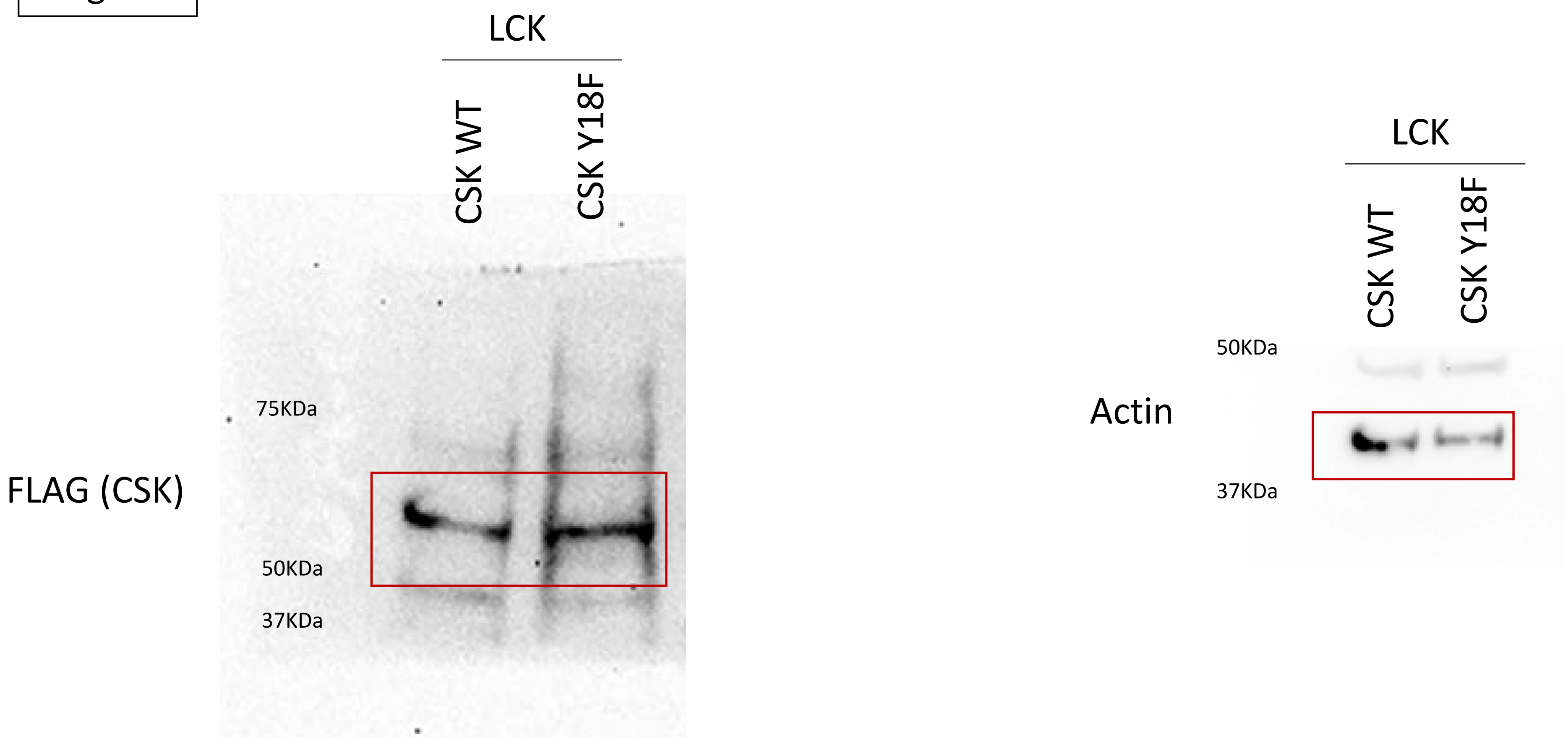

LCK
CSK WT
CSK Y18F
75KDa
FLAG (CSK)
50KDa
37KDa
LCK
CSK WT
CSK Y18F
50KDa
Actin
37KDa

Fig 2f

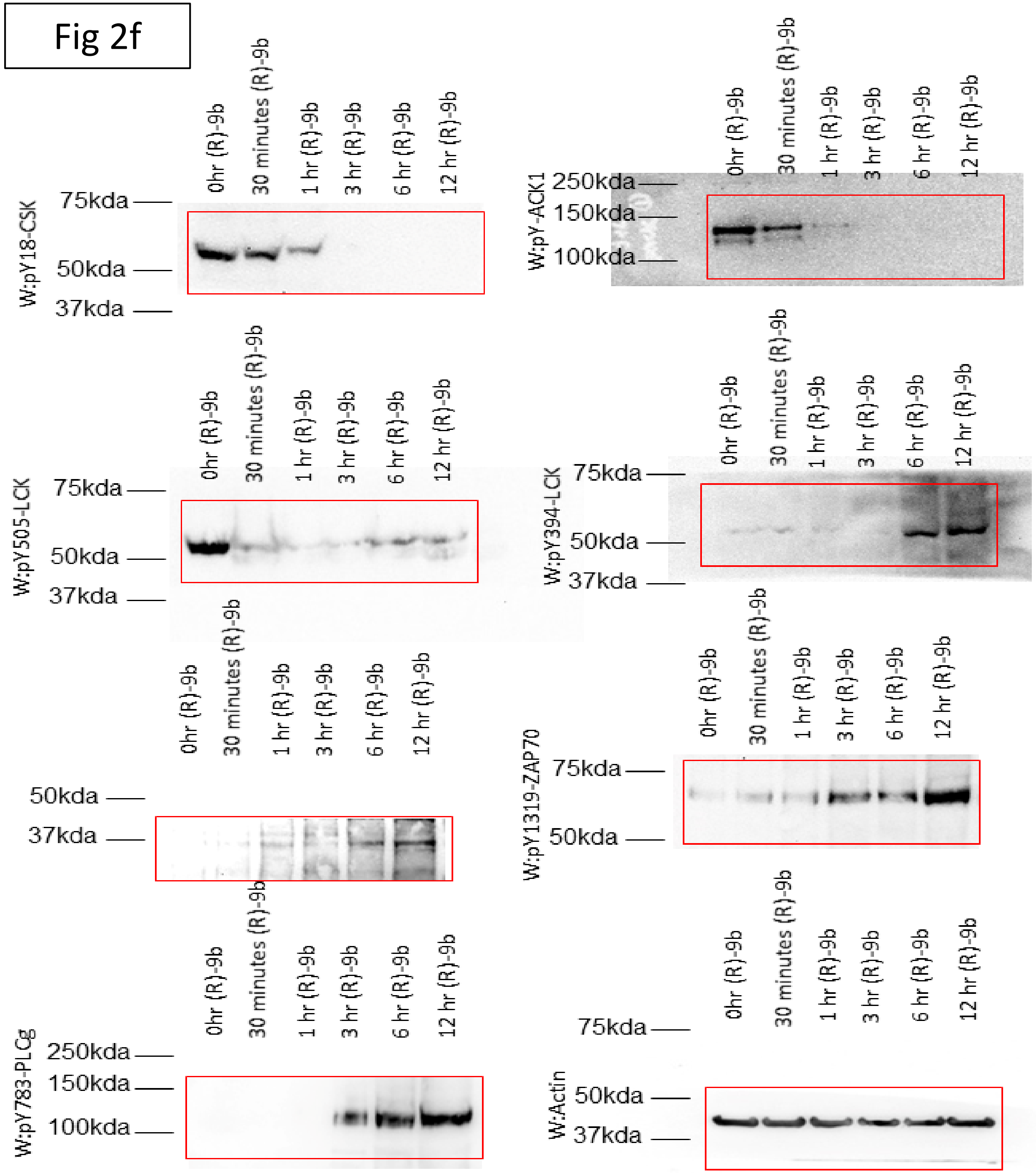

0hr (R)-9b
30 minutes (R)-9b
1 hr (R)-9b
3 hr (R)-9b
6 hr (R)-9b
12 hr (R)-9b
W:pY18-CSK
75kda
50kda
37kda
W:pY-ACK1
250kda
150kda
100kda
W:pY505-LCK
75kda
50kda
37kda
W:pY394-LCK
75kda
50kda
37kda
50kda
37kda
W:pY1319-ZAP70
75kda
50kda
W:pY783-PLCg
250kda
150kda
100kda
W:Actin
75kda
50kda
37kda

Fig 3a

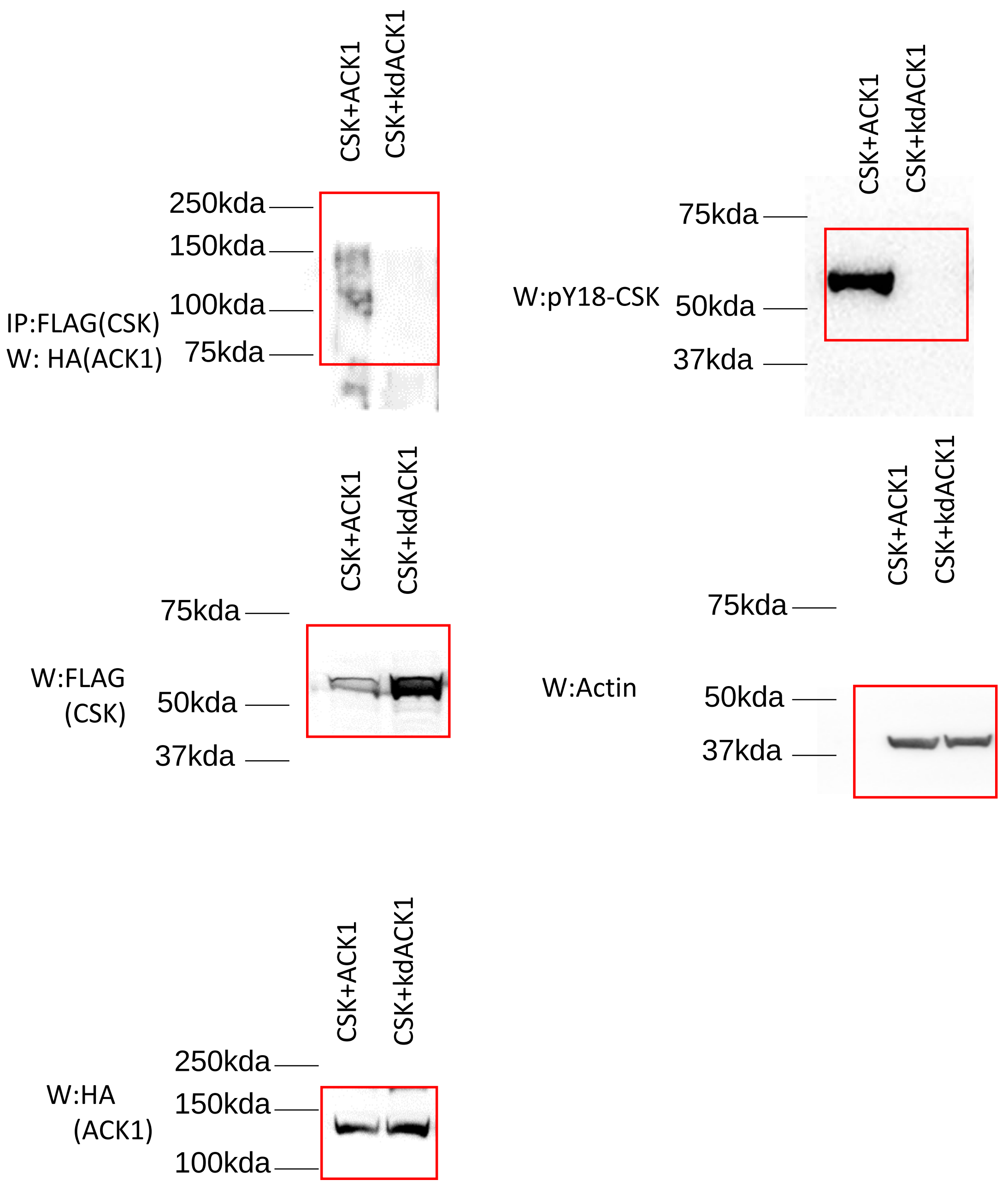
CSK+ACK1
CSK+kdACK1
250kda
150kda
100kda
75kda
IP:FLAG(CSK)
W: HA(ACK1)
W:pY18-CSK
75kda
50kda
37kda
W:FLAG
(CSK)
75kda
50kda
37kda
W:Actin
75kda
50kda
37kda
W:HA
(ACK1)
250kda
150kda
100kda

Fig 3b

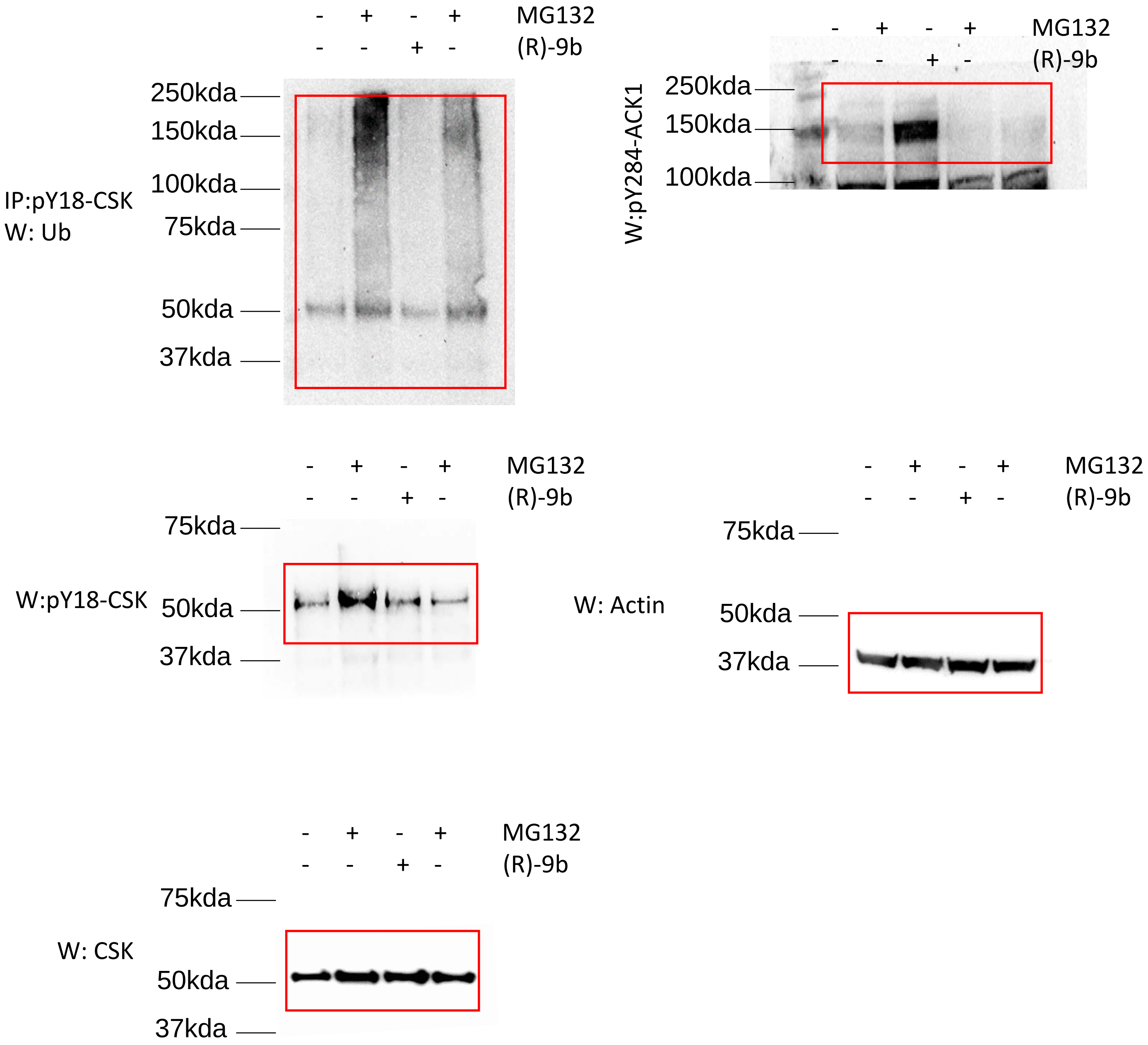

MG132
(R)-9b
IP:pY18-CSK
W: Ub
250kda
150kda
100kda
75kda
50kda
37kda
W:pY284-ACK1
W:pY18-CSK
W: Actin
W: CSK

Fig 3c

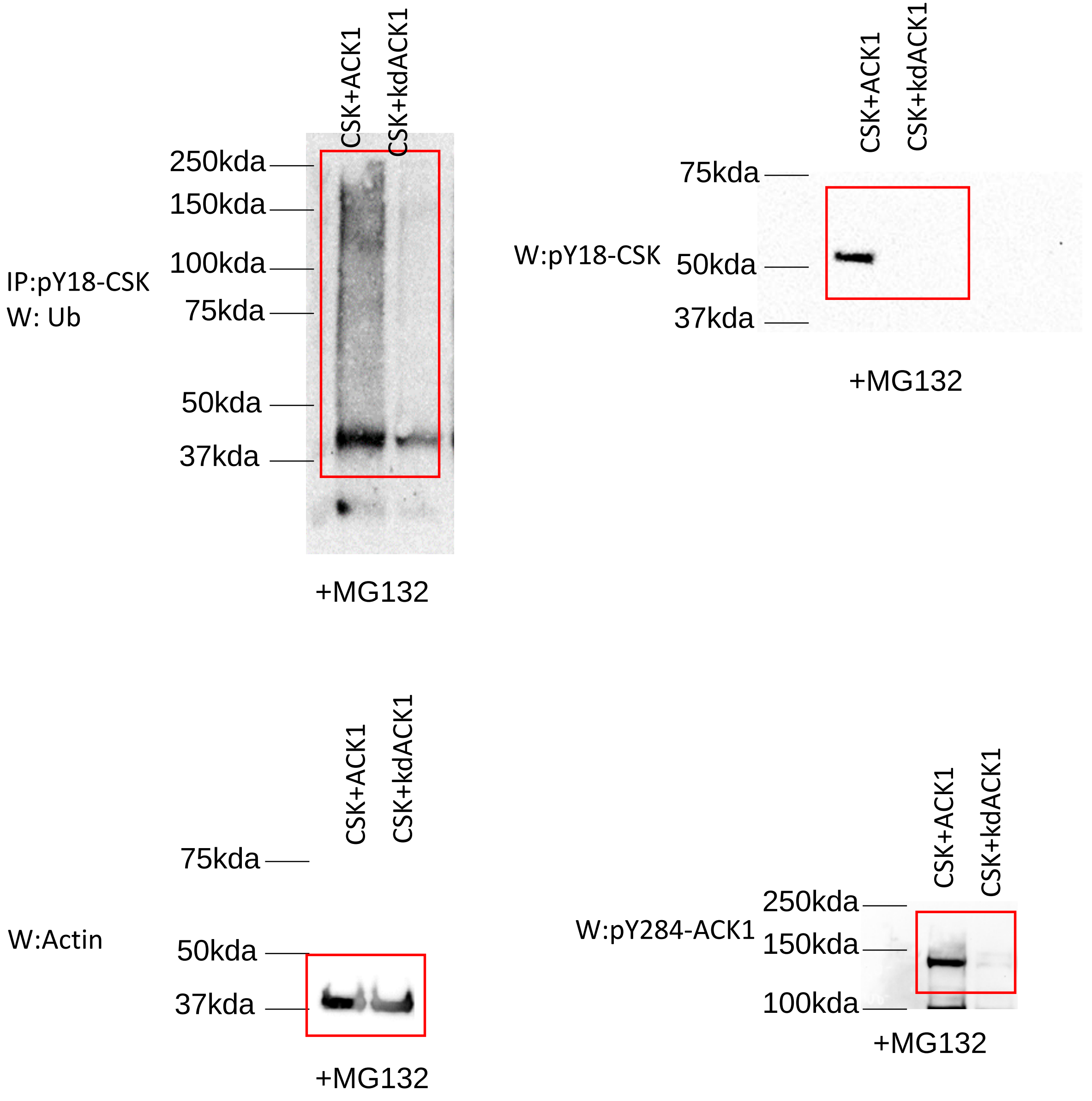
CSK+ACK1
CSK+kdACK1
250kda
150kda
100kda
75kda
50kda
37kda
IP:pY18-CSK
W: Ub
+MG132
CSK+ACK1
CSK+kdACK1
75kda
W:pY18-CSK
50kda
37kda
+MG132
CSK+ACK1
CSK+kdACK1
75kda
W:Actin
50kda
37kda
+MG132
CSK+ACK1
CSK+kdACK1
250kda
W:pY284-ACK1
150kda
100kda
+MG132

Fig 3d

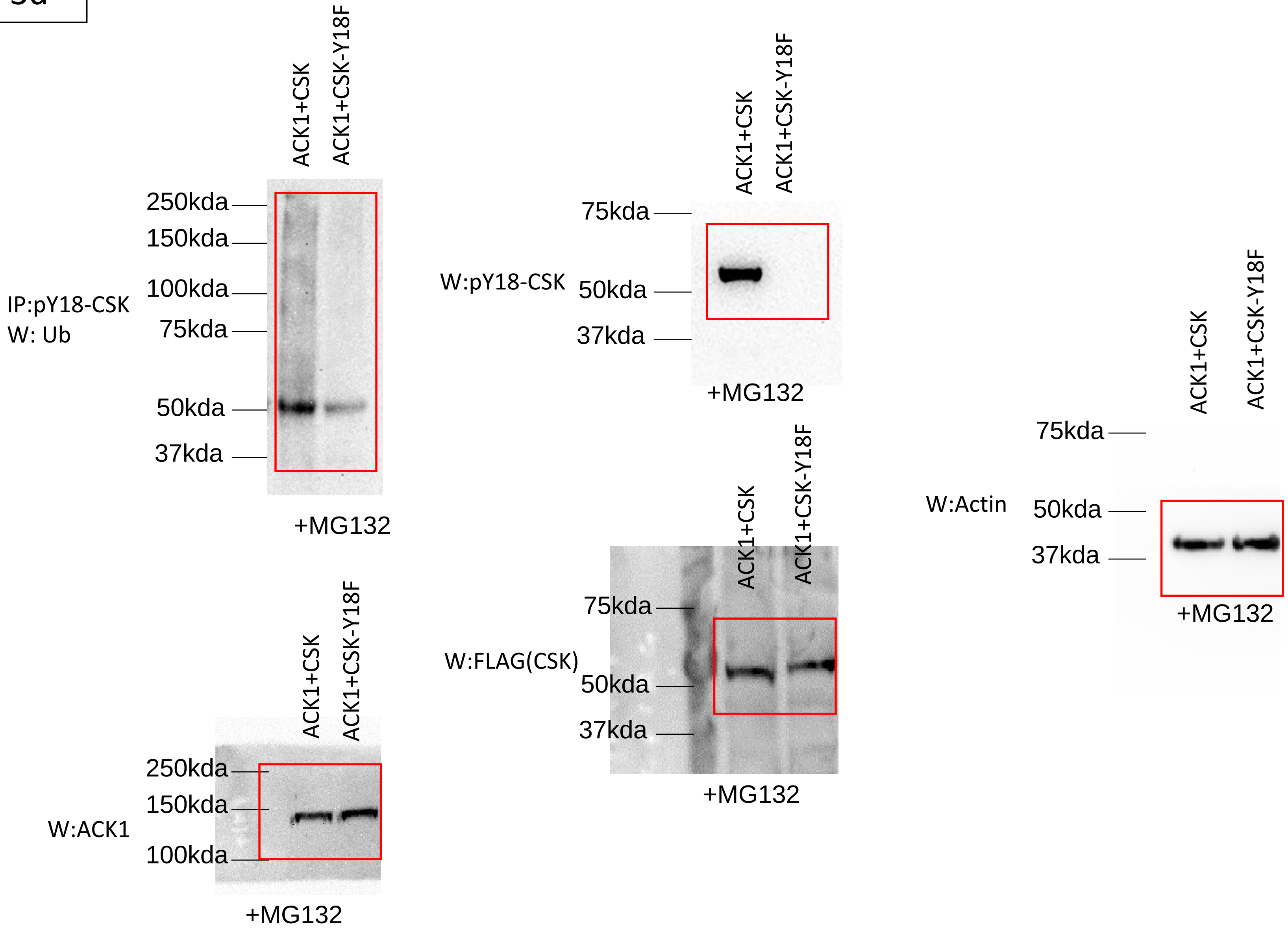
IP:pY18-CSK
W: Ub
ACK1+CSK
ACK1+CSK-Y18F
250kda
150kda
100kda
75kda
50kda
37kda
+MG132
W:pY18-CSK
ACK1+CSK
ACK1+CSK-Y18F
75kda
50kda
37kda
+MG132
W:Actin
ACK1+CSK
ACK1+CSK-Y18F
75kda
50kda
37kda
+MG132
W:ACK1
ACK1+CSK
ACK1+CSK-Y18F
250kda
150kda
100kda
+MG132
W:FLAG(CSK)
ACK1+CSK
ACK1+CSK-Y18F
75kda
50kda
37kda
+MG132

Fig 3e

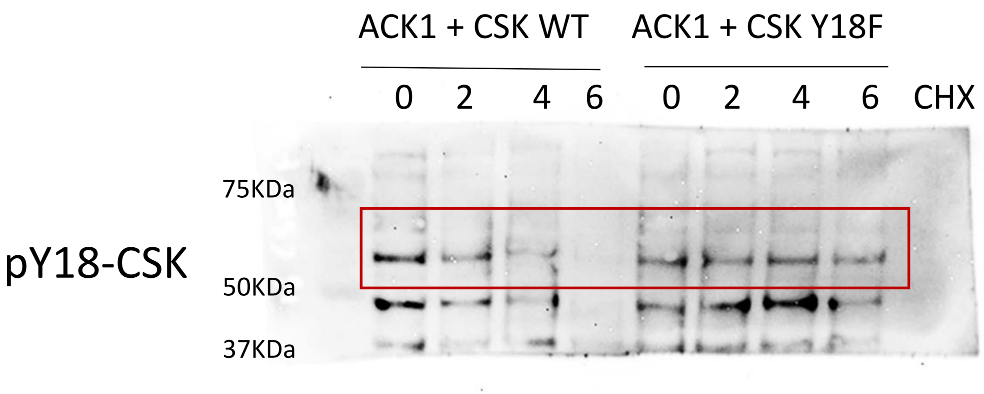
ACK1 + CSK WT
ACK1 + CSK Y18F
0 2 4 6 0 2 4 6 CHX
75KDa
pY18-CSK
50KDa
37KDa

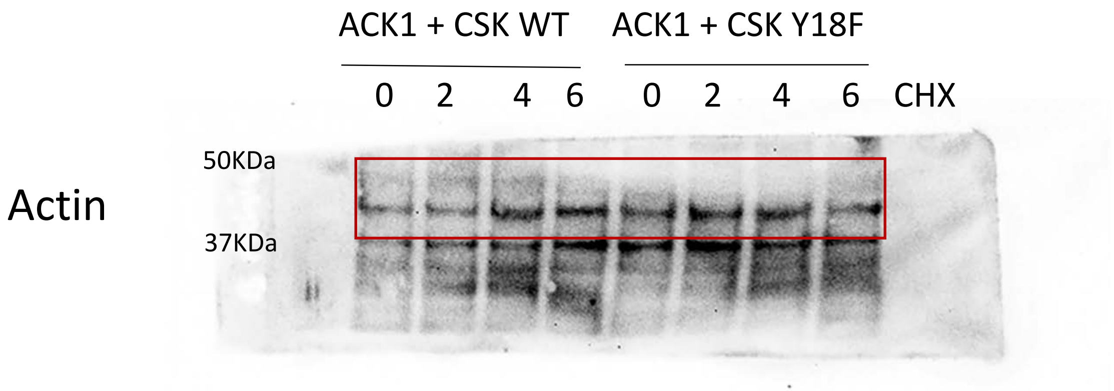
ACK1 + CSK WT
ACK1 + CSK Y18F
0 2 4 6 0 2 4 6 CHX
50KDa
Actin
37KDa

Fig 3f

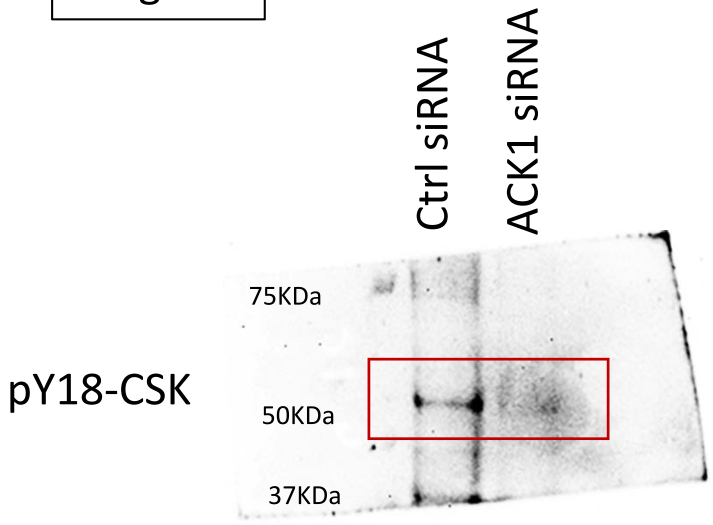

Ctrl siRNA
ACK1 siRNA
pY18-CSK
75KDa
50KDa
37KDa

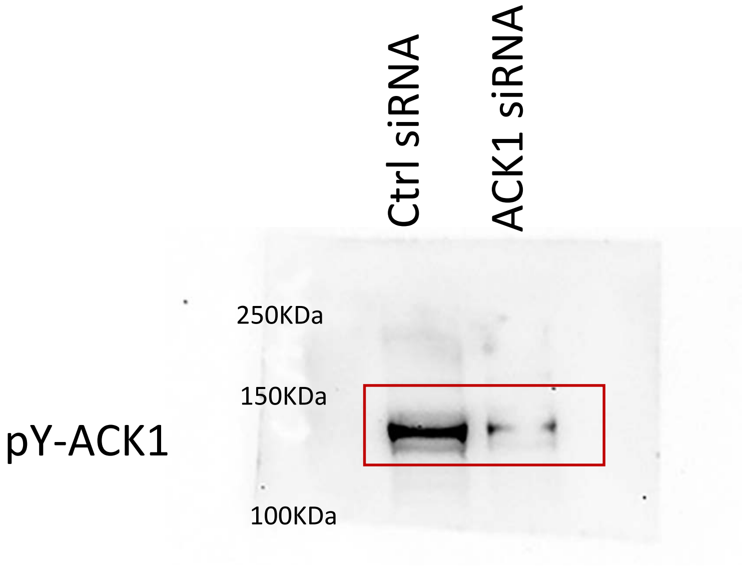

Ctrl siRNA
ACK1 siRNA
pY-ACK1
250KDa
150KDa
100KDa

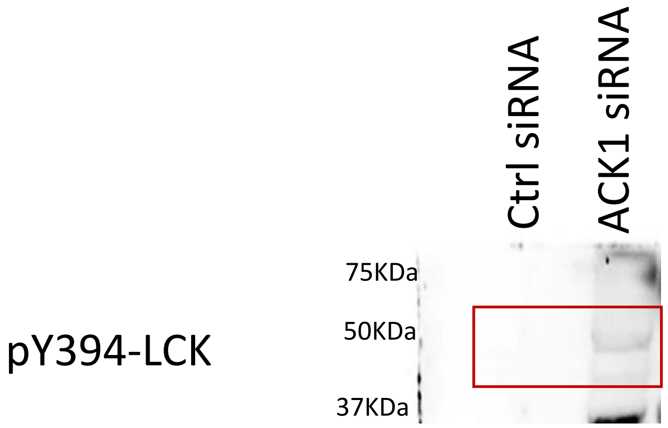

Ctrl siRNA
ACK1 siRNA
pY394-LCK
75KDa
50KDa
37KDa

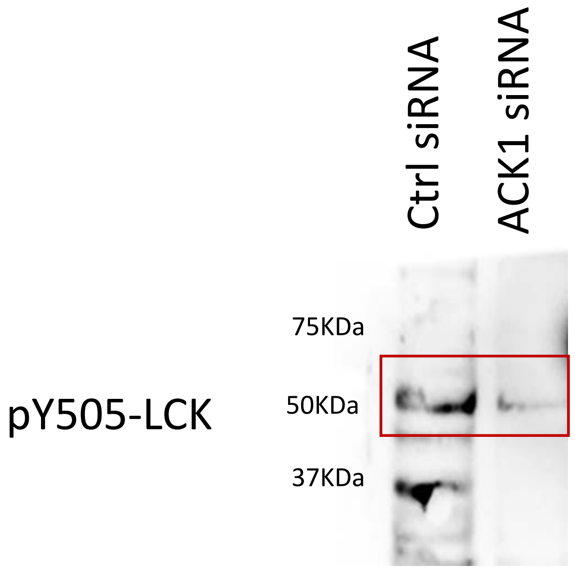

Ctrl siRNA
ACK1 siRNA
pY505-LCK
75KDa
50KDa
37KDa

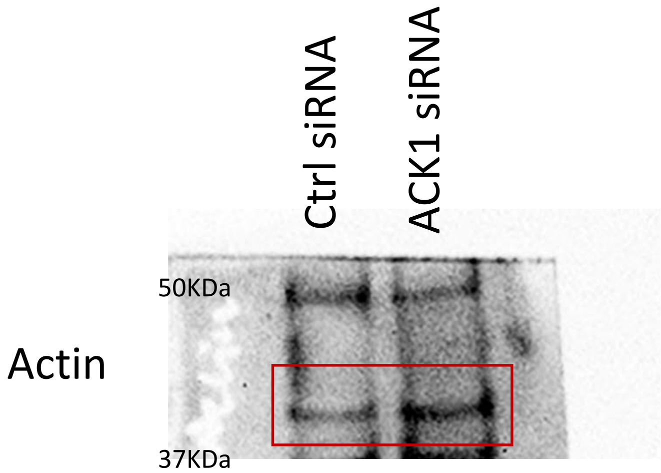

Ctrl siRNA
ACK1 siRNA
Actin
50KDa
37KDa

Fig 6f

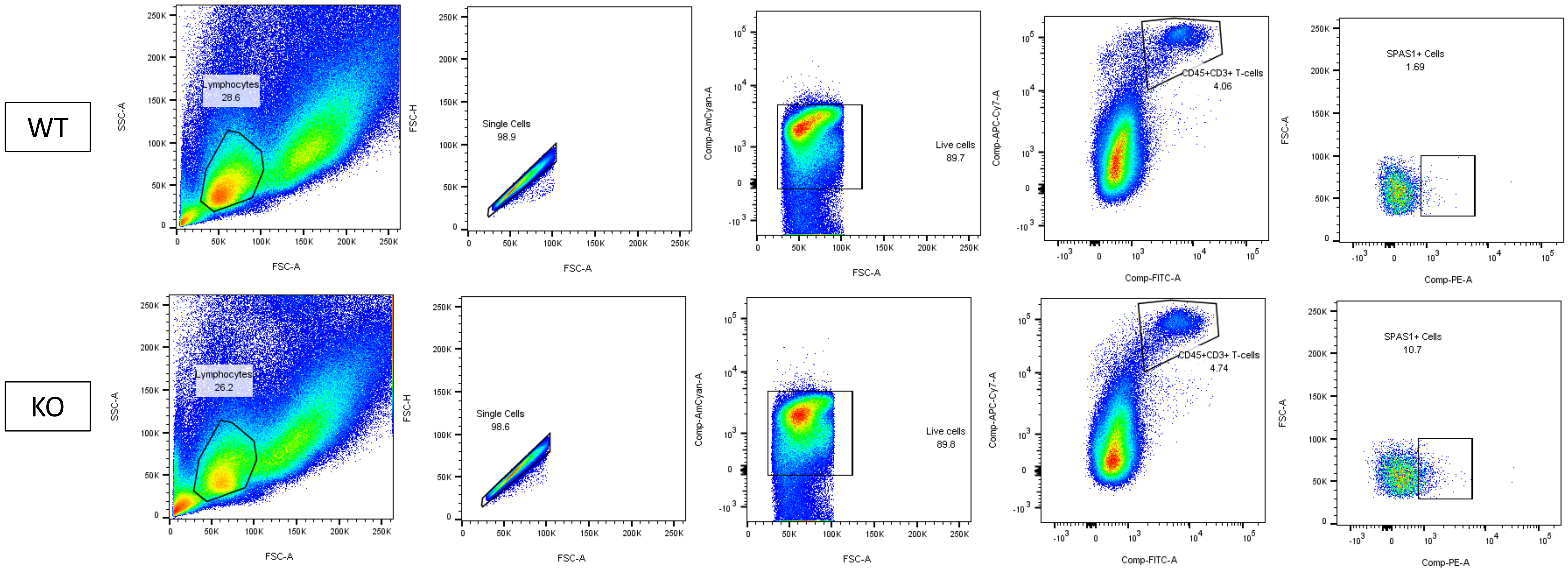

Adoptive transfer - splenocytes were isolated and the levels of SPAS-1 expression was assessed by flow cytometry

Fig 6g

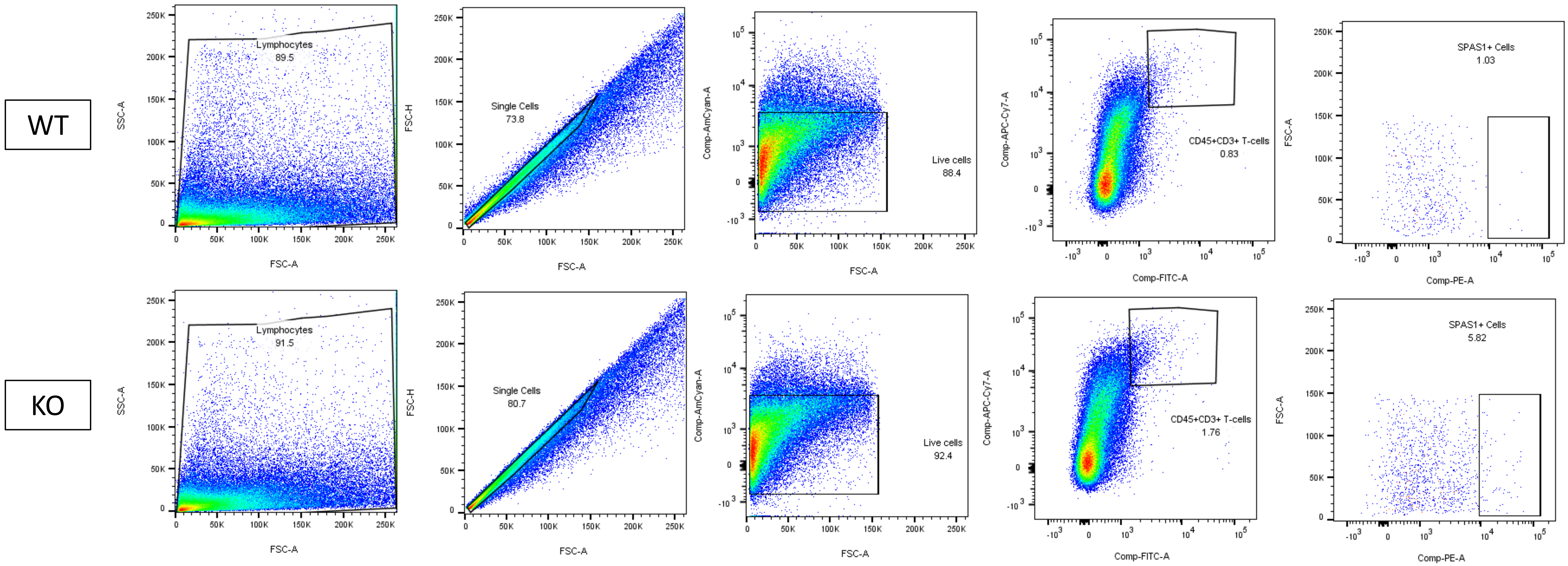

Adoptive transfer - lymph nodes were drained and the levels of SPAS-1 expression was assessed by flow cytometry

Fig 7a

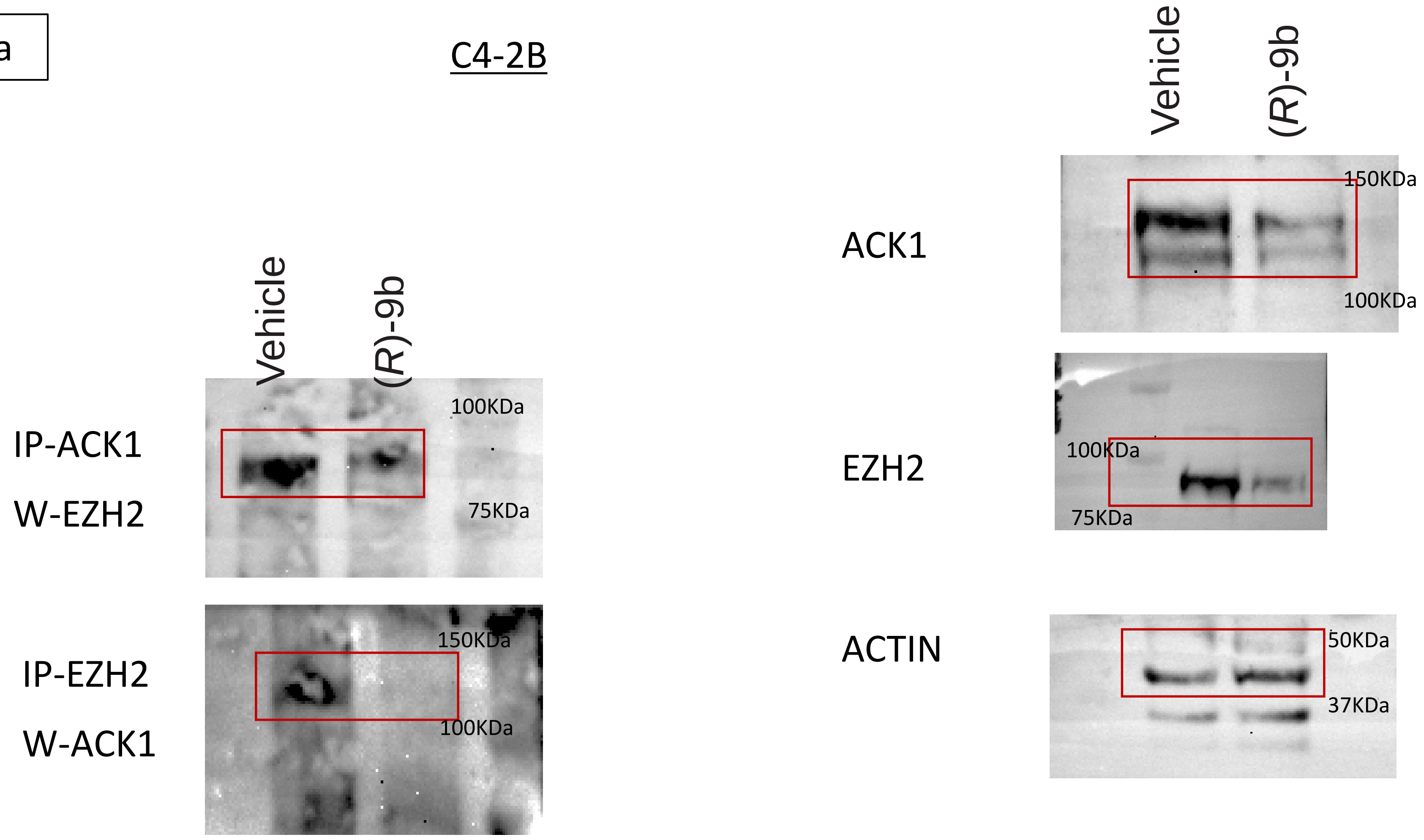

C4-2B
Vehicle
(R)-9b
IP-ACK1
W-EZH2
100KDa
75KDa
IP-EZH2
W-ACK1
150KDa
100KDa
Vehicle
(R)-9b
ACK1
150KDa
100KDa
EZH2
100KDa
75KDa
ACTIN
50KDa
37KDa

Fig 7a

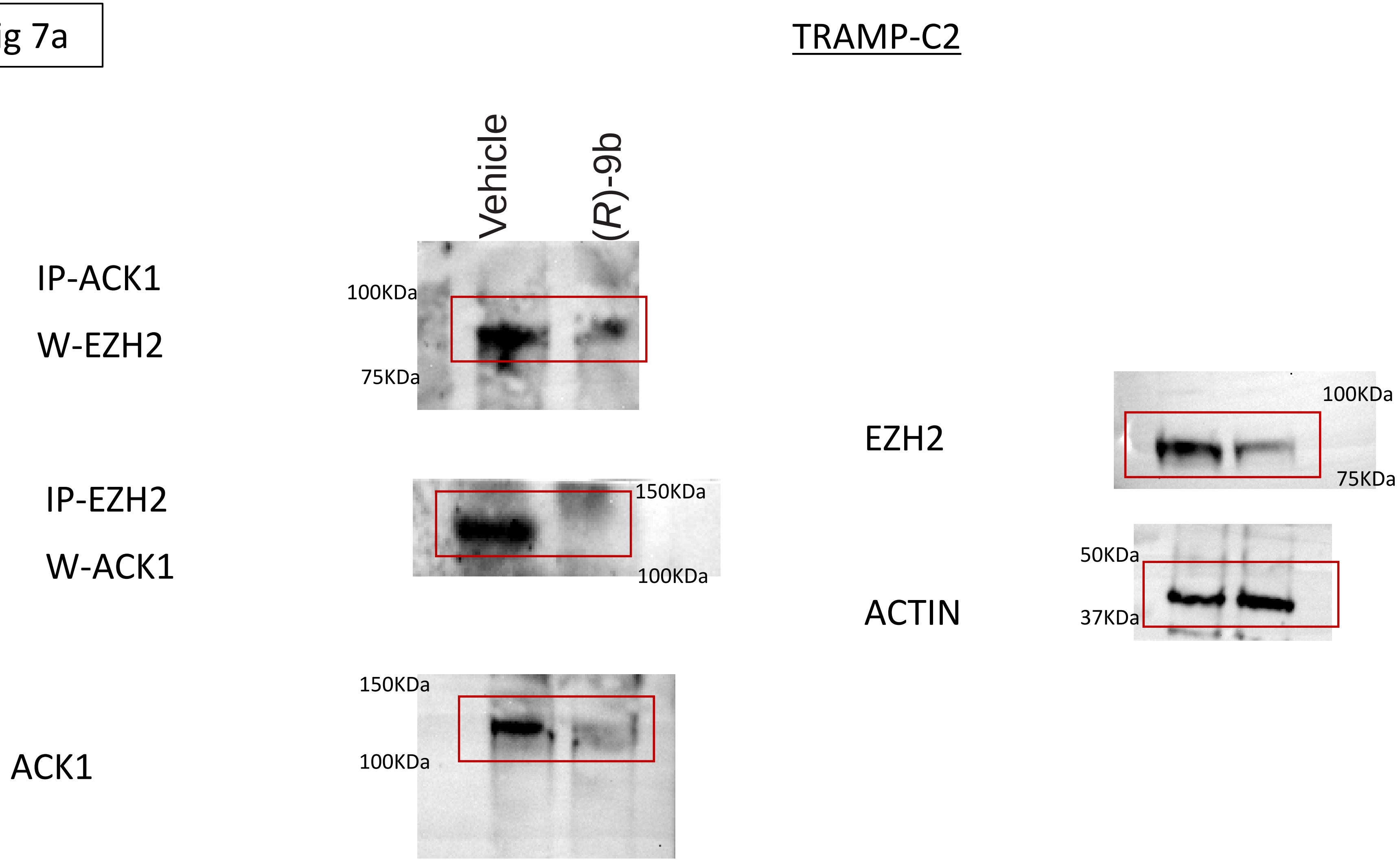
TRAMP-C2
Vehicle
(R)-9b
IP-ACK1
W-EZH2
100KDa
75KDa
IP-EZH2
W-ACK1
150KDa
100KDa
ACK1
150KDa
100KDa
EZH2
100KDa
75KDa
ACTIN
50KDa
37KDa

Fig 7f

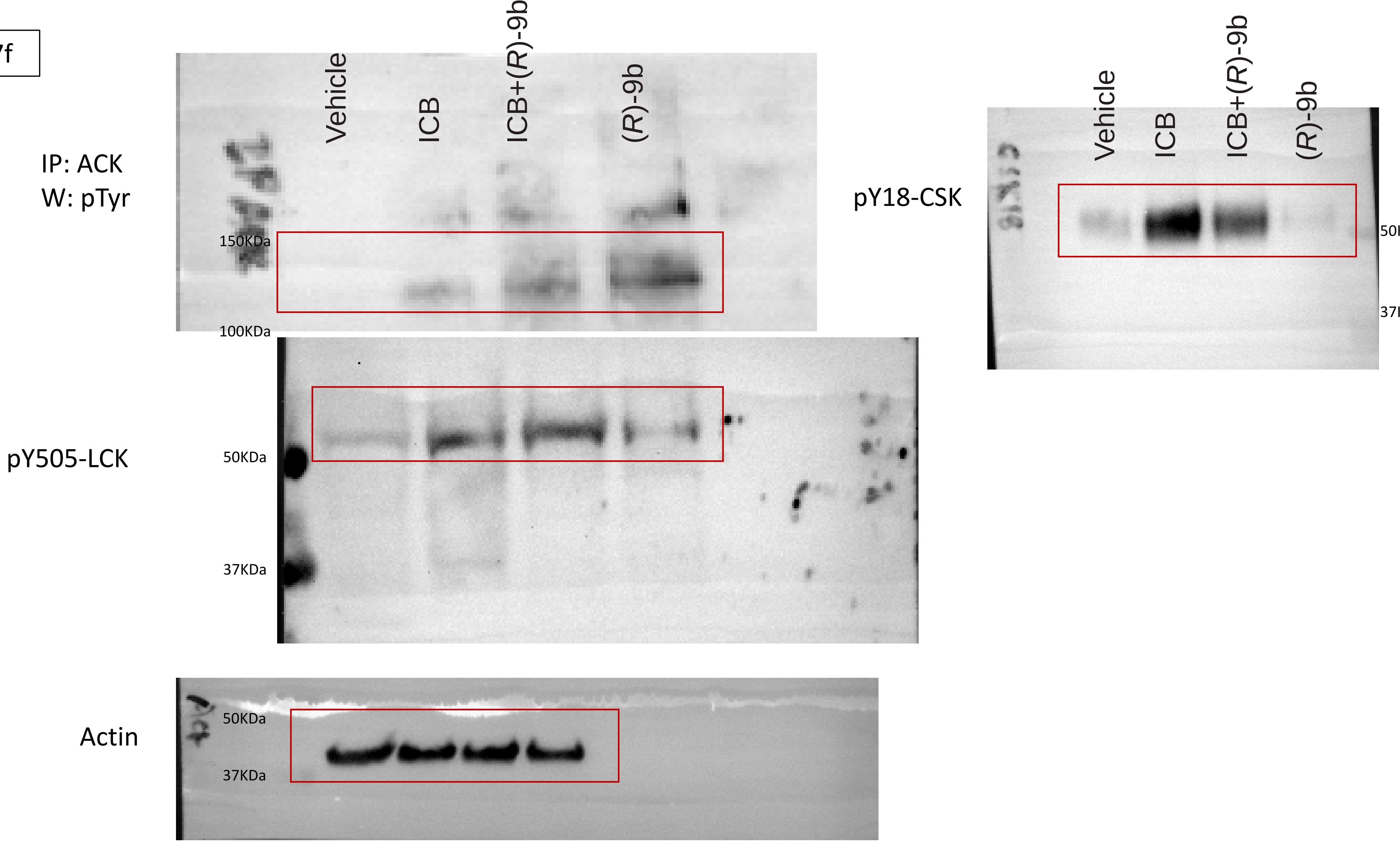

IP: ACK
W: pTyr
Vehicle
ICB
ICB+(R)-9b
(R)-9b
150KDa
100KDa
pY505-LCK
50KDa
37KDa
Actin
50KDa
37KDa
pY18-CSK
Vehicle
ICB
ICB+(R)-9b
(R)-9b

Fig 7g

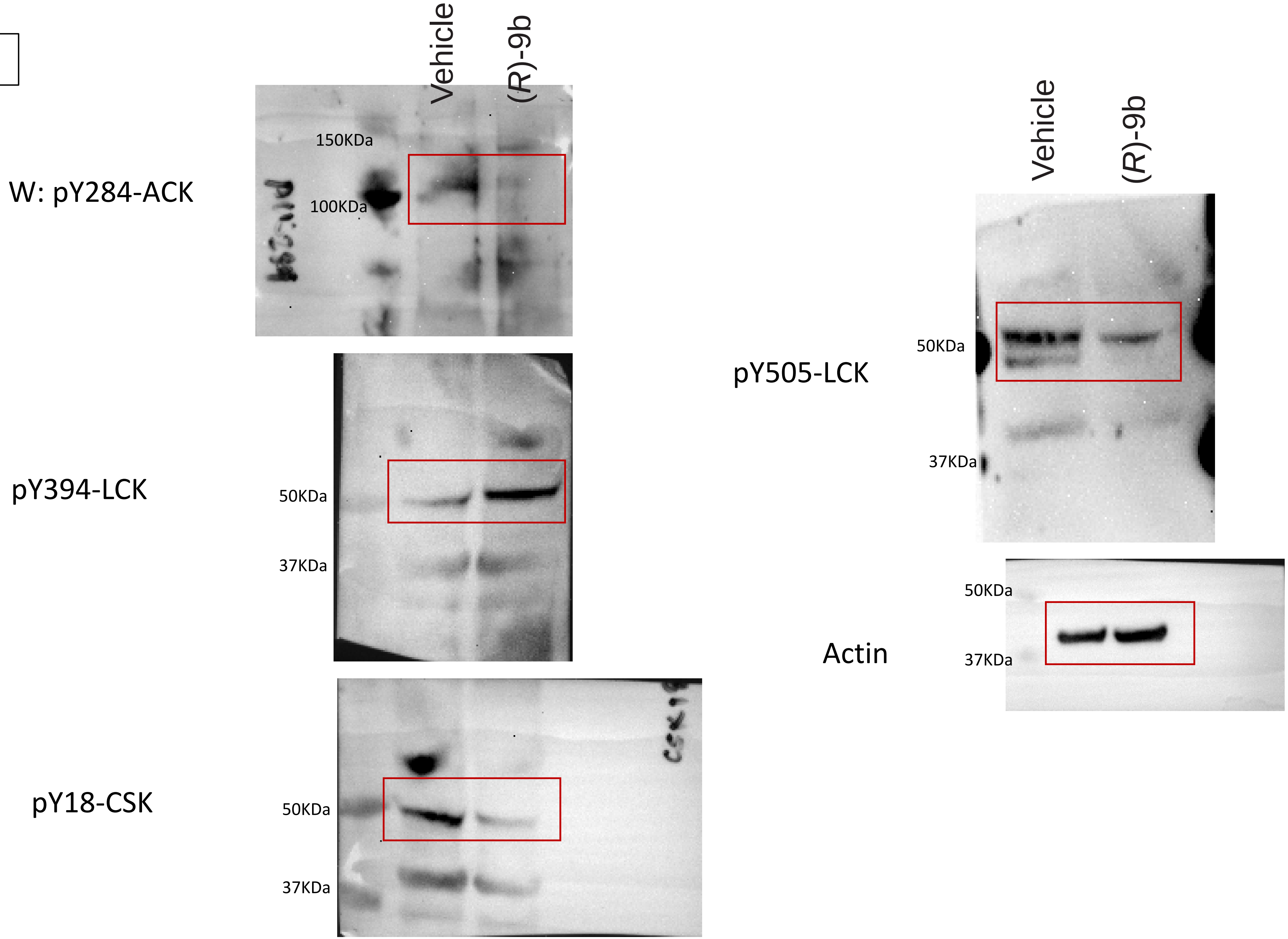
Vehicle
(R)-9b
150KDa
100KDa
W: pY284-ACK
pY394-LCK
50KDa
37KDa
pY18-CSK
50KDa
37KDa
Vehicle
(R)-9b
pY505-LCK
50KDa
37KDa
Actin
50KDa
37KDa
