# Supplementary material for: Inhibiting ACK1-mediated phosphorylation of C-terminal Src kinase counteracts prostate cancer immune checkpoint blockade resistance
Source: Nat Commun. 2022 Nov 14;13:6929. doi: 10.1038/s41467-022-34724-5 (PMC9663509; doi:10.1038/s41467-022-34724-5)

Supplementary Fig. 1c

Genotype confirmed by tail PCR

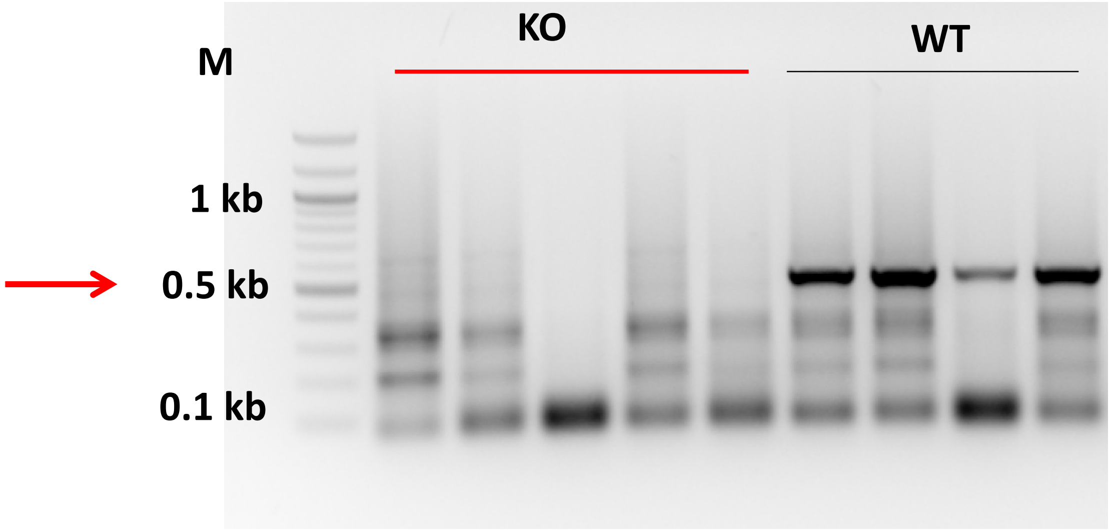

WT – Intact Ack1   KO – Ack1 knockout

Supplementary Fig. 2

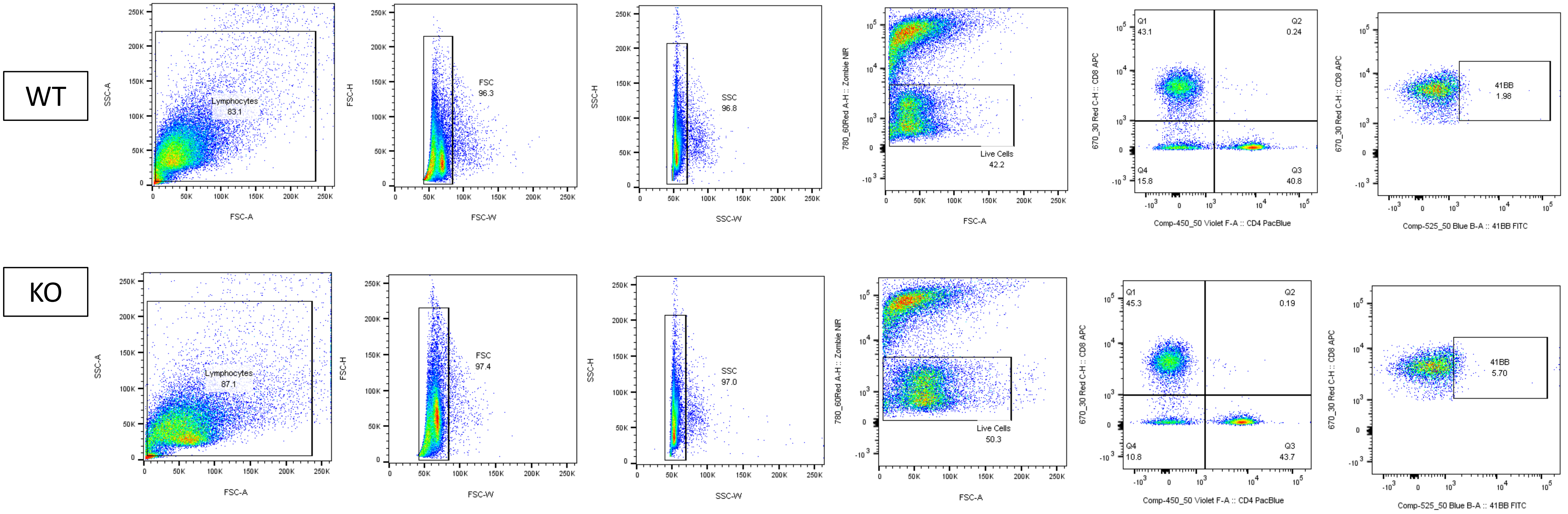

Flow cytometric analysis of CD137 on CD8 gated population isolated from splenocytes of WT and *Ack1* KO mice injected with TRAMP-C2 cells.

Supplementary Fig. 4a

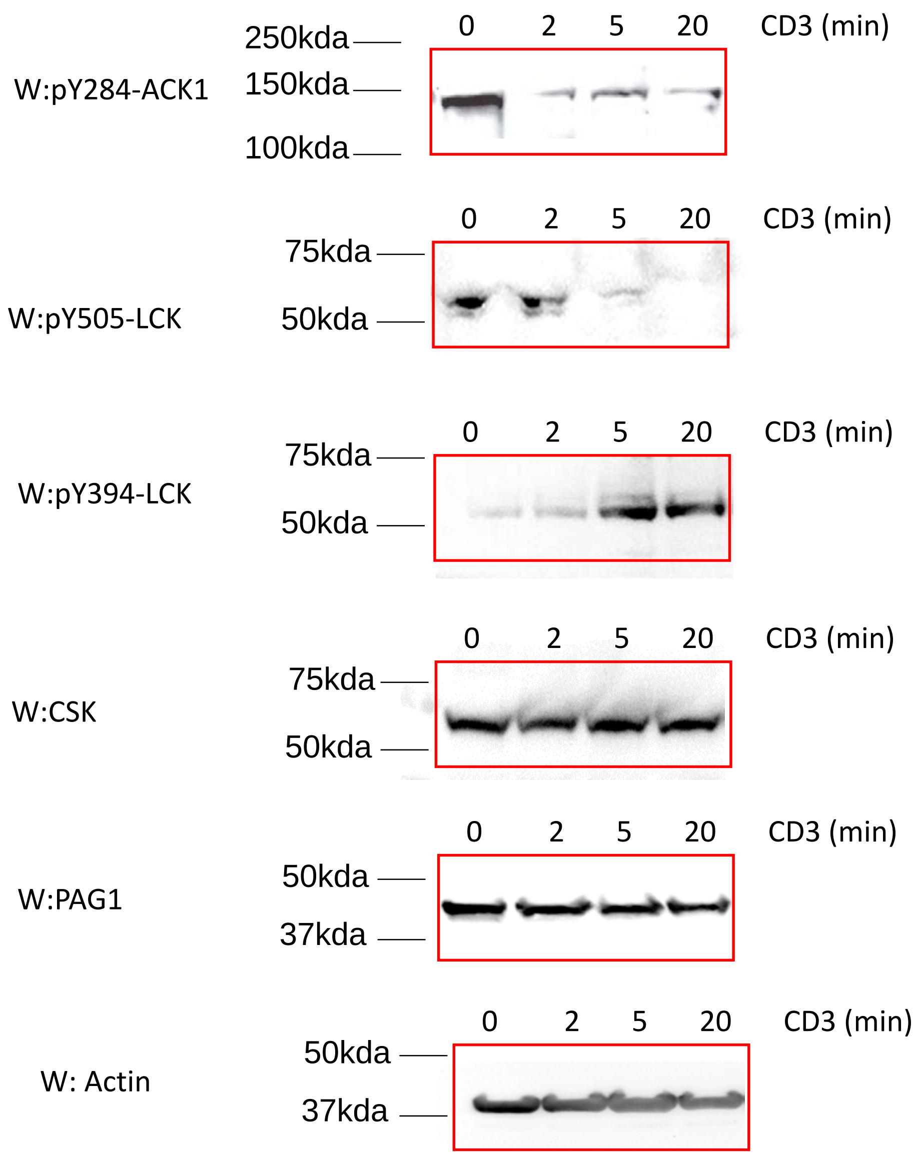

Supplementary Fig. 4b

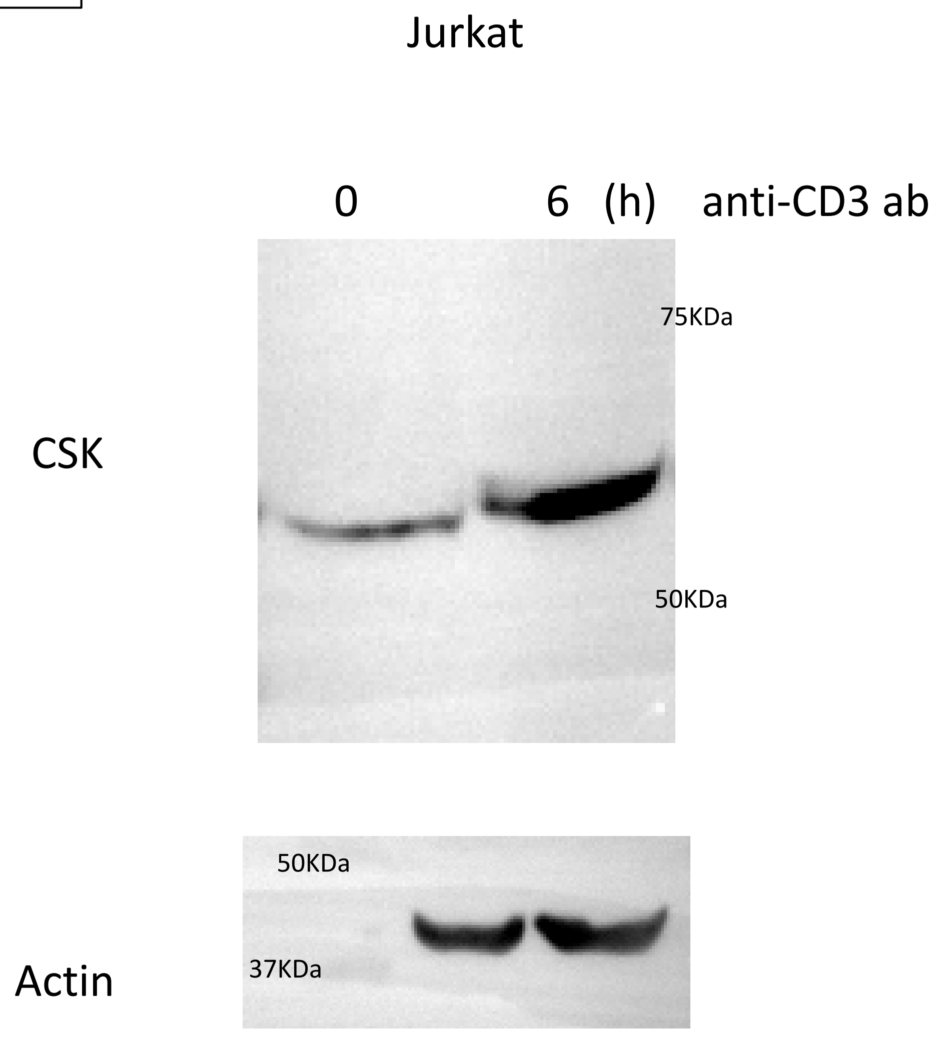

Supplementary Fig. 4c

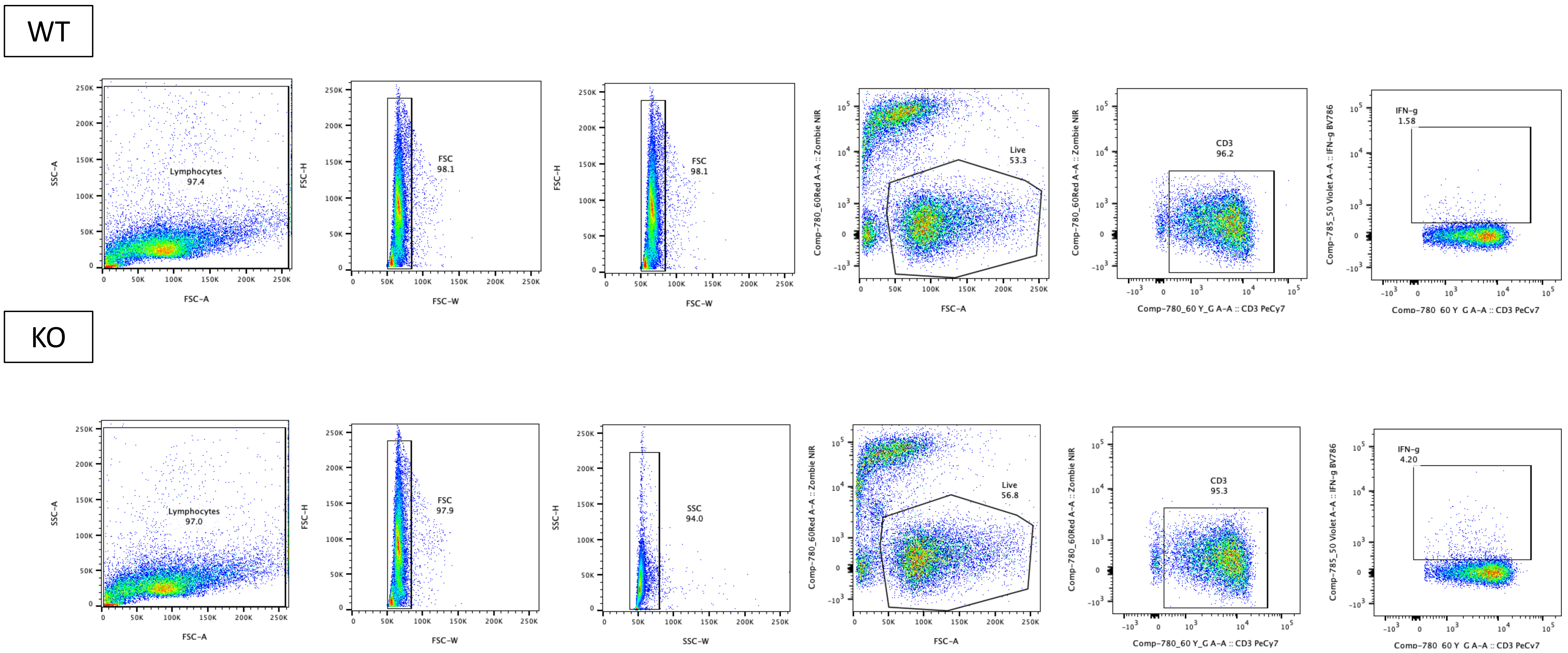

Flow cytometric analysis of IL-2 levels on CD8 gated population isolated from splenocytes of WT and *Ack1* KO mice

Supplementary Fig. 4d

WT

KO

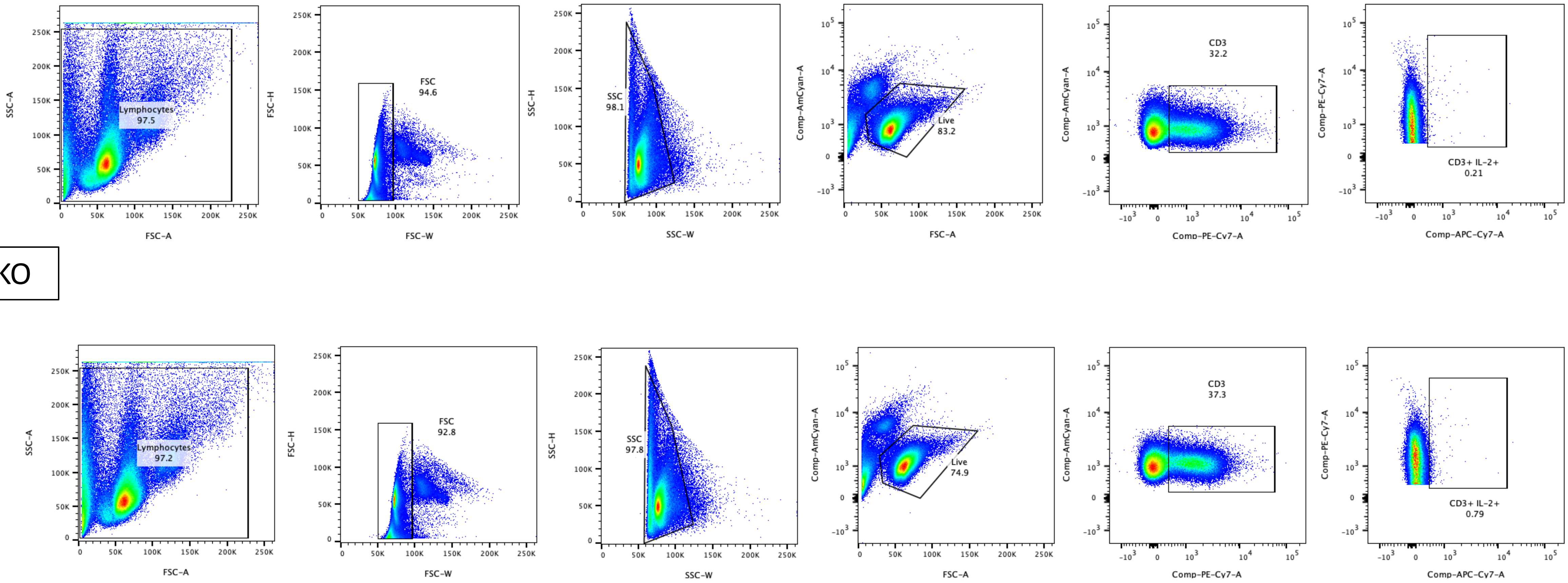

Flow cytometric analysis of IL-2 levels on CD8 gated population isolated from splenocytes of WT and *Ack1* KO mice

Supplementary Fig. 6a

W: pY18-CSK

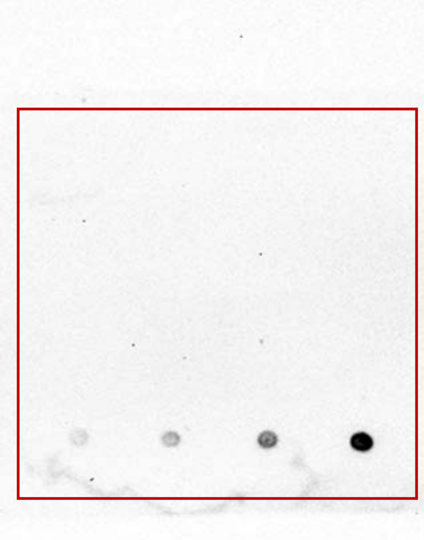

pY37-H2B peptide

non-phosphopeptide to CSK

pY18-CSK phosphopeptide

1 5 20 40 nM of peptide

Ponceau staining

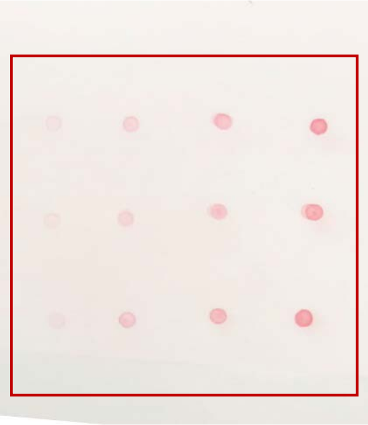

Supplementary Fig. 6c

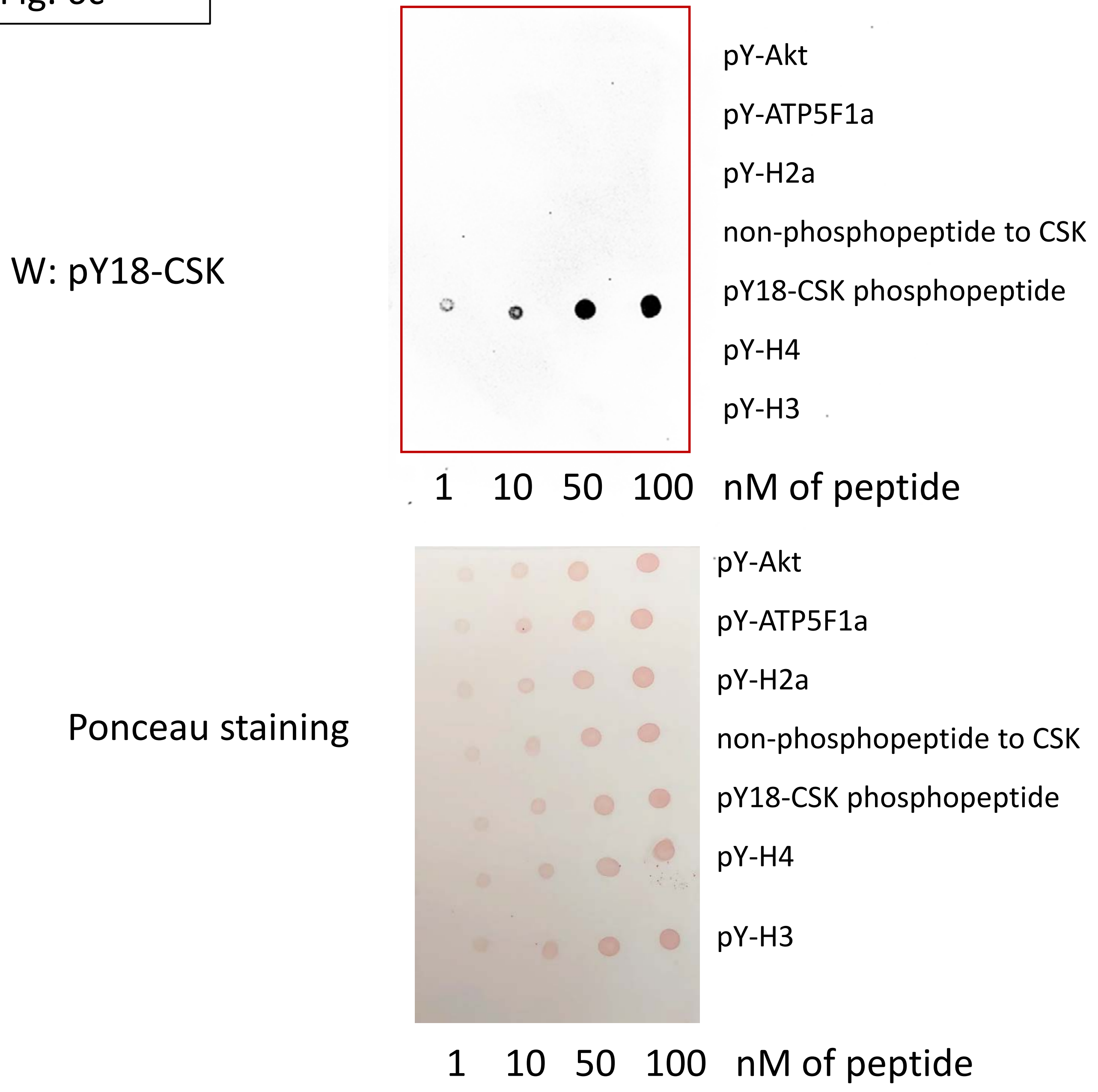

Supplementary Fig. 6d

pY18-CSK

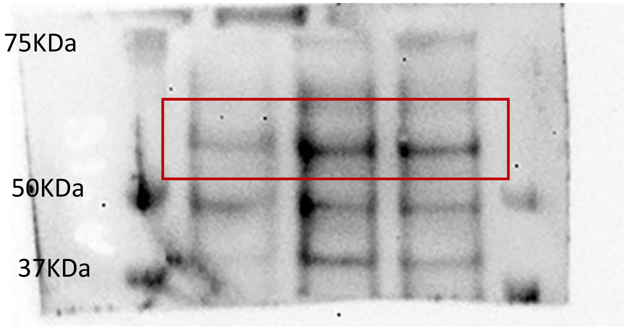

1 2.5 3.5 Protease inhibitor cocktail (ul)

Actin

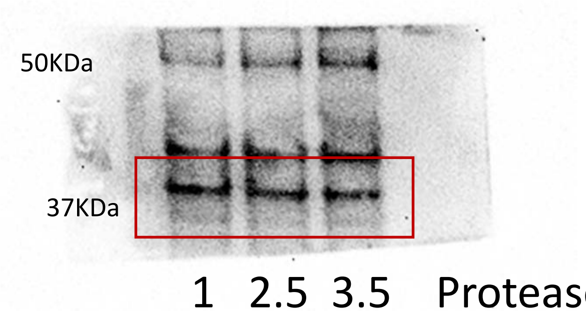

1 2.5 3.5 Protease inhibitor cocktail (ul)

Supplementary Fig. 7c

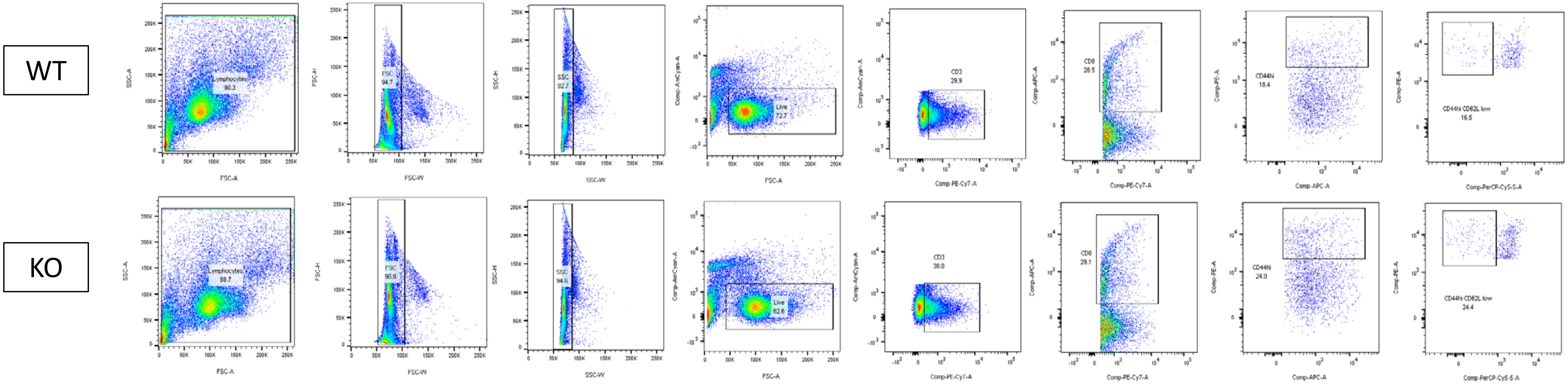

Flow cytometric analysis of CD44^{hi}/CD62L^{low} on CD8 gated population isolated from splenocytes of WT and *Ack1* KO mice injected with TRAMP-C2 tumors

Supplementary Fig. 7d

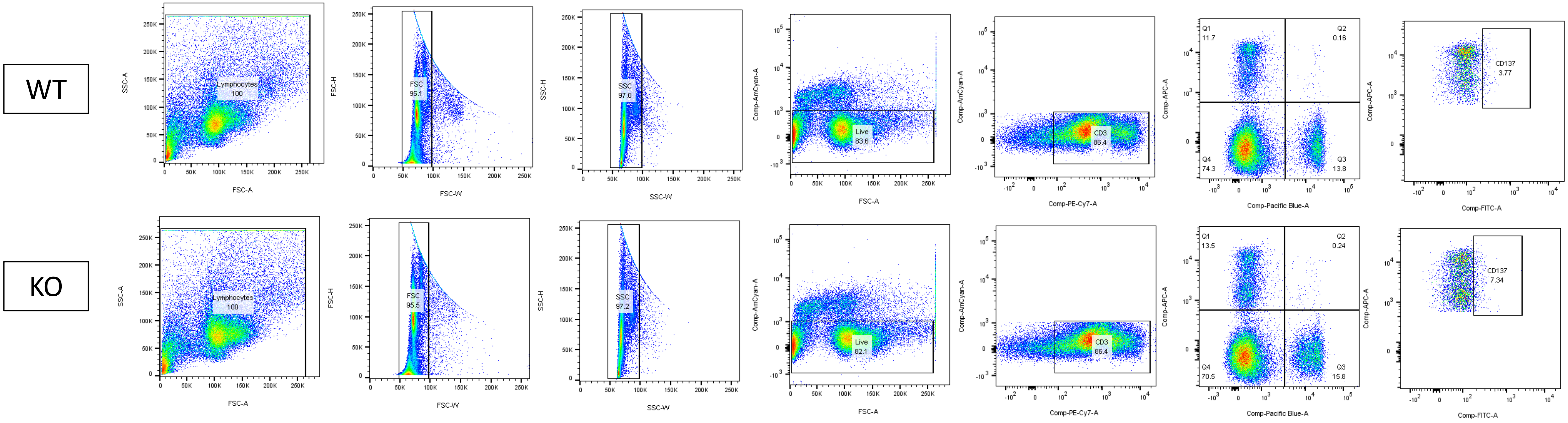

Flow cytometric analysis of CD137 on CD8 gated population isolated from splenocytes of WT and *Ack1* KO mice injected with TRAMP-C2 tumors.

Supplementary Fig. 7e

WT

KO

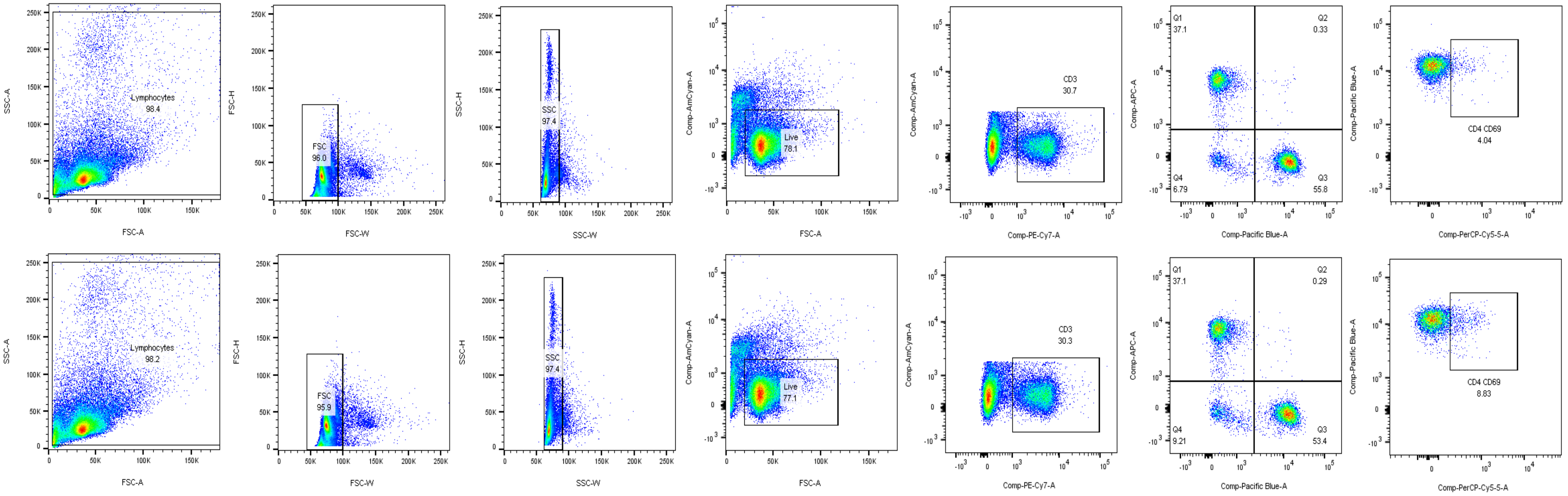

Flow cytometric analysis of CD69 on CD4 gated population isolated from splenocytes of WT and *Ack1* KO mice injected with TRAMP-C2 tumors.

Supplementary Fig. 7f

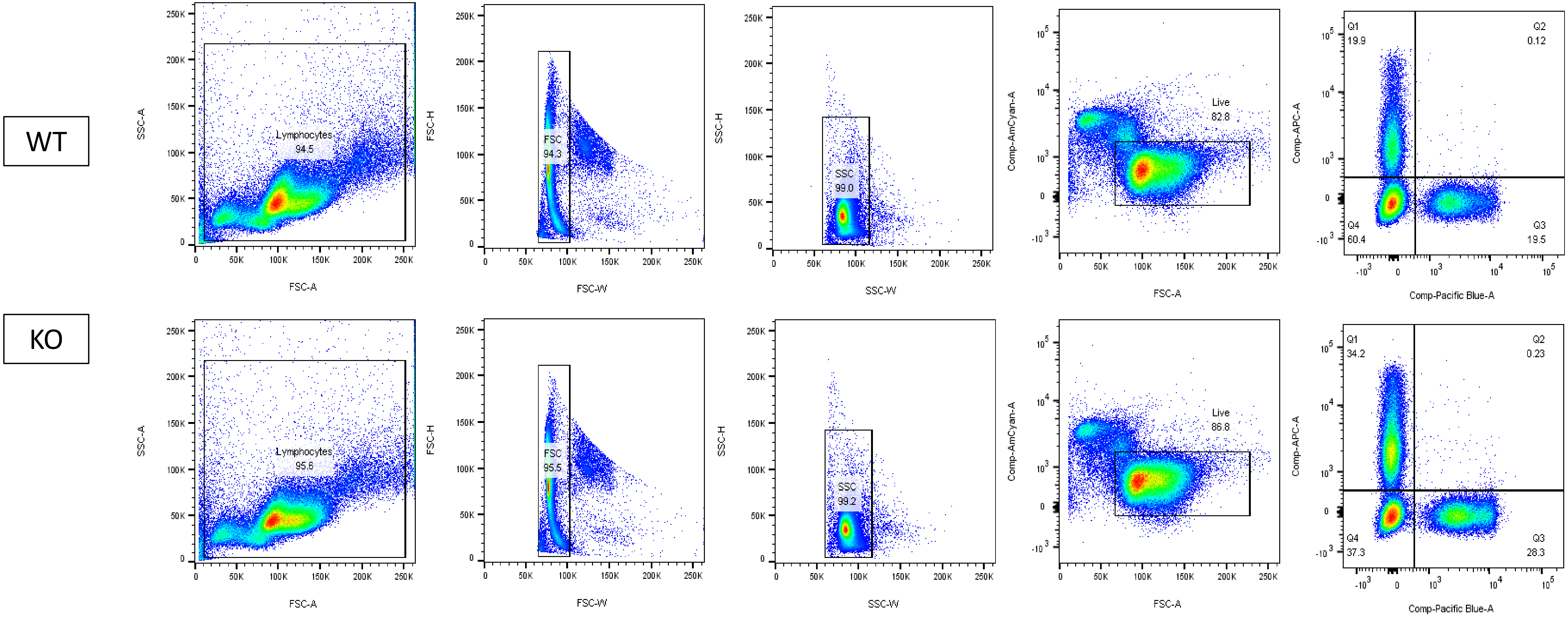

Flow cytometric analysis of CD4 and CD8 population in lymph nodes drained from WT and *Ack1* KO mice implanted with TRAMP-C2 tumors.

Supplementary Fig. 8a

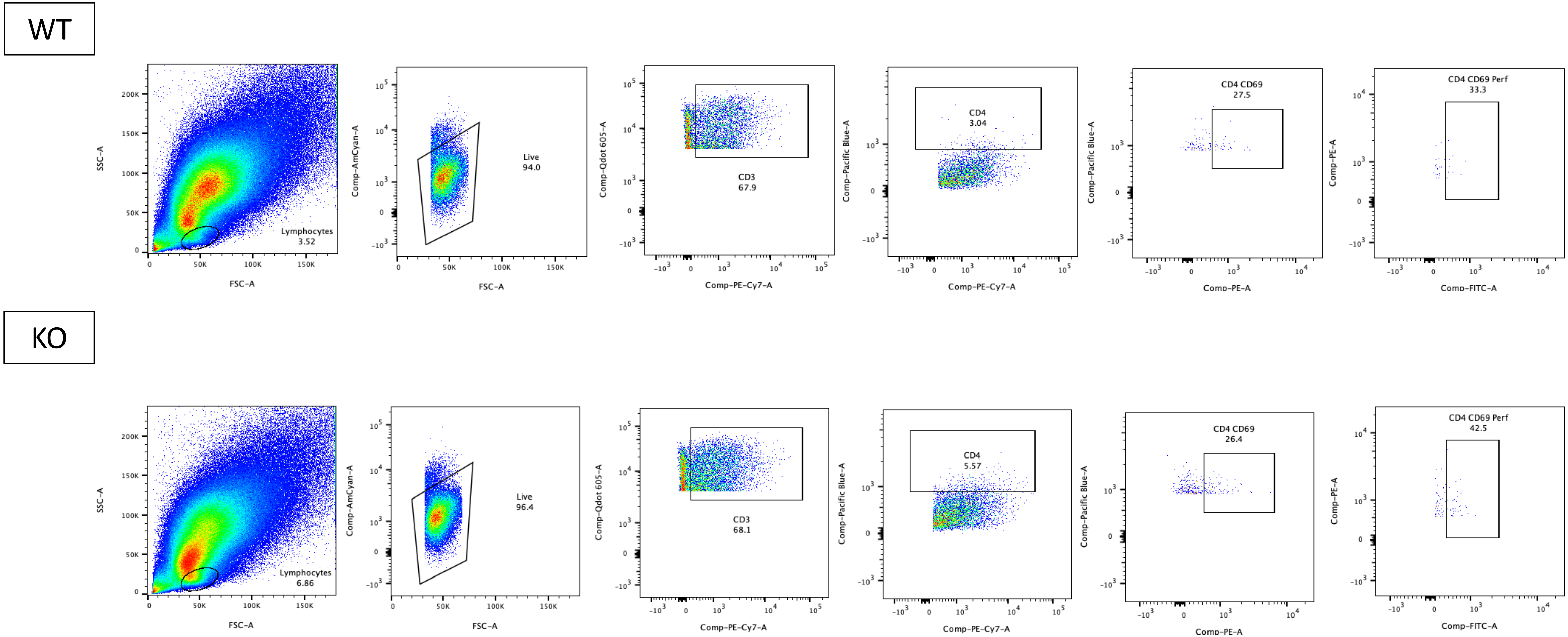

Flow cytometric analysis of Perforin expressing CD4+CD69+ cells in TILS from TRAMP-C2 tumors injected in WT and *Ack1* KO mice.

Supplementary Fig. 8b

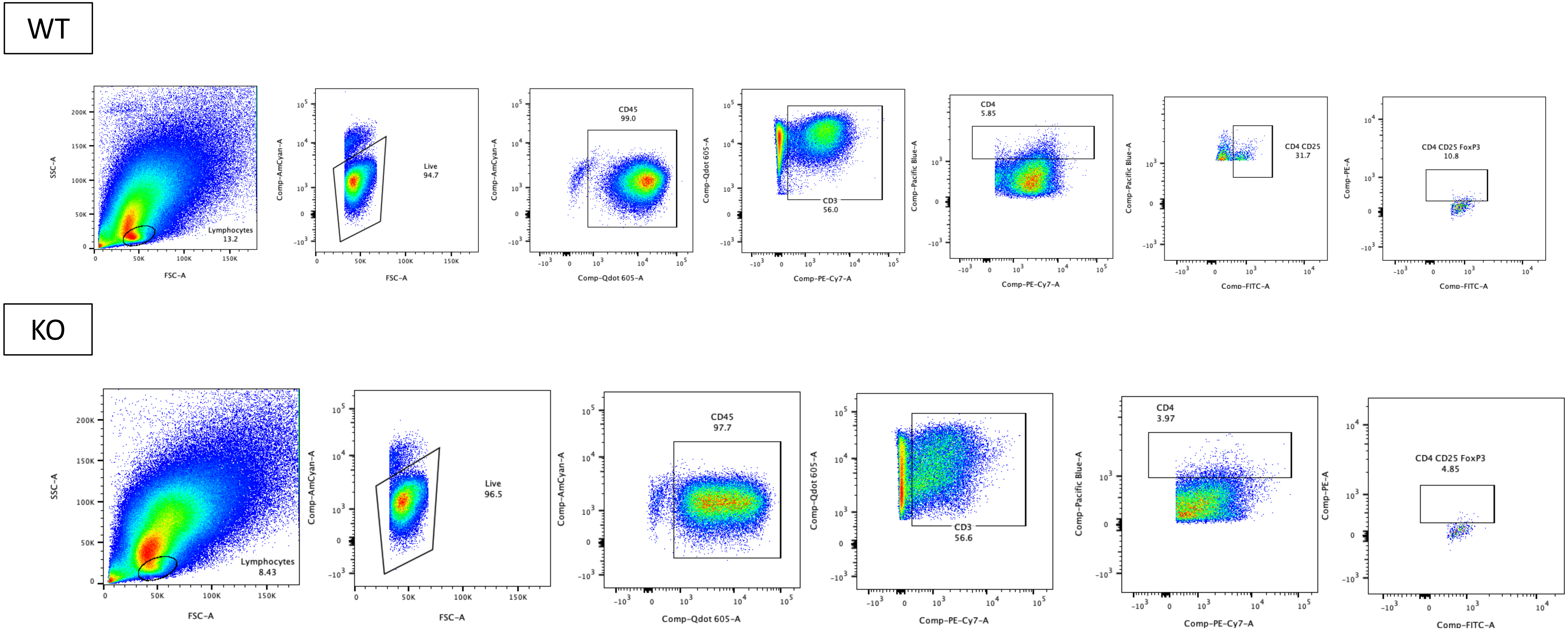

Flow cytometric analysis of Tregs in TILS from TRAMP-C2 tumors injected in WT and *Ack1* KO mice.

Supplementary Fig. 8c

WT

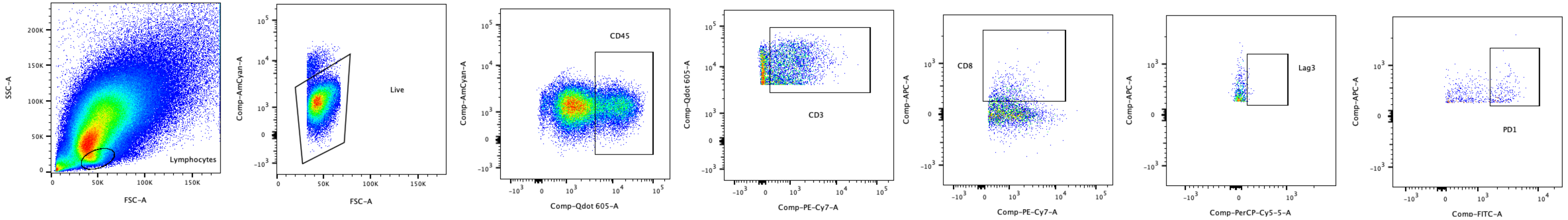

KO

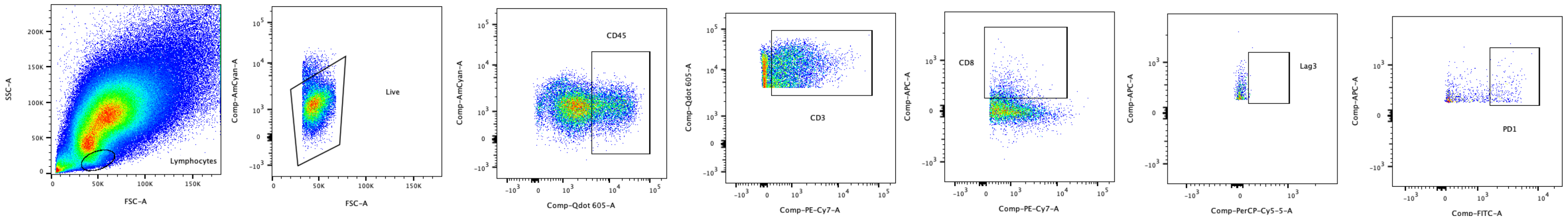

Flow cytometric analysis of exhaustion markers on CD8 gated population in TILS from TRAMP-C2 tumors injected in WT and *Ack1* KO mice.

Supplementary Fig. 9c

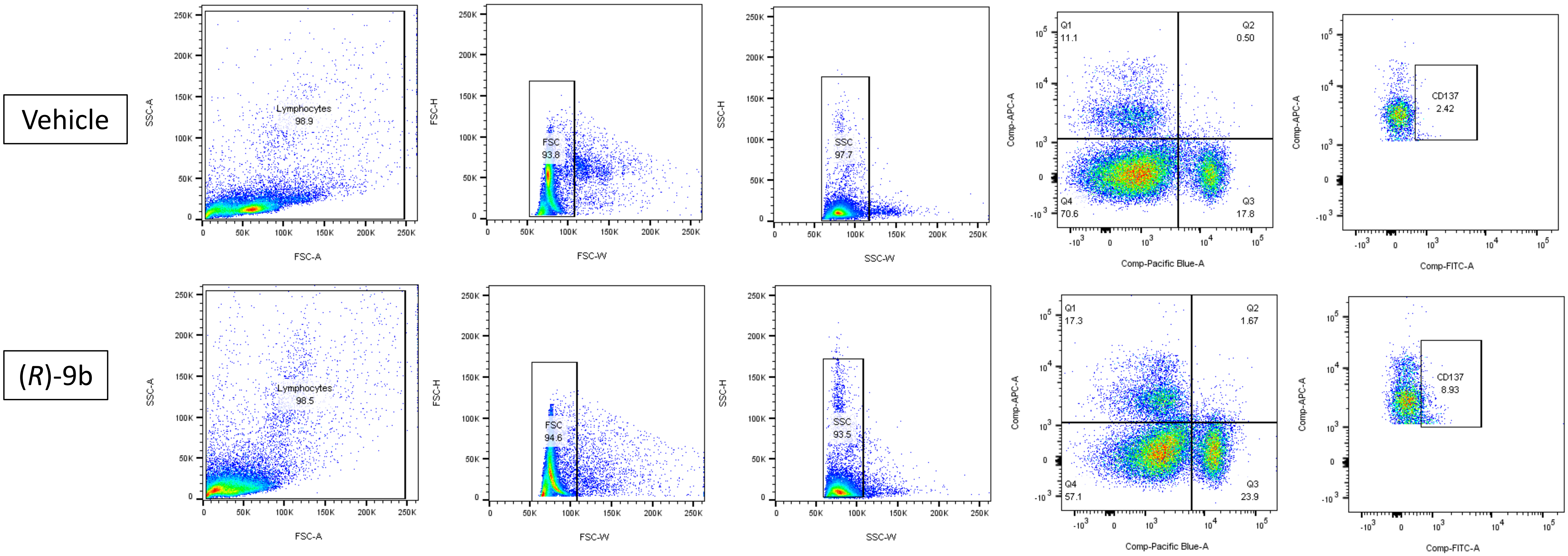

Flow cytometric analysis of CD137 on CD8 gated population isolated from splenocytes of vehicle and (*R*)-**9b** injected mice with TRAMP-C2 tumors.

Supplementary Fig. 9d

Vehicle

(*R*)-9b

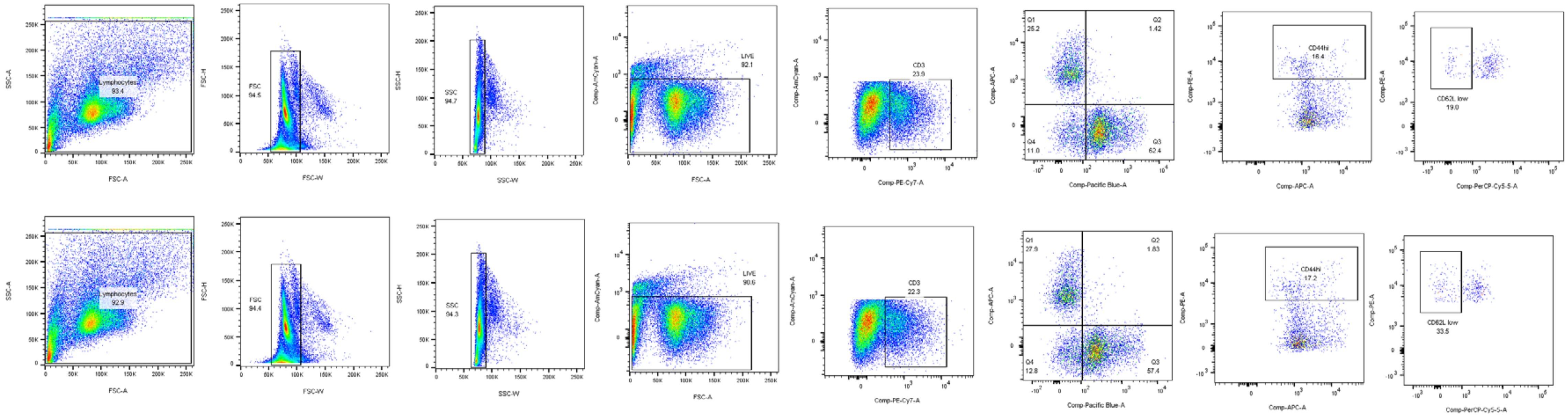

Flow cytometric analysis of CD44^{hi}/CD62L^{low} on CD8 gated population isolated from splenocytes of vehicle and (*R*)-**9b** injected mice with TRAMP-C2 tumors.

Supplementary Fig. 10a

Vehicle

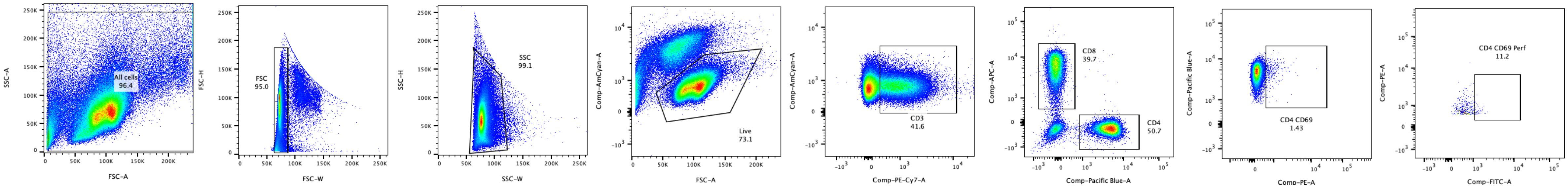

(*R*)-9b

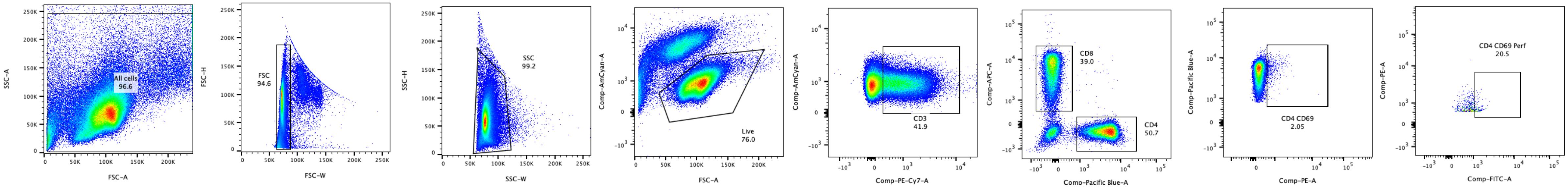

Flow cytometric analysis of Perforin expressing CD4+CD69+ cells from splenocytes of vehicle and (*R*)-**9b** injected mice with TRAMP-C2 tumors.

Supplementary Fig. 10b

Vehicle

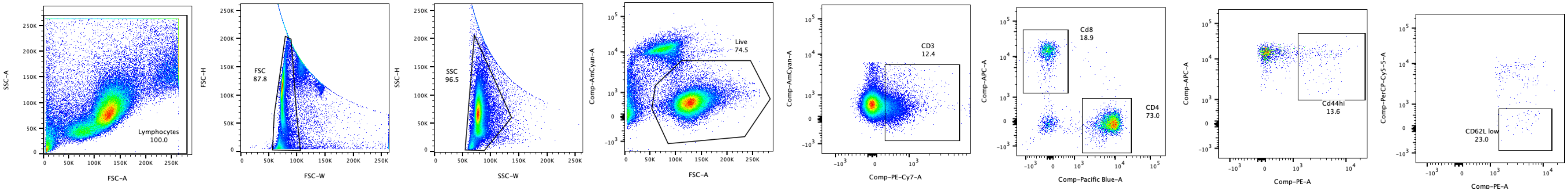

(*R*)-9b

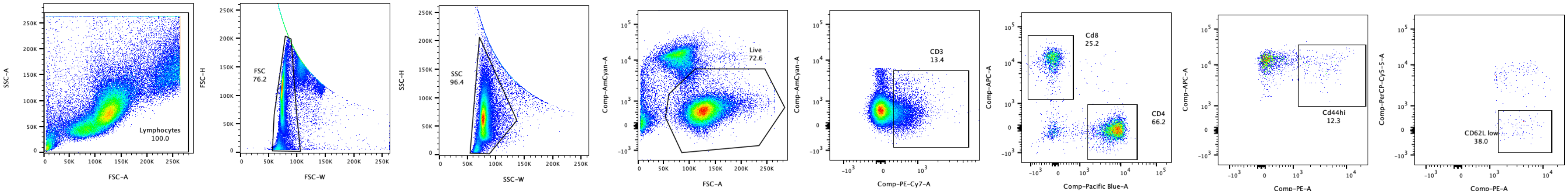

Flow cytometric analysis of CD44^{hi}/CD62L^{low} on CD8 gated population isolated from lymph node of vehicle and (*R*)-**9b** injected mice with TRAMP-C2 tumors.

Supplementary Fig. 10c

Vehicle

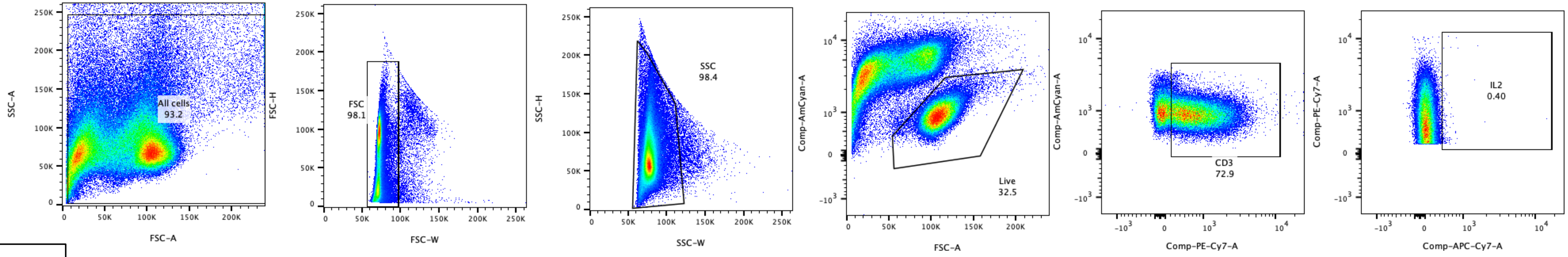

(*R*)-9b

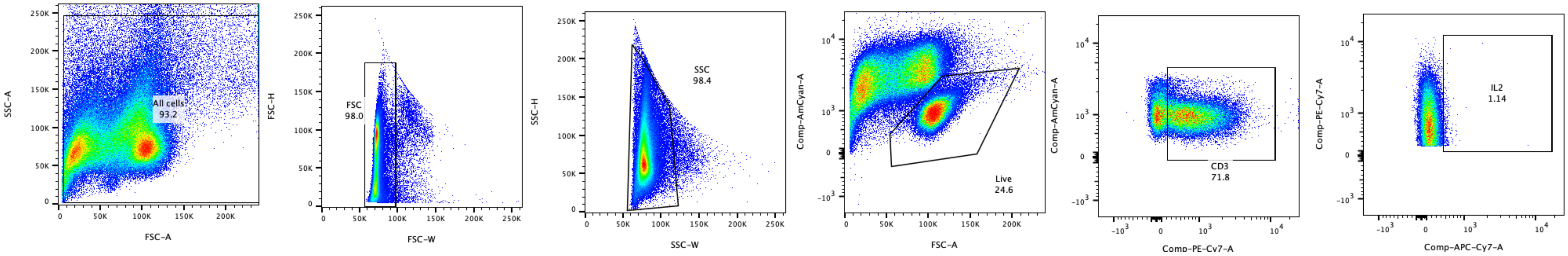

Flow cytometric analysis of IL-2 expressing CD3 population from isolated splenocytes of vehicle and (*R*)-**9b** injected mice with TRAMP-C2 tumors.

Supplementary Fig. 10d

Vehicle

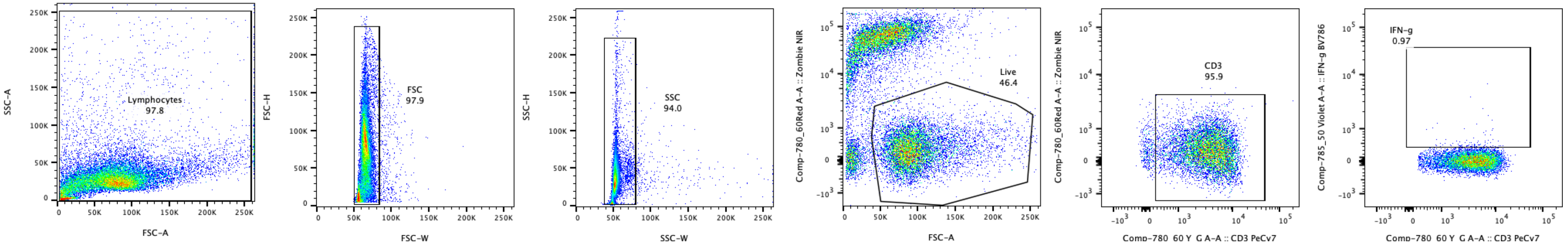

(*R*)-9b

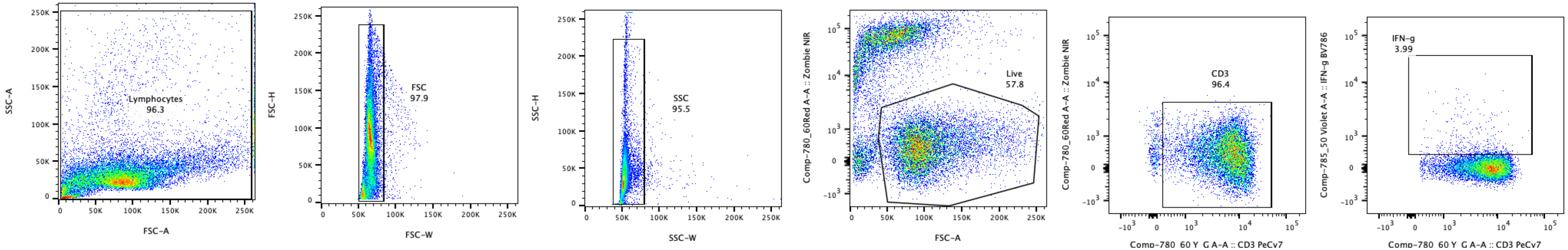

Flow cytometric analysis of IFN-***γ*** expressing CD3 population from isolated splenocytes of vehicle and (*R*)-**9b** injected mice with TRAMP-C2 tumors.

Supplementary Fig. 11a

Vehicle

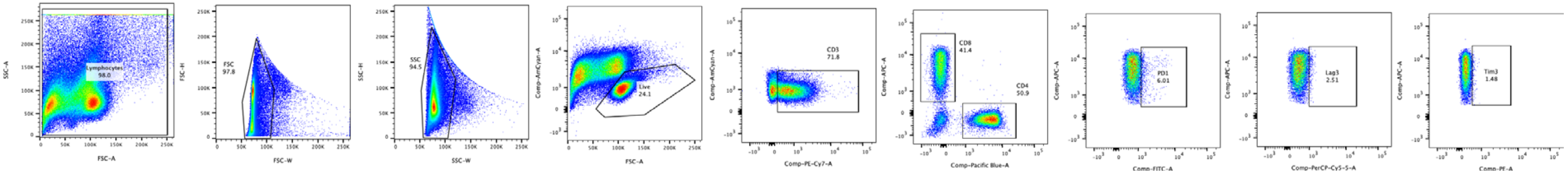

(*R*)-9b

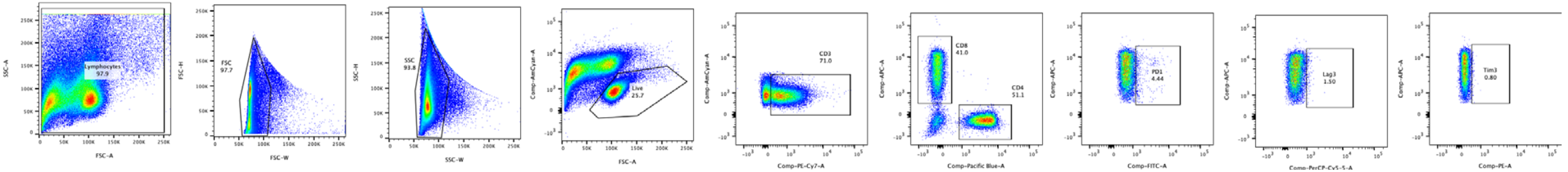

Flow cytometric analysis of exhaustion markers on CD8 gated population from isolated splenocytes of vehicle and (*R*)-**9b** injected mice with TRAMP-C2 tumors.

Supplementary Fig. 11b

Vehicle

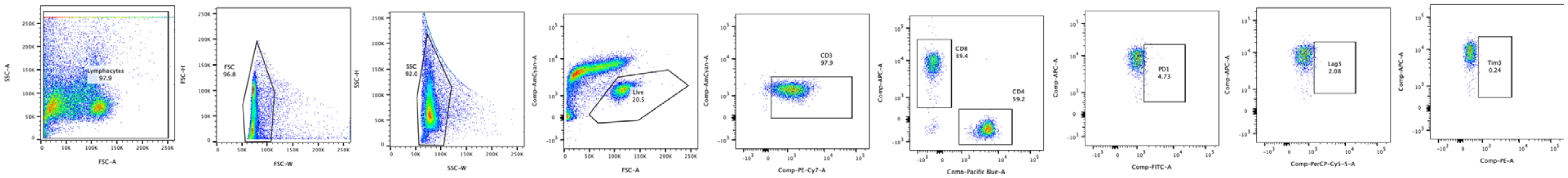

(*R*)-9b

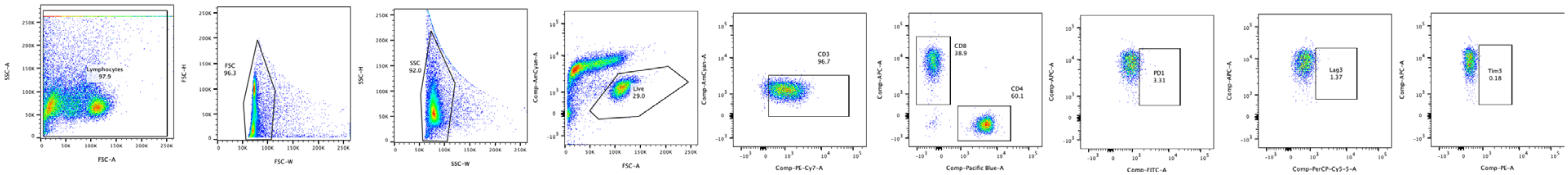

Flow cytometric analysis of exhaustion markers on CD8 gated population from isolated draining lymph nodes of vehicle and (*R*)-**9b** injected mice with TRAMP-C2 tumors.

Supplementary Fig. 12a

WT

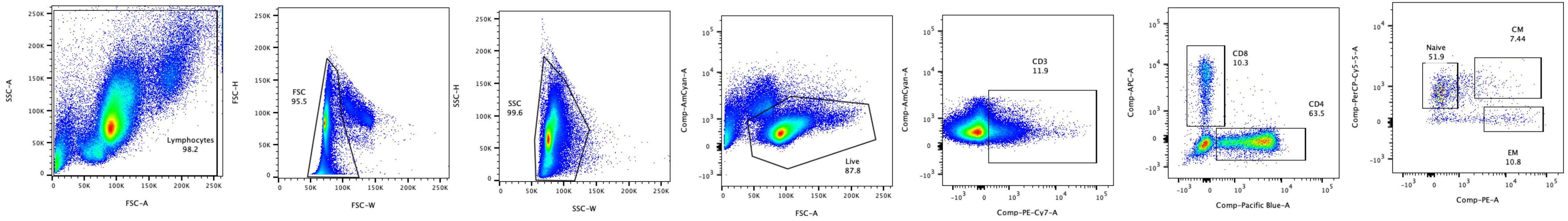

KO

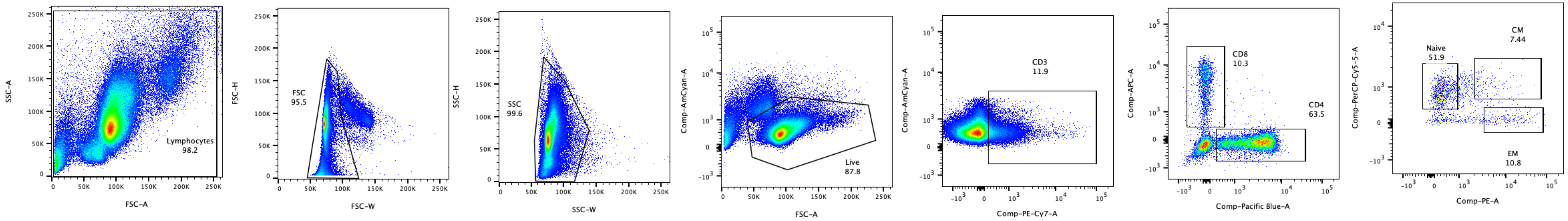

Flow cytometric analysis of naïve, central memory (CM), effector memory (EM) CD8 T cells in splenocytes isolated from TRAMP-C2 tumors injected in WT and *Ack1* KO mice.

Supplementary Fig. 12b

Vehicle

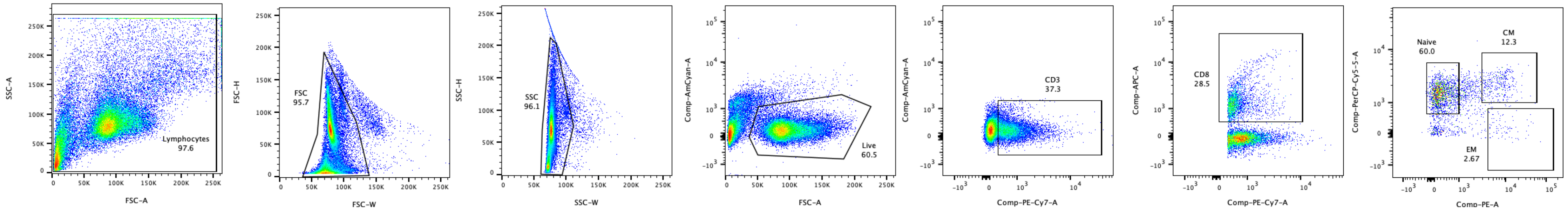

(*R*)-9b

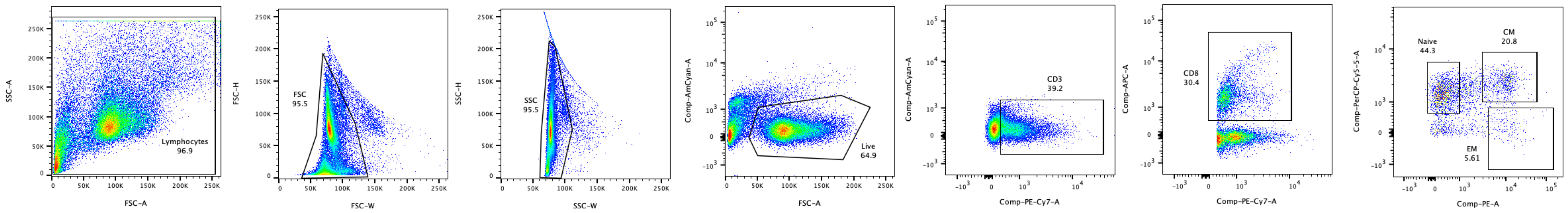

Flow cytometric analysis of naïve, central memory (CM), effector memory (EM) CD8 T cells in splenocytes of vehicle and (*R*)-**9b** injected mice with TRAMP-C2 tumors.

Supplementary Fig. 13e

WT

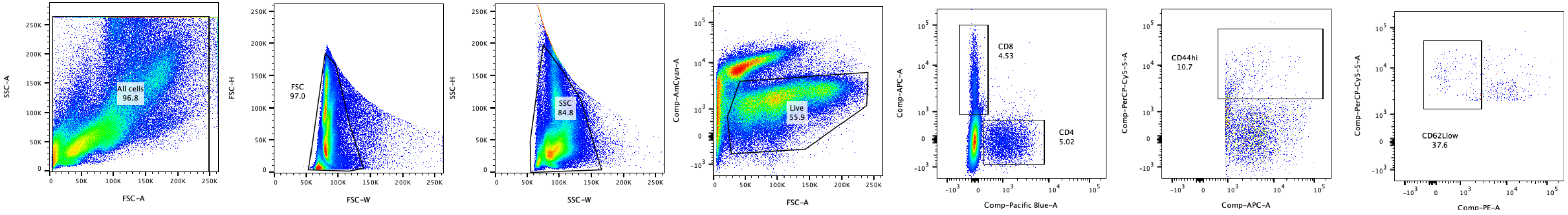

KO

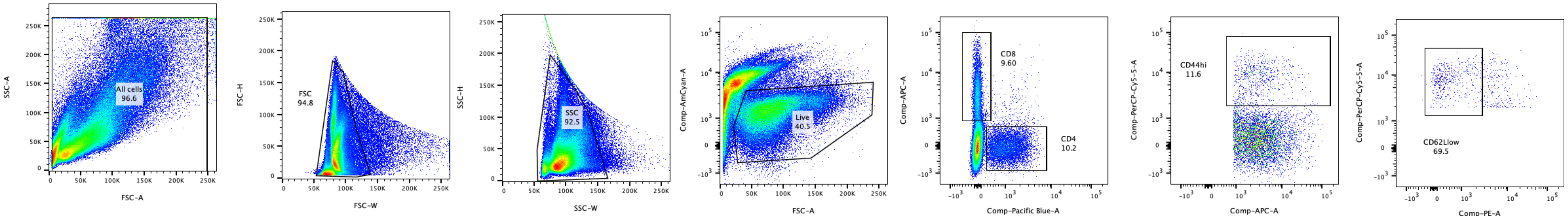

Flow cytometric analysis of $CD44^{hi}/CD62L^{low}$ on CD8 gated population after adoptive transfer T cells from WT and *Ack1* KO mice into TRAMP-C2 tumor bearing NSG mice

Supplementary Fig. 13e

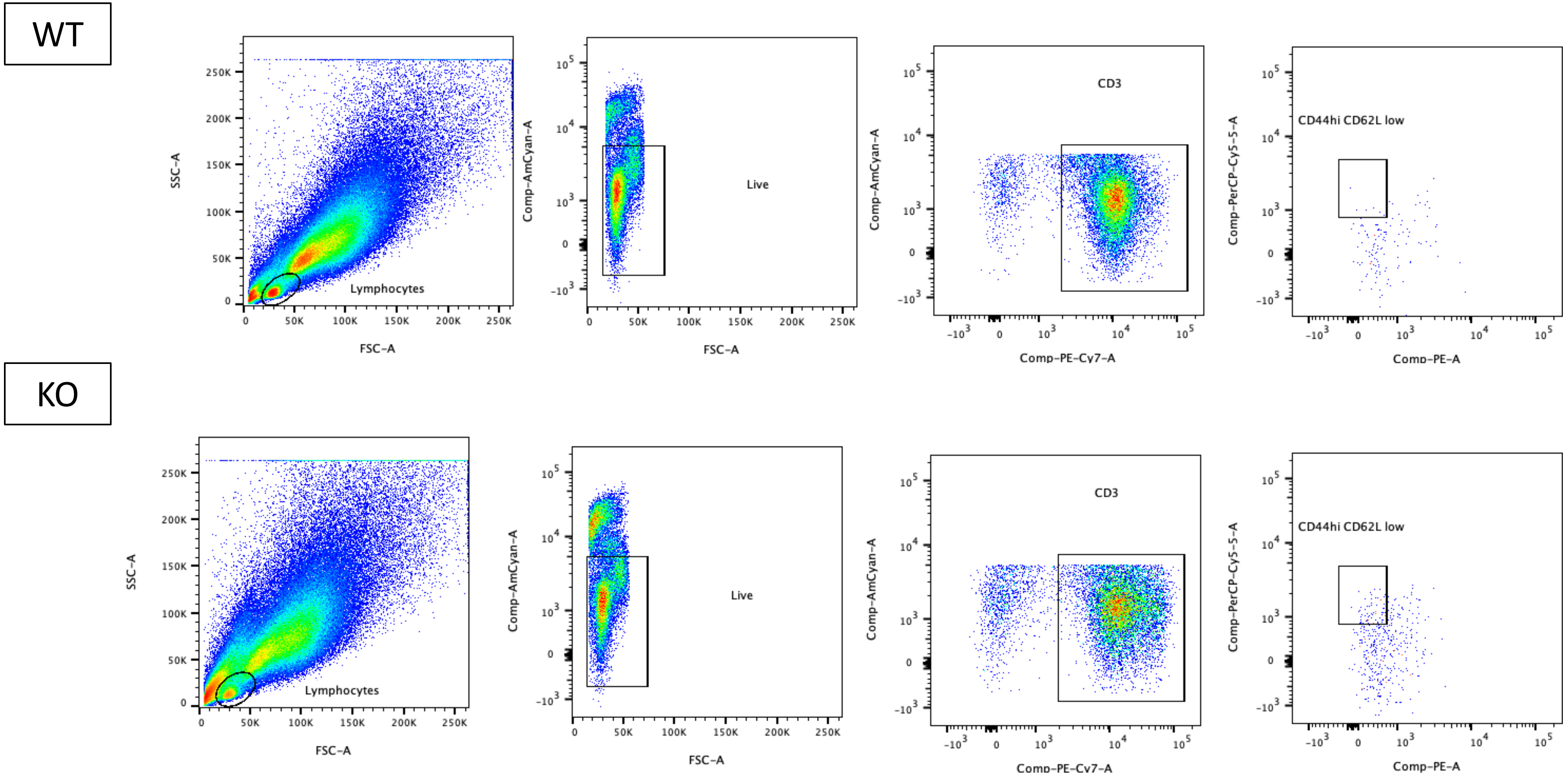

Flow cytometric analysis of CD44^{hi}/CD62L^{low} on CD8 gated population in the TILS after adoptive transfer T cells from WT and *Ack1* KO mice into TRAMP-C2 tumor bearing NSG mice

Supplementary Fig. 13f

Control tetramer

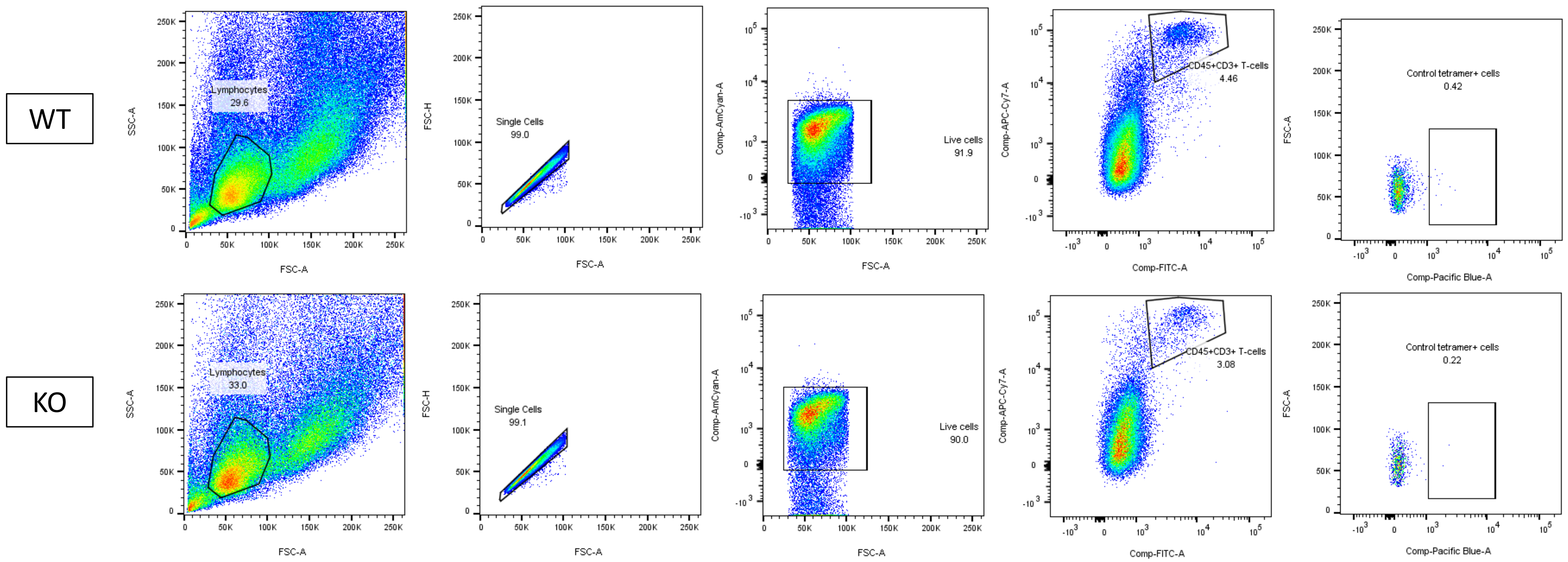

Scatter plot of the control tetramer stained splenocytes assessed by flow cytometry.

Supplementary Fig. 15

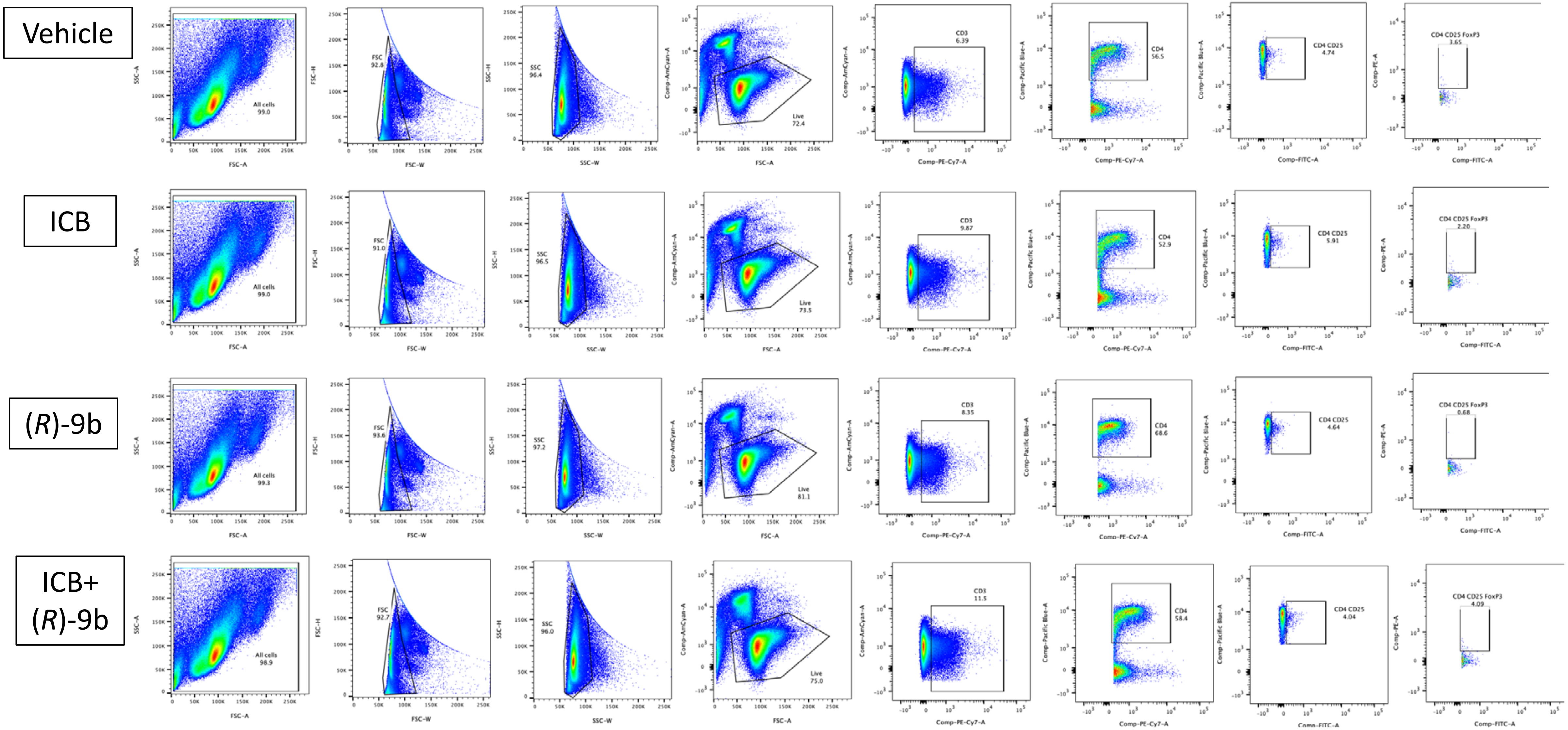

Flow cytometric analysis of Tregs in TILS from TRAMP-C2 tumors injected in C57BL/6 mice treated with vehicle, ICB antibodies, (*R*)-9b and combination.

Supplementary Fig. 17c

PBMC

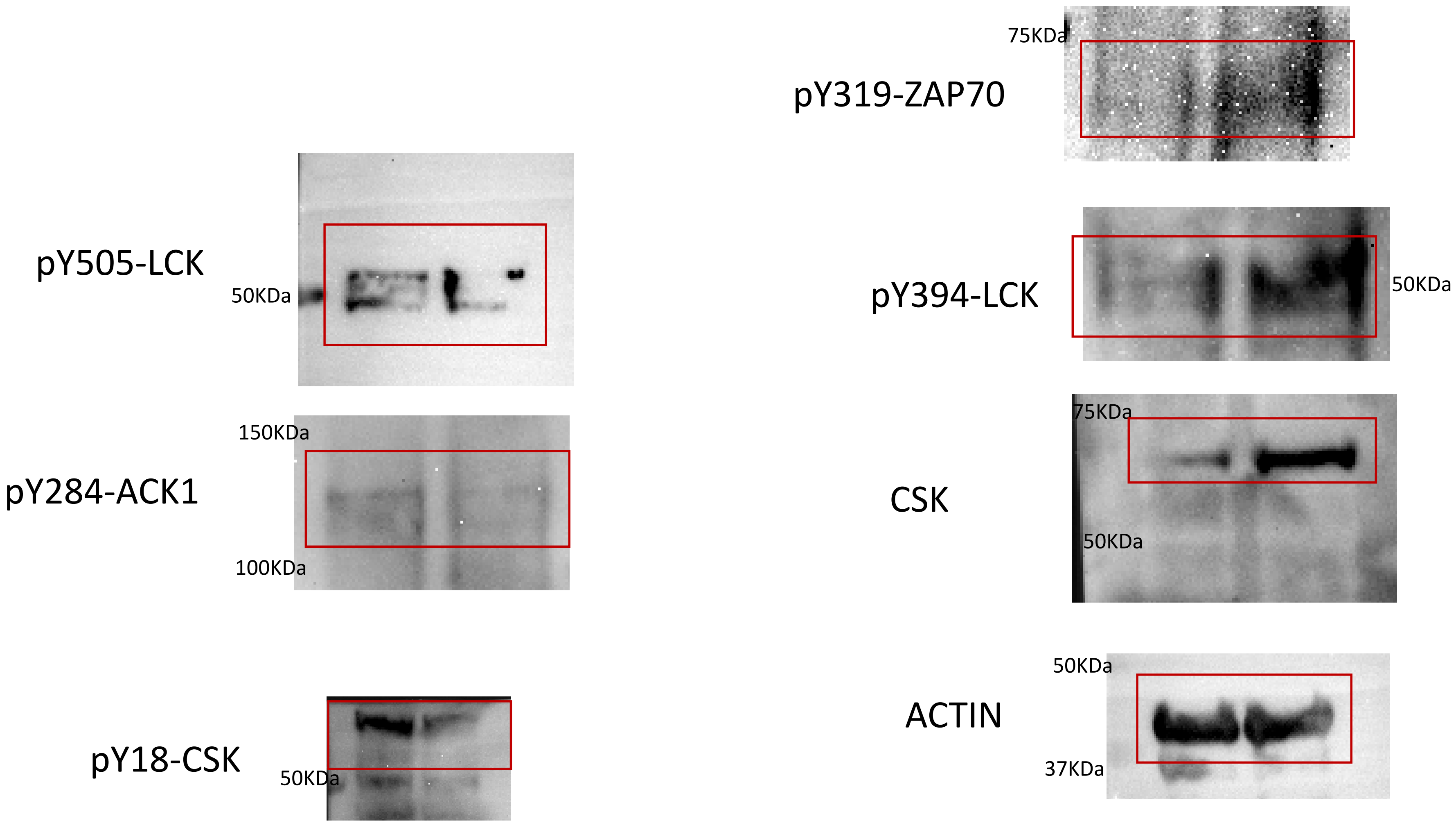

Supplementary Fig. 17e

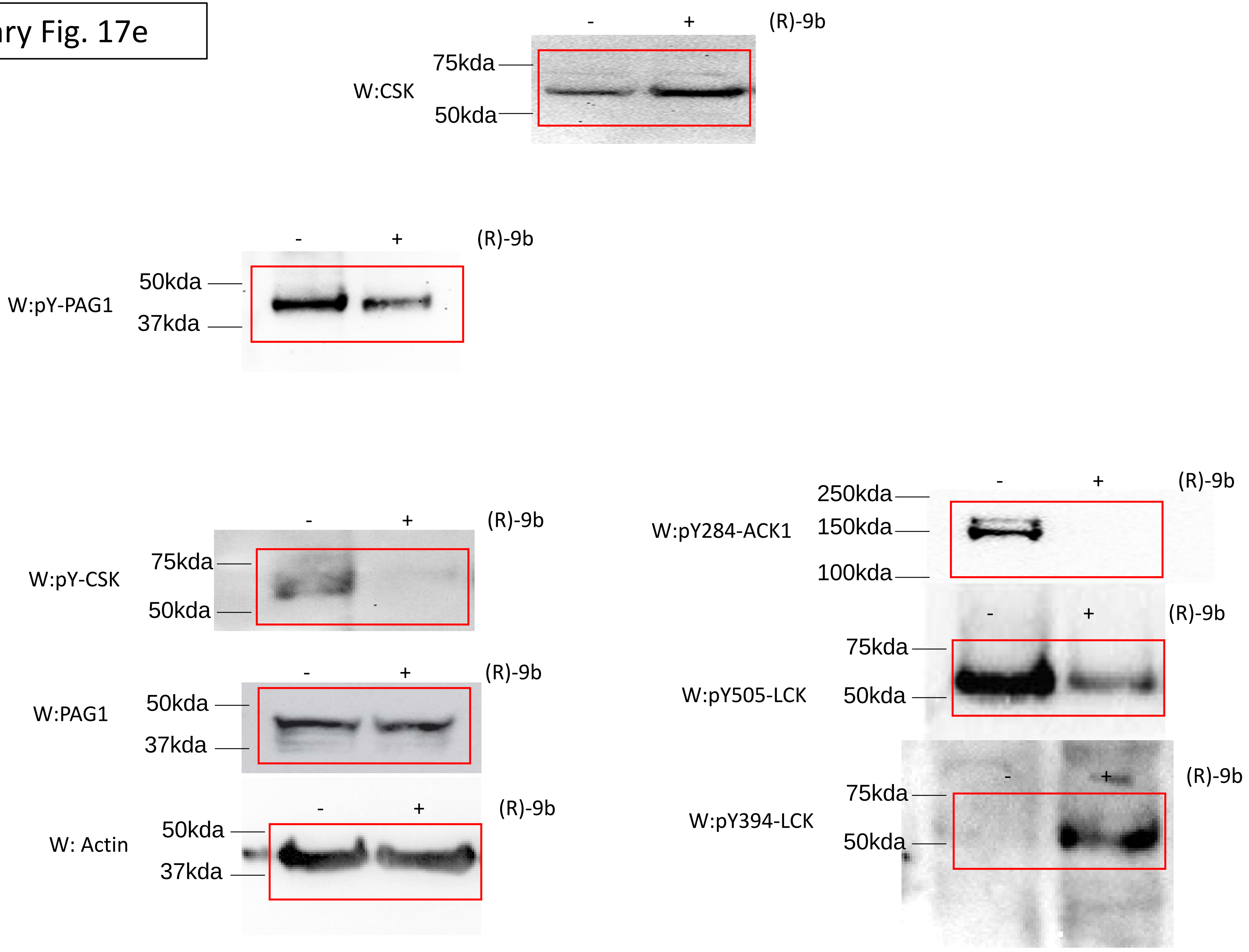

Supplementary Fig. 17f

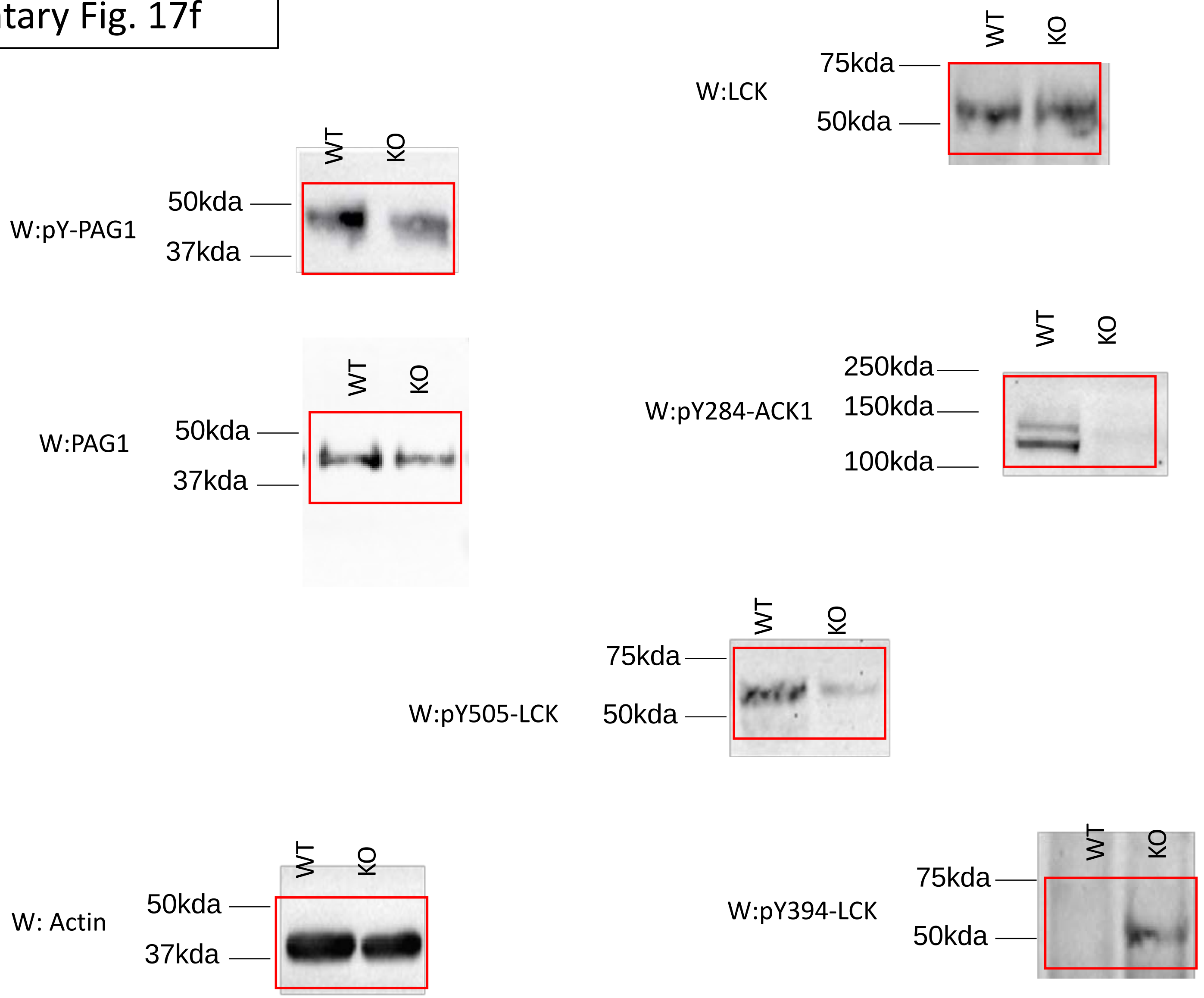

Supplement: Supplementary file 4 — Source Data [file 41467_2022_34724_MOESM4_ESM.zip › 331588_3_related_ms_7010046_rk2r4x.pdf]
